# A Network Pharmacology Approach for Exploring the Mechanisms of *Panax notoginseng* Saponins in Ischaemic Stroke

**DOI:** 10.1155/2021/5582782

**Published:** 2021-08-13

**Authors:** Cong Wang, Hao Chen, Shi-tang Ma, Bin-bin Mao, Yu Chen, Hao-Nan Xu, Hao Yu

**Affiliations:** College of Life and Health Sciences, Anhui Science and Technology University, Fengyang 233100, China

## Abstract

**Background:**

*Panax notoginseng* saponins (PNS) have been deemed effective herb compounds for treating ischaemic stroke (IS) and improving the quality of life of IS patients. This study aimed to investigate the underlying mechanisms of PNS in the treatment of IS based on network pharmacology.

**Methods:**

PNS were identified from the Traditional Chinese Medicine System Pharmacology (TCMSP) database, and their possible targets were predicted using the PharmMapper database. IS-related targets were identified from the GeneCards database, OMIM database, and DisGeNET database. A herb-compound-target-disease network was constructed using Cytoscape, and protein-protein interaction (PPI) networks were established with STRING. GO enrichment and KEGG pathway analysis were performed using DAVID. The binding of the compounds and key targets was validated by molecular docking studies using AutoDock Vina. The neuroprotective effect of TFCJ was substantiated in terms of oxidative stress (superoxide dismutase, glutathione peroxidase, catalase, and malondialdehyde) and the levels of IGF1/PI3K/Akt pathway proteins.

**Results:**

A total of 375 PNS targets and 5111 IS-related targets were identified. Among these targets, 241 were common to PNS, and IS network analysis showed that MAPK1, AKT1, PIK3R1, SRC, MAPK8, EGFR, IGF1, HRAS, RHOA, and HSP90AA1 are key targets of PNS against IS. Furthermore, GO and KEGG enrichment analysis indicated that PNS probably exert therapeutic effects against IS by regulating many pathways, such as the Ras, oestrogen, FoxO, prolactin, Rap1, PI3K-Akt, insulin, PPAR, and thyroid hormone signalling pathways. Molecular docking studies further corroborated the experimental results.The network pharmacology results were further verified by molecular docking and in vivo experiments.

**Conclusions:**

The ameliorative effects of PNS against IS were predicted to be associated with the regulation of the IGF1-PI3K-Akt signalling pathway. Ginsenoside Re and ginsenoside Rb1 may play an important role in the treatment of IS.

## 1. Introduction

Stroke is a common disease worldwide, accounting for 11.59% of all deaths in 2019. Stroke, which accounts for more than 20% of China's total deaths, is the most common cause of death in China [1]. The main categories of stroke are ischaemic stroke (IS), cerebral haemorrhage, and subarachnoid haemorrhage [2]. In China, IS accounts for more than 80% of cases of stroke [1]. IS mainly occurs due to the obstruction of the middle cerebral artery, causing regional ischaemia and hypoxia in brain tissue, leading to DNA damage and cell apoptosis [3]. At present, there are no specific drugs for the clinical treatment of IS. Most of the drugs used for treatment are drugs that protect against free radical damage, thrombolytic drugs, antiplatelet aggregation drugs, anti-inflammatory drugs, and anticoagulants [4–6]. These drugs have serious side effects and a single mechanism of action, limiting their clinical use to a certain extent [7–10].

Traditional Chinese medicine compounds, which act through multiple components, pathways, and targets, have shown good clinical effects and thus are increasingly used to treat IS. This study provides new strategies for the treatment of IS in China. *Panax notoginseng*, as a traditional Chinese medicine, removes blood stasis, stops bleeding, promotes blood circulation, and relieves pain. The main active ingredients of *Panax notoginseng* are *Panax notoginseng* saponins (PNS), including notoginsenoside R1, ginsenoside Rg1, ginsenoside Re, ginsenoside Rb1, and ginsenoside Rd-qt [11, 12]. Studies have shown that PNS alleviate energy metabolism disturbances, balance ion metabolism, reduce the production of free radicals, and accelerate free radical scavenging. PNS exert antiapoptosis effects by maintaining mitochondrial homeostasis, inhibiting inflammation, protecting the blood-brain barrier (BBB), and increasing the blood supply to brain tissue [13]. However, the mechanism underlying the effect of PNS against IS is not completely clear.

Network pharmacology is a method of predicting drug targets and pharmacological mechanisms [14]. By predicting the complicated “drug-target-disease” relationship, it can assist the clinical evaluation of drug safety and effectiveness, reduce the cost of preclinical drug screening, and promote the development of new drugs. Additionally, multicomponent, multitarget, and regulatory networks based on network pharmacology can reveal the clinically complex mechanisms of action of drugs and are especially suitable for studying the mechanisms of Chinese herbal compounds and their complex components from the “holistic view” [15, 16]. Previously, many scholars have demonstrated the predictive value of network pharmacology in in vivo and vitro experiments. Niu et al. [17] predicted that the ability of *Angelica* to treat acute myocardial infarction (AMI) might be related to the AMPK signalling pathway. This prediction was verified by in vitro and in vivo experiments. In vivo and in vitro verification experiments have indicated that Taohe Chengqi decoction may regulate the PI3K/AKT/mTOR and HIF-1*α*/VEGF signalling pathways, consistent with the predicted results of network pharmacology [18]. Fu et al. [19] predicted that IGF1R is the main target of baicalin against epilepsy and verified through in vivo and in vitro experiments that baicalin inhibits IGF1R, thereby alleviating the symptoms of epilepsy. Dong et al. [20] predicted that VEGFA and BCL2A1 are the key targets of *Panax notoginseng* against cardiovascular diseases and verified this prediction by in vitro experiments. Zhang et al. [21] predicted that the PPAR signalling pathway is the key target pathway of Shen-Hong-Tong-Luo (SHTL) against atherosclerosis (AS) and verified that SHTL activates the PPAR pathway and reduces the inflammatory response and lipid accumulation in macrophages, thus reducing the symptoms of AS. Wen et al. [22] predicted that Chaiqin Chengqi decoction (CQCQD) may inhibit the TLR4/NLRP3 pathway, relieve inflammation, and reduce damage in acute pancreatitis (AP). The prediction results were verified by in vivo and in vitro experiments.

**Figure 1:**
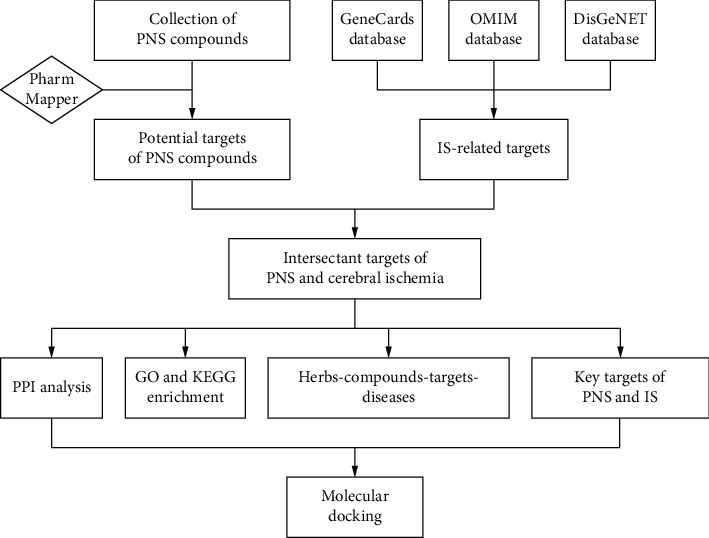
Flowchart of designed analysis in PNS against IS.

We explored the mechanism of PNS against IS through network pharmacology and provided a theoretical basis for the experimental research on the effect of PNS against IS and the efficacy of PNS in the clinical treatment of IS. The research process is shown in Figure 1.

## 2. Materials and Methods

### 2.1. Prediction of the Targets of PNS

The main PNS were identified from the Traditional Chinese Medicine Systems Pharmacology (TCMSP) database and analysis platform [23] (http://tcmspw.com/tcmsp.php), Integrative Pharmacology-Based Research Platform of Traditional Chinese Medicine [24] (TCMIP, http://www.tcmip.cn/), and Bioinformatics Analysis Tool for Molecular Mechanism of Traditional Chinese Medicine [25] (BATMAN- TCM, http://bionet.ncpsb.org/batman-tcm/). The gene targets of the active compounds were downloaded from the PharmMapper database [26] (http://www.lilab-ecust.cn/pharmmapper/).

### 2.2. Prediction of IS-Related Targets

IS-related targets were identified from the following three disease-related databases: the GeneCards database (https:/www.genecards.org/), OMIM database (https://www.omim.org/), and DisGeNET database [27] (https://www.disgenet.org/). We used the keywords “brain infarction,” “cerebral ischaemia,” “cerebral infarction,” and “stroke” to search these databases for IS-related genes, merged the data from the databases, and deleted the duplicate data.

### 2.3. Construction of the Protein-Protein Interaction (PPI) Network

The PNS targets and IS-related targets that intersected were identified as common targets. The common PNS and IS-related targets were uploaded to the STRING 11.0 database (https://string-db.org/) [28], the type was set to “*Homo sapiens*,” the confidence level was set to > 0.700, the “hide disconnected nodes in the network” setting was simplified, the protein interaction information was obtained, the file was saved in the TSV format, and the data for node1, node2, and the combined score were imported into Cytoscape 3.7.2 [29] to establish a PPI network.

### 2.4. Establishment of an “Herb-Compound-Target-Disease” Network

The active compounds, common targets, PNS, and IS were arranged in a relationship pair file and then imported into Cytoscape 3.7.2 to construct a visual “herb-compound-target-disease” network.

### 2.5. Gene Set Enrichment Analysis

To further determine the mechanism of PNS against IS, the common PNS and IS targets were uploaded to the DAVID 6.8 database [30] (https://david.ncifcrf.gov/), the select identifier was limited to “OFFICIAL GENE SYMBOL,” the list type was limited to “Gene List,” and the species was limited to “*Homo sapiens*.” Gene Ontology (GO) and KEGG pathway enrichment analysis of the common targets were performed with this database.

### 2.6. Molecular Docking Studies

Preprocessing structures were generated using AutoDock 4.2 (http://autodock.scripps.edu/). Docking simulations were performed using AutoDock Vina [31]. The crystal structures of the target proteins were downloaded from the Protein Data Bank (http://www.rcsb.org) [32]. The 2D structures of the natural ligands of the PNS compounds were downloaded from PubChem (https://pubchem.ncbi.nlm.nih.gov/).

### 2.7. Middle Cerebral Artery Occlusion (MCAO) Establishment

Adult male Sprague-Dawley rats (260 ± 20 g bodyweight) were purchased from Huaxing Experimental Animal Farm in Huiji District, Zhengzhou City (Zhengzhou, Henan, China). All the animals were housed in an environment with a temperature of 23 ± 2°C, relative humidity of 55 ± 5%, and a light/dark cycle of 12/12 hr with free access to food and water. In addition, all animal studies (including the mice euthanasia procedure) were performed in compliance with the regulations and guidelines of Anhui Science and Technology University Institutional Animal Care and conducted according to the AAALAC and the IACUC guidelines. Rats were randomly divided into six groups as follows: (1) Sham group received 10 mL/kg 0.5% CMC daily, and the procedure was the same as that of MCAO except for the insertion of a filament; (2) MCAO group received 10 mL/kg 0.5% CMC solution daily before MCAO surgery. (3) PNS-L group received 40 mg/kg PNS (5 mg/pill, KPC Xuesaitong Pharmaceutical Co., Ltd.) daily before MCAO surgery. (4) PNS-H group received 80 mg/kg PNS daily before MCAO surgery; and (5) nimodipine group received 14.4 mg/kg nimodipine (20 mg/pill, Yabao Phamarceutical Group Co., Ltd.) daily before MCAO surgery. All the above rates were given intragastric administration once a day for 7 days.

Before the operation, the rats were fasted for 12 h to reduce the mortality during and after the operation and anesthetized by intraperitoneal injection of 2% pentobarbital sodium (4 mL/kg). According to the previous literature, we slowly inserted the threaded plug from the left external carotid artery (ECA) through the common carotid artery (CCA) into the internal carotid artery (ICA) until the middle cerebral artery (MCA) was blocked. After ischaemia 2 h, the blood supply was restored, and reperfusion was achieved. The reperfusion process continued for 24 h.

### 2.8. Oxidative Stress Evaluation

SOD, GSH-Px, CAT activities, and the content of MDA in the brain homogenate were determined according to manufacturer's instructions. All kits were purchased from Nanjing Jiancheng Bioengineering Institute.

### 2.9. Western Blot

The total proteins in the cerebral ischemia-reperfusion brain were were quantified with a bicinchoninic acid protein assay kit (P0010, Beyotime, Shanghai, China), loaded into 10% sodium dodecyl sulfate-polyacrylamide gel electrophoresis, and transferred to polyvinylidene fluoride (PVDF) membranes. After blocking with 5% fat-free milk or BSA for 1 h, the PVDF membranes were incubated with primary antibodies at 4°C overnight (Table 1), following incubation with second antibody (66009-1-Ig, Proteintech, Wuhan, China) for 2 hours at room temperature. The protein bands were visualized using enhanced chemiluminescence (ECL), *β*-actin served as a loading control, and densitometric analysis was determined by ImageJ software.

### 2.10. Statistical Analysis

All data were expressed as mean ± SD; SPSS 25.0 statistical software (IBM SPSS Statistics for Windows, IBM Corp.) was adopted for all statistical analyses. The multiple variables were performed by one-way analysis of variance (ANOVA), and Student”s *t*-tests were used for comparison of variable pairs. *P* < 0.05 was considered as statistically significant.

## 3. Results

### 3.1. Predicted Targets of PNS

**Figure 2:**
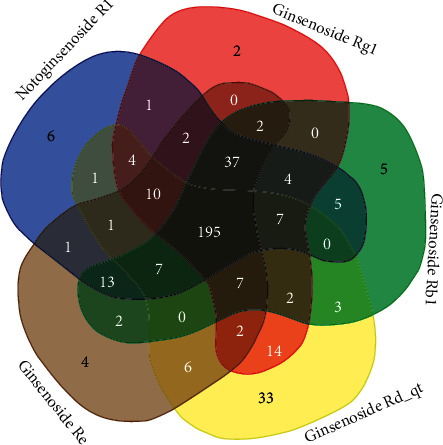
Venn diagram of the gene targets of PNS.

The details of the 5 compounds in PNS are described in Supplementary Table S1. The results retrieved from the PharmMapper databases were integrated to obtain the 300 targets with the highest matching degree with the five active PNS compounds. All targets were uploaded to the UniProt KB database and named uniformly. After deduplication, all information were merged, resulting in a database of 375 targets of PNS, including 196 common targets (Figure 2).

### 3.2. Screening of IS-Related Genes

**Figure 3:**
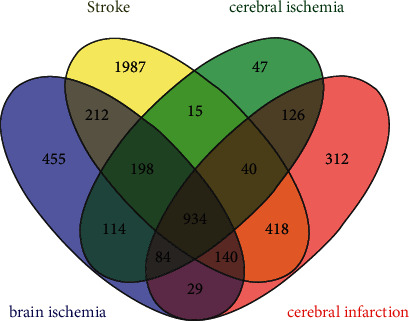
Venn diagram of gene targets related to IS.

Targets related to IS were identified from the GeneCards, OMIM, and DisGeNET databases. The targets were renamed using the UniProt KB database. After deduplication, all information was merged, resulting in a database of 5111 IS-related targets, including 934 common targets (Figure 3).

### 3.3. Construction of the PPI Network

**Figure 4:**
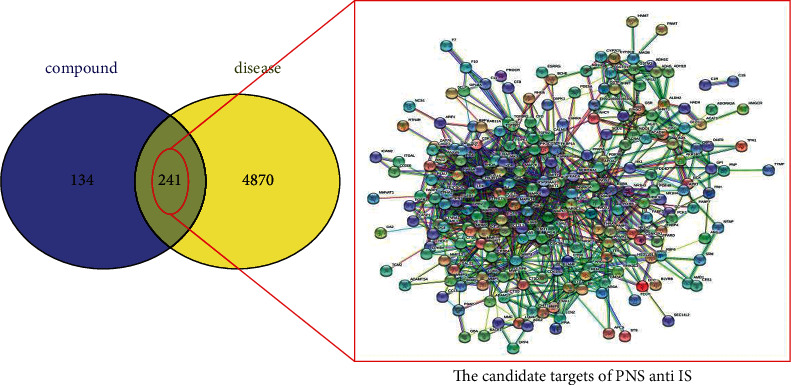
Venn diagram and PPI network of target genes of PNS and IS.

A total of 241 common targets of IS and PNS were identified with a Venn tool. The network constructed with the STRING 11.0 database ignored disconnected targets, resulting in a PPI network of only 224 targets (Figure 4).

**Figure 5:**
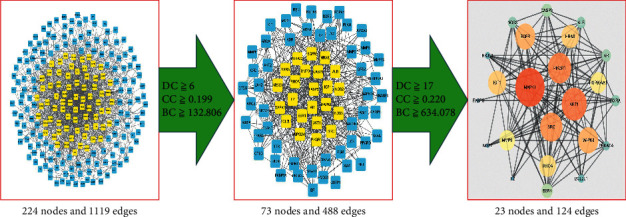
The screening process for the PPI network.

In the PPI network, there were a total of 224 nodes and 1119 edges. The three main parameters “degree (DC),” “closeness,” and “betweenness (BC)” were set as filters to select core targets and construct the main hub of the node representing the effect of PNS against IS. The first screening threshold DC ≥ 6, BC ≥ 132.806, closeness≥0.199, and 73 nodes and 488 edges were identified. The second threshold DC ≥ 17, BC ≥ 634.078, closeness≥0.220, and 23 nodes and 124 edges were identified (Figure 5).

**Figure 6:**
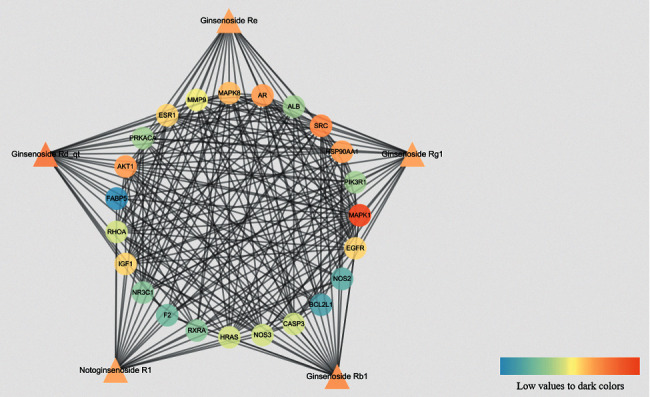
The compound-core target connection network.

Based on these 23 core targets, we further constructed a “compound-core target” network (Figure 6). Ginsenoside Re was related to 19 targets, ginsenoside Rg1 was related to 19 targets, ginsenoside Rb1 was related to 20 targets, ginsenoside Rd_qt was related to 21 targets, and notoginsenoside R1 was related to 19 targets. The colour of each node represents the DC.

The triangular nodes represent compounds, and the circular node represents core targets.

SRC, MAPK1, NOS2, NR3C1, ALB, HRAS, NOS3, CASP3, MAPK8, RHOA, FABP5, HSP90AA1, BCL2L1, PRKACA, PIK3R1, F2, AKT1, IGF1, EGFR, MMP9, AR, RXRA, and ESR1 were arranged according to the DC (Table 2). The top ten targets were MAPK1 (58), AKT1 (50), PIK3R1 (47), SRC (45), MAPK8 (41), EGFR (41), IGF1 (38), HRAS (37), RHOA (35), and HSP90AA1 (34). These targets are the key targets of the PPI network.

### 3.4. Establishment of an “Herb-Compound-Target-Disease” Network

**Figure 7:**
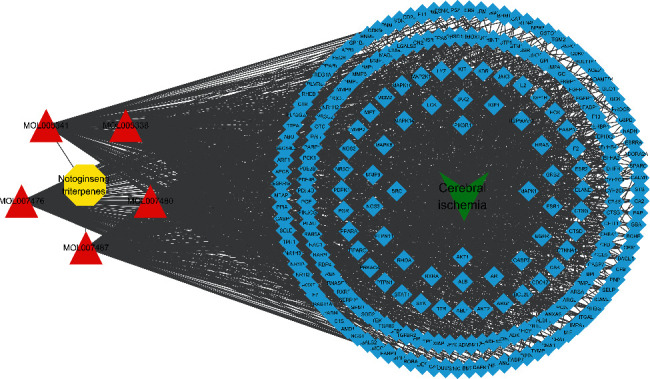
Herb-compound-target-disease network.

The herb-compound-target-disease network was composed of one herb, five compounds, 241 targets, and one disease. A diagram of this network is shown in Figure 7.

The circles represent PNS, the triangles represent PNS compounds, the squares represent the 241 targets, and the V represents IS.

### 3.5. Results of GO Enrichment and KEGG Pathway Analysis

**Figure 8:**
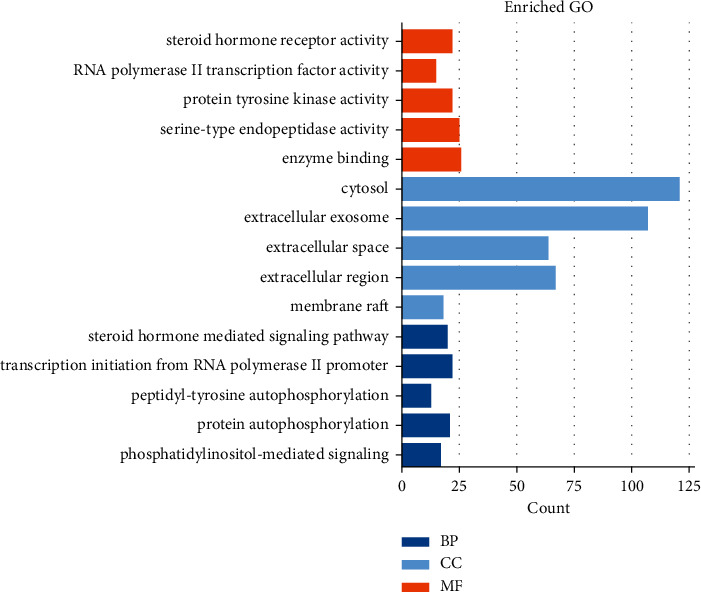
GO enrichment analysis of the 241 nodes (BP, MF, and CC categories).

We performed GO enrichment and KEGG pathway analysis of the common targets. For GO enrichment analysis (Supplementary Tables S2, S3, S4), we filtered the top five cellular components (CC), biological process (BP), and molecular function (MF) categories based on the following criteria: *p* value < 0.01 and FDR < 0.05. The top 5 entries in the BP category were the steroid hormone-mediated signalling pathway, transcription initiation from RNA polymerase II promoter, peptidyl-tyrosine autophosphorylation, protein autophosphorylation, and phosphatidylinositol-mediated signalling. The top 5 entries in the MF category were steroid hormone receptor activity, RNA polymerase II transcription factor activity, ligand-activated sequence-specific DNA binding, protein tyrosine kinase activity, and serine-type endopeptidase activity. The top 5 entries in the CC category were cytosol, extracellular exosome, extracellular space, extracellular region, and membrane raft. The results are shown in Figure 8.

**Figure 9:**
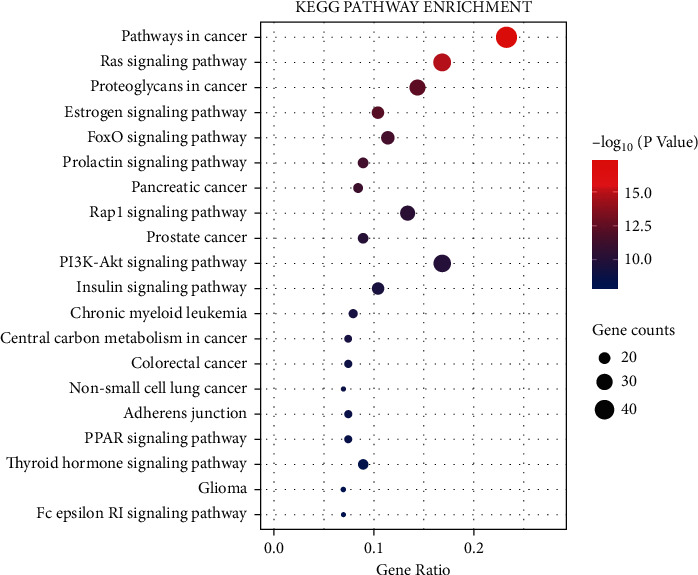
Top 20 enriched KEGG pathways.

To confirm the key pathways enriched in the targets of PNS against IS, we filtered the top 20 KEGG pathways (Supplementary Table S5). The entries were divided into five categories: biochemical substances involved in cancer (proteoglycans and central carbon metabolites), pathways in cancer, human diseases associated with cancer (pancreatic cancer, prostate cancer, colorectal cancer, chronic myeloid leukaemia, and nonsmall-cell lung cancer and glioma), signal transduction pathways (Ras, oestrogen, FoxO, prolactin, Rap1, PI3K-Akt, insulin, PPAR, and thyroid hormone signalling pathways); and types of cell junctions (adherens junctions). Specific information about the top 20 entries is shown in Figure 9.

### 3.6. Results of Molecular Docking Studies

**Figure 10:**
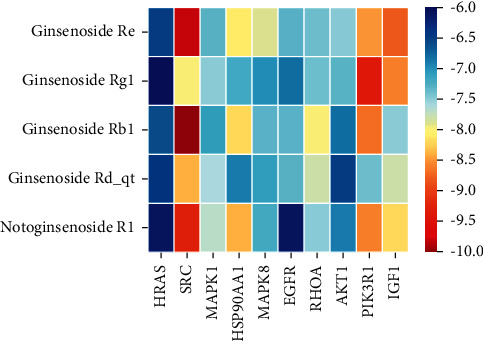
Docking information for 10 targets and their corresponding compounds.

**Figure 11:**
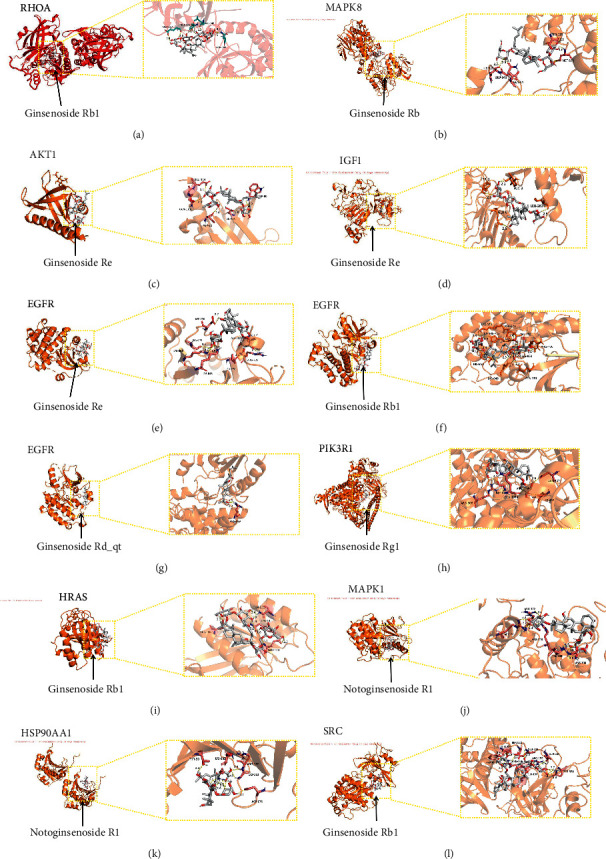
Detailed target compound interactions in the docking simulation. (a) RHOA protein ginsenoside Rb1; (b) MAPK8 protein ginsenoside Re; (c) AKT1 protein ginsenoside Re; (d) IGF1 protein ginsenoside Re; (e) EGFR protein ginsenoside Re; (f) EGFR protein ginsenoside Rb1; (g) EGFR protein ginsenoside Rd_qt; (h) PIK3R1 protein ginsenoside Rg1; (i) HRAS protein ginsenoside Rb1; (j) MAPK1 protein notoginsenoside R1; (k) HSP90AA1 protein notoginsenoside R1; (l) SRC protein ginsenoside Rb1.

We used binding energy to assess the effects of compound-target interactions. Docking of compounds and key targets was simulated, and the results are given in Table 3 and Figure 10. The binding energy of each stimulation was less than −6 kcal/mol, indicating molecular docking was effective. The highest docking scores are shown in Figure 11.

### 3.7. PNS Reduces Oxidative Stress in MCAO Rats

**Figure 12:**
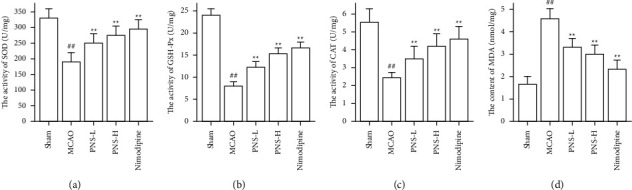
Effects of PNS on MCAO inducing oxidative stress. (a) SOD activity; (b) GSH-Px activity; (c) CAT activity; (d) MDA level. Results were showed as mean ± SD (*n* = 5; *n*, numbers of rats), ^#^*P* < 0.05, ^##^*p* < 0.01 vs. the sham group. ^*∗*^*P* < 0.05, ^*∗∗*^*p* < 0.01 vs. the MCAO group.

The activity of SOD, GSH-Px, and CAT in the brain tissue was higher in the MCAO group than in the sham group (Figures 12(a)–12(c), ^##^*p* < 0.01), and the concentration of MDA was lower in the MCAO group than in the sham group (Figure 12(d), ^##^*p* < 0.01).

The activity of SOD, GSH-Px, and CAT in the brain tissue of the PNS group was much greater than in the MCAO group (Figures 12(a)–12(c), ^*∗∗*^*p* < 0.01). The concentration of MDA in the brain tissue of the PNS group was significantly lower compared to that in the MCAO group (Figure 12(d), ^*∗∗*^*p* < 0.01).

### 3.8. The Protein Expression of the IGF/PI3K/AKT Pathway Mediated by PNS

**Figure 13:**
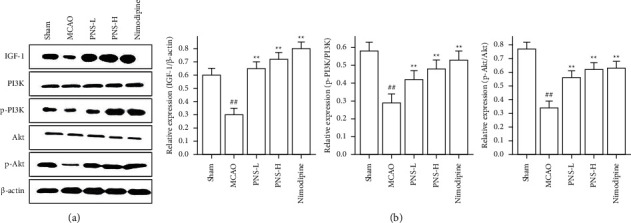
The effects of PNS on the IGF/PI3K/Akt pathway. (a) The IGF1, Akt, mTOR, p-Akt, and p-mTOR expression were measured using Western blotting. (b) The data of IGF1/*β*-actin, p-Akt/Akt, and p-mTOR/mTOR expression. Results were shown as mean ± SD (*n* = 5, *n*: numbers of rats), ^#^*P* < 0.05, ^##^*p* < 0.01 vs. the sham group. ^*∗*^*P* < 0.05, ^*∗∗*^*P* < 0.01 vs. the MCAO group.

As the top 20 of the KEGG pathway, the PI3K-Akt signalling pathway played an important role, and IGF was its upstream protein. Therefore, we focused on the regulation of PNS on the protein of IGF and its downstream PI3K-Akt signal pathway. The IGF1/*β*-actin, p-Akt/Akt, and p-mTOR/mTOR expression in the MCAO control rats were significantly lower than in the sham rats (Figure 13(b), ^##^*p* < 0.01). The IGF1/*β*-actin, p-Akt/Akt, and p-mTOR/mTOR expression in the PNS treatment rats were significantly higher than in the MCAO control rats (Figure 13(b), ^*∗∗*^*p* < 0.01). There was no significant change in total PI3K and Akt protein expression in each group (*p* > 0.05).

## 4. Discussion

IS is a clinically acute disease. Within the first few minutes to a few hours after intracerebral artery occlusion, the infarcted area containing ischaemic tissue gradually spreads outward. As time passes after reperfusion, the ability of brain tissue to recovery decreases gradually [33]. Arterial occlusion is thought to cause the apoptosis of 2 million neurons per minute, and the number of neurons that becomes apoptotic within 10 hours is equivalent to the number of neurons that becomes apoptotic over 36 years of normal ageing [34]. The current treatment plan addresses the changes observed in animal models of local cerebral ischaemia and reperfusion. The primary goal is to relieve arterial obstruction as soon as possible, achieve recanalization, restore cerebral blood flow, and achieve reperfusion to reduce tissue damage and relieve symptoms [33]. Reteplase [8], a commonly used thrombolytic drug, has side effects, such as visceral bleeding. Dexamethasone [9, 10], a commonly used anti-inflammatory drug, has side effects, such as inhibition of the body's immune function and induction of glucose metabolism disorders and osteoporosis. Studies have reported that PNS or their active compounds can reduce ROS production, relieve inflammation, inhibit cell apoptosis, and protect neurons. PNS have not been reported to have any side effects. Notoginsenoside R1 [35] reduces cerebral hypoxic-ischaemic damage in neonatal rats. Ginsenoside Rg1 [36] has a neuroprotective effect against cerebral ischemia-reperfusion injury. In particular, pretreatment with ginsenoside Rd-qt alone alleviates early MCAO-induced damage in rats. Moreover, administration of ginsenoside Rd-qt to rats within 4 hours after MCAO injury has an apparent neuroprotective effect. The therapeutic window of thrombolytic drugs is 6–12 hours after onset [37, 38]. In comparison, ginsenoside Rd-qt can improve the prognosis of patients 72 hours after IS onset [39].

Most Chinese medicines regulate the expression of pathways by acting on multiple genes or proteins and usually do not have serious side effects. For this reason, it is impossible to confirm how traditional Chinese medicines affect protein expression in organisms. However, network pharmacology can determine the potential molecular mechanism of drugs with multiple targets. Network pharmacology mainly involves prediction of compound and disease targets, construction of networks, PPI analysis, GO and KEGG enrichment analysis, and molecular docking studies and was used here to evaluate the mechanism underlying the therapeutic effect of PNS in IS.

In this study, it was confirmed that PNS and IS have 224 common gene targets; a PPI network was constructed; DC, BC, and closeness were determined for two screenings; and 23 targets were identified. The targets with the top ten DCs were MAPK1, AKT1, PIK3R1, SRC, MAPK8, EGFR, IGF1, HRAS, RHOA, and HSP90AA1, which are the key targets.

According to GO analysis, the targets of PNS against IS regulate BP terms, affecting CC and MF terms. The results showed that PNS mainly regulate the steroid hormone-mediated signalling pathway, RNA polymerase II promoter transcription, autophosphorylation of peptidyl-tyrosine, autophosphorylation of proteins, and phosphatidylinositol-mediated signalling. It was also shown that PNS affects specific CC and MF terms, including cytosol, extracellular region, steroid hormone receptor activity, RNA polymerase II transcription factor activity, protein tyrosine kinase activity, and serine endopeptidase activity.

KEGG enrichment analysis indicated that that the targets were mainly enriched in signalling pathways, cell connection types, cancer-related diseases, biochemical substances, and pathways. According to reports in the literature, inhibition of the expression of related proteins in the Ras-MEK-ERK pathway can inhibit vascular intimal hyperplasia after stroke and reduce neuronal damage [40–42]. Activation of the PPAR*γ*-Nrf2-NF-*κ*B signalling pathway can reduce neuroinflammation after stroke [43]. Activation of the PI3K-Akt-FOXO pathway inhibits the transcription of FOXO-3a and promotes the proliferation of neuronal stem cells [44]. The prolactin, oestrogen, and Rap1 signalling pathways can also activate the PI3K-Akt signalling pathway and inhibit cell apoptosis [45–47]. Therefore, it can be predicted that PNS mainly exerts its effect against IS through PI3K- and Akt-related proteins. Akt usually acts as a critical regulator of cell growth, apoptosis, and proliferation, while PI3K mainly activates Akt. The PI3K-Akt signalling pathway has a neuroprotective effect in MCAO rats through inhibition of cell apoptosis [48, 49]. By activating the PI3K-Akt signalling pathway, Danhong injection increases the expression of antiapoptotic factors, inhibits the expression of apoptotic factors, and exerts a neuroprotective effect in MCAO rats [50]. Electroacupuncture reduces the cerebral infarct volume and the activation of cleaved caspase-3 in MCAO rats by activating the PI3K-Akt signalling pathway [51]. Ligustilide-induced activation of the PI3K-Akt signalling pathway inhibits hippocampal neuron apoptosis in two models of oxygen-glucose deprivation/reperfusion (OGD/R) and in MCAO rats [52]. SOX5 promotes the expression of VEGF, which activates the PI3K-Akt signalling pathway and protects nerves in MCAO rats [53]. PNS activate the Nrf2 antioxidant signal through the PI3K/Akt signalling pathway to prevent BBB damage induced by OGD/R [54]. Maintaining BBB integrity is a critical strategy for the prevention of IS [55].

Notoginsenoside Rb1 regulates the Akt-mTOR-PTEN signalling pathway and can alleviate motor and cognitive impairment induced by MCAO in rats [56]. PNS may improve the survival of endothelial cells in MCAO mice through the PI3K-Akt signalling pathway and reduce the cytotoxicity of leukocytes [57].

The results of the network pharmacology study were also confirmed by molecular docking studies. Generally, a binding free energy lower than −5.0 kcal/mol indicates that the docking molecule has excellent binding activity with the target; a binding energy lower than −7.0 kcal/mol indicates that the binding force is strong and that there is a significant interaction. The binding energies of the compounds and core targets were less than −6.0 kcal/mol, indicating good docking and high binding activity; in particular, the binding energy of SRC and ginsenoside Rb1 was −10.0 kcal/mol. Ginsenoside Rb1 protects the endothelial barrier function through the suppression of Src activation [58]. From the perspective of molecular interactions, the results also verified the network pharmacology data.

IGF1, acts as a ligand for IGF1R, activated the PI3K-AKT [59] and Ras-MAPK [6] pathways. PIK3R1 and AKT1 were the key proteins of the PI3K-AKT [59] pathways which regulate apoptosis and oxidative stress. EGFR activated epidermal growth factor, initiated a cascade of downstream signalling events leading to activation of the RAS-RAF-MEK-ERK [60] and PI3K-AKT [61] pathways, and improved the condition of cerebral infarction. MAPK1 and MAPK8 [62] which act as the essential components of the MAP kinase signal transduction pathway involved in cell proliferation, differentiation, migration, and programmed cell death, that was a major downstream signalling pathway of the epidermal growth factor. HSP90AA1 [63] is a molecular chaperone that is involved for instance in the signal transduction and interaction with cochaperone proteins. HRAS [64] was involved in the activation of Ras protein signal transduction and decreased cell apoptosis. Most of the key gene targets are related to the PI3K-AKT pathway. Therefore, the PI3K/Akt pathway plays an important role in PNS anti-IS.

In our study, we found that PNS upregulated the activities of SOD, GSH-Px, and CAT after IS 24 h, whereas PNS downregulated the MDA level, and PNS increased the ratio of IGF1/*β*-actin, p-Akt/Akt, and p-mTOR/mTOR after IS 24 h. These results showed PNS anti-IS through the PI3K-AKT pathway by MCAO rats.

## 5. Conclusions

In this study, the targets, mechanism of action, and associated signalling pathways of PNS in the treatment of IS were predicted through network pharmacology; the binding abilities of the active ingredients of PNS and their targets were verified; and a fundamental basis for elucidating the mechanism of action of PNS was provided. Ginsenoside Rb1 and ginsenoside Rd-qt were related to most core targets. The key targets in the PPI network of the effect of PNS against IS were MAPK1, AKT1, PIK3R1, SRC, MAPK8, EGFR, IGF1, HRAS, RHOA, and HSP90AA1. According to molecular docking analysis, ginsenoside Re and ginsenoside Rb1, the active components of PNS, bind well with the key targets SRC, MAPK1, AKT1, and PIK3R1. Ginsenoside Re and ginsenoside Rb1 may play an essential role in IS treatment. The IGF1/PI3K-Akt signalling pathway, as a strongly associated pathway verified by in vivo experiments, may be used as a critical pathway underlying the effect of PNS against IS. Network pharmacology helps to reveal the mechanisms through which Chinese herbs can treat diseases.

## Figures and Tables

**Table 1:** Primary antibodies for Western blot in this study.

Primary antibodies	Company	Catalog
Anti-AKT	Proteintech	10176-2-AP
Anti-PI3K	Proteintech	67071-1-Ig
Antiphospho-AKT (Ser473)	Proteintech	66444-1-Ig
Antiphospho-PI3K	Proteintech	66444-1-Ig
Anti-IGF	Proteintech	28530-1-AP
Anti-*β*-actin	Proteintech	66009-1-Ig

**Table 2:** Degree information for core targets.

Gene names	Protein names	Degree	Length
SRC	Protooncogene tyrosine-protein kinase Src	58	360
MAPK1	Mitogen-activated protein kinase 1	50	480
NOS2	Nitric oxide synthase type II	47	724
NR3C1	Nuclear receptor subfamily 3 group C member 1	45	536
ALB	Albumin	41	427
HRAS	GTPase HRas	41	1210
NOS3	Nitric oxide synthase type III	38	195
CASP3	Caspase-3	37	189
MAPK8	Mitogen-activated protein kinase 8	35	193
RHOA	Transforming protein RhoA	34	732
FABP5	Fatty acid-binding protein 5	33	707
HSP90AA1	Heat shock protein HSP 90-alpha	27	595
BCL2L1	Bcl-2-like protein 1	26	277
PRKACA	Protein kinase C alpha type	25	920
PIK3R1	Phosphatidylinositol 3-kinase regulatory subunit alpha	24	609
F2	Coagulation factor II	24	1203
AKT1	AKT serine/threonine kinase 1	24	462
IGF1	Insulin-like growth factor I	22	672
EGFR	Epidermal growth factor receptor	21	233
MMP9	Matrix metalloproteinase-9	20	777
AR	Androgen receptor	17	1153
RXRA	Retinoic acid receptor RXR-alpha	17	135
ESR1	Estrogen receptor alpha	17	622

**Table 3:** Docking information for 10 targets and their corresponding compounds.

CAS	Molecule name	Docking score (kcal/mol)									
		HRAS	SRC	MAPK1	HSP90AA1	MAPK8	EGFR	RHOA	AKT1	PIK3R1	IGF1
51542-56-4	Ginsenoside Re	−6.5	−9.8	−7.3	−8.1	−7.9	−7.3	−7.4	−7.5	−8.5	−8.8
22427-39-0	Ginsenoside Rg1	−5.6	−8	−7.5	−7.2	−7.0	−6.8	−7.4	−7.3	−9.4	−8.6
41753-43-9	Ginsenoside Rb1	−6.6	−10	−7.1	−8.2	−7.3	−7.3	−8	−6.8	−8.7	−7.5
62025-49-4	Ginsenoside Rd_qt	−6.4	−8.4	−7.6	−6.9	−7.1	−7.3	−7.8	−6.5	−7.4	−7.8
80418-24-2	Notoginsenoside R1	−6.1	−9.3	−7.7	−8.4	−7.2	−6.1	−7.5	−6.9	−8.6	−8.2

## Data Availability

The data used to support the findings of this study are available from the corresponding author upon request.

## Supplementary Materials

Supplementary Materials The supplementary materials are available online. Table S1: basic information of ingredients in PNS; Table S2: functions of potential target genes based on the GO molecular function; Table S3: functions of potential target genes based on the GO biological process; Table S4: functions of potential target genes based on the GO cellular component; Table S5: functions of potential target genes based on KEGG analysis.
