# Supplementary material for: A Network Pharmacology Approach for Exploring the Mechanisms of Panax notoginseng Saponins in Ischaemic Stroke
Source: Evid Based Complement Alternat Med. 2021 Aug 13;2021:5582782. doi: 10.1155/2021/5582782 (PMC8382556; doi:10.1155/2021/5582782)
Supplement: Supplementary Materials — The supplementary materials are available online. Table S1: basic information of ingredients in PNS; Table S2: functions of potential target genes based on the GO molecular function; Table S3: functions of potential target genes based on the GO biological process; Table S4: functions of potential target genes based on the GO cellular component; Table S5: functions of potential target genes based on KEGG analysis. [file 5582782.f1.zip › 5582782.f1/Supplementary Table S1 Basic information of ingredients in PNS.docx]

Supplementary Table 1 Basic information of ingredients in PNS

| [Mol ID](https://tcmspw.com/tcmspsearch.php?qr=Panax Notoginseng (Burk.) F. H. Chen Ex C. Chow&qsr=herb_en_name&token=c3605f6181fae1557d6818438647a463) | [Molecule Name](https://tcmspw.com/tcmspsearch.php?qr=Chrysanthemi Flos&qsr=herb_en_name&token=00847f58acf9ed0c5f6dec1cbc5c060e) | Chinese Name | [OB (%)](https://tcmspw.com/tcmspsearch.php?qr=Panax Notoginseng (Burk.) F. H. Chen Ex C. Chow&qsr=herb_en_name&token=c3605f6181fae1557d6818438647a463) | [BBB](https://tcmspw.com/tcmspsearch.php?qr=Panax Notoginseng (Burk.) F. H. Chen Ex C. Chow&qsr=herb_en_name&token=c3605f6181fae1557d6818438647a463) | [DL](https://tcmspw.com/tcmspsearch.php?qr=Panax Notoginseng (Burk.) F. H. Chen Ex C. Chow&qsr=herb_en_name&token=c3605f6181fae1557d6818438647a463) |
| --- | --- | --- | --- | --- | --- |
| MOL005338 | Ginsenoside Re | 人参皂苷Re | 4.27 | -4.39 | 0.12 |
| MOL005341 | Ginsenoside Rg1 | 人参皂苷Rg1 | 10.04 | -3.5 | 0.28 |
| MOL007476 | Ginsenoside Rb1 | 人参皂苷Rb1 | 6.29 | -4.95 | 0.04 |
| MOL007480 | Ginsenoside Rd_qt | 人参皂苷Rd_qt | 12.23 | -0.01 | 0.77 |
| MOL007487 | Notoginsenoside R1 | 三七皂苷R1 | 5.43 | -4.15 | 0.13 |
