# Supplementary material for: A Network Pharmacology Approach for Exploring the Mechanisms of Panax notoginseng Saponins in Ischaemic Stroke
Source: Evid Based Complement Alternat Med. 2021 Aug 13;2021:5582782. doi: 10.1155/2021/5582782 (PMC8382556; doi:10.1155/2021/5582782)
Supplement: Supplementary Materials — The supplementary materials are available online. Table S1: basic information of ingredients in PNS; Table S2: functions of potential target genes based on the GO molecular function; Table S3: functions of potential target genes based on the GO biological process; Table S4: functions of potential target genes based on the GO cellular component; Table S5: functions of potential target genes based on KEGG analysis. [file 5582782.f1.zip › 5582782.f1/Supplementary Table S2 Functions of potential target genes based on GO molecular function.docx]

Supplementary Table 2 Functions of potential target genes based on GO molecular function

| Category | Term | Count | Percent | PValue | Genes | FDR |
| --- | --- | --- | --- | --- | --- | --- |
| GOTERM_MF_DIRECT | GO:0003707~steroid hormone receptor activity | 22 | 0.06 | 5.67E-25 | ESRRA, THRA, VDR, NR1H2, NR1I3, NR1I2, NR1H4, NR1H3, RORA, ESRRG, NR3C1, ESR1, ESR2, NR3C2, RXRB, AR, RXRA, RARB, PGR, PPARG, PPARA, PPARD | 3.18E-22 |
| GOTERM_MF_DIRECT | GO:0004879~RNA polymerase II transcription factor activity, ligand-activated sequence-specific DNA binding | 15 | 0.04 | 2.66E-17 | ESRRA, NR1H2, NR1I3, NR1I2, NR1H4, NR1H3, RORA, ESR1, ESR2, RXRB, AR, RXRA, PPARG, PPARA, PPARD | 7.45E-15 |
| GOTERM_MF_DIRECT | GO:0004713~protein tyrosine kinase activity | 22 | 0.06 | 2.26E-16 | MAP2K1, HSP90AA1, SYK, SRC, INSR, EGFR, IGF1R, HCK, ZAP70, ERBB4, LCK, KIT, KDR, ABL1, CSK, TEK, JAK2, JAK3, MET, FGFR2, EPHA2, FGFR1 | 4.22E-14 |
| GOTERM_MF_DIRECT | GO:0004252~serine-type endopeptidase activity | 25 | 0.07 | 2.46E-13 | CFD, C1S, C1R, CTSS, DPP4, PLAU, CTSG, CTSD, ELANE, CTSB, MMP7, F10, MMP1, CMA1, MMP2, MMP3, F11, MMP8, F2, MMP9, MMP12, F7, MMP13, FAP, CFB | 3.45E-11 |
| GOTERM_MF_DIRECT | GO:0019899~enzyme binding | 26 | 0.07 | 1.19E-11 | SRC, EGFR, ACAT1, MAPK8, RXRA, CBS, AKT1, HMOX1, PTPN1, BCHE, HSPA8, GSTM1, PARP1, STAT1, PDE4D, MAPK14, ESR1, ESR2, BACE1, FKBP1A, AR, ADORA2A, MDM2, PPARG, PGR, HSPA1A | 1.33E-09 |
| GOTERM_MF_DIRECT | GO:0005496~steroid binding | 10 | 0.03 | 7.66E-11 | ESRRA, AR, SULT1E1, ESRRG, PGR, NR3C1, SHBG, ESR1, ESR2, NR3C2 | 7.16E-09 |
| GOTERM_MF_DIRECT | GO:0004714~transmembrane receptor protein tyrosine kinase activity | 11 | 0.03 | 9.33E-11 | ERBB4, INSR, KIT, KDR, TEK, MET, EPHB4, EGFR, FGFR2, EPHA2, IGF1R | 7.48E-09 |
| GOTERM_MF_DIRECT | GO:0042802~identical protein binding | 37 | 0.1 | 1.32E-10 | SERPINA1, AHCY, C1S, SHMT1, XIAP, EGFR, SRM, IGF1R, DPP4, CDC42, TTR, IMPA1, CBS, AKT1, MAPK1, CSK, ACADM, NQO1, BCHE, G6PD, HSP90AA1, PARP1, DAPK1, STAT1, LYZ, SOD2, MMP9, ESR1, ADORA2A, NMNAT1, LCK, ALB, MDM2, GRB2, PPARG, FGFR1, BCL2L1 | 9.29E-09 |
| GOTERM_MF_DIRECT | GO:0008270~zinc ion binding | 46 | 0.13 | 7.25E-10 | THRA, ADH1C, ADH1B, NR1I3, GLO1, NR1I2, XIAP, RORA, NR3C1, ADH5, NR3C2, ADAMTS4, RXRB, CA1, RXRA, CA2, LTA4H, ESRRA, PTPN1, MMP7, PARP1, MME, MMP1, VDR, NR1H2, MMP2, MMP3, NR1H4, NR1H3, ESRRG, MMP8, MMP9, ESR1, ESR2, MMP12, ACE2, AR, BHMT, MMP13, MDM2, RARB, PPARG, PGR, PPARA, S100A9, PPARD | 4.52E-08 |
| GOTERM_MF_DIRECT | GO:0005102~receptor binding | 23 | 0.06 | 7.06E-09 | TGFB2, NOS2, SRC, EPHX2, GBA, MIF, F2, DPP4, F7, AR, HCK, ZAP70, BMP2, ABL1, CSK, REN, PGR, ANG, JAK2, AGXT, JAK3, CRAT, HSPA1A | 3.96E-07 |
| GOTERM_MF_DIRECT | GO:0016301~kinase activity | 19 | 0.05 | 1.10E-08 | GSK3B, CSNK2A1, DAPK1, PDPK1, SRC, PIK3R1, PIK3CG, MAPK8, PLAU, LCK, CHEK1, AKT1, MAPK1, CSK, CALM1, JAK2, FGFR2, EPHA2, CDK5R1 | 5.59E-07 |
| GOTERM_MF_DIRECT | GO:0004175~endopeptidase activity | 10 | 0.03 | 6.23E-08 | MMP12, ACE2, MME, FAP, MMP1, CMA1, MMP3, CASP1, MMP9, ELANE | 2.91E-06 |
| GOTERM_MF_DIRECT | GO:0005515~protein binding | 166 | 0.45 | 8.77E-08 | SPARC, RORA, NR3C1, IGF1R, NR3C2, LGALS3, LGALS2, PLAU, AKT2, CHEK1, KDR, AKT1, PRKACA, EPHB4, TGM2, ARSA, MAP2K1, G6PD, CSNK2A1, DAPK1, MIF, PGF, PROCR, AR, ACE2, RBP4, MTAP, ADAM17, PGR, PADI4, PPIA, S100A9, EPHA2, RTN4R, FECH, SHMT1, PIK3R1, DPP4, TTPA, PSAP, ABL1, HMOX1, LTA4H, ELANE, ESRRA, HSPA8, TGFB2, VDR, INSR, ESRRG, IGF1, SELE, ESR1, BMP7, ESR2, SELP, BMP2, CDK6, CD209, ALB, MDM2, GRB2, CALM1, RAB5A, FGFR2, BCL2L1, FGFR1, GSK3B, ARF1, SERPINA1, THRA, PDE3B, ITGAL, PIK3CG, HK1, ADAMTS4, CASP7, CA1, IMPA1, CA2, CASP3, NCS1, CASP1, CTSG, JAK2, JAK3, HRAS, CTSD, CTSB, HSP90AA1, TPI1, PARP1, SYK, MME, PDPK1, GSTO1, PDE4D, MMP2, ANXA5, MMP3, APOA2, GP1BA, F2, MMP9, RHOA, TGFBR1, TGFBR2, BACE1, F7, ZAP70, HCK, ADORA2A, LCK, KIT, ANG, PPARG, PPARA, AGXT, MET, PPARD, AHCY, C1S, SRC, C1R, GSTP1, GBA, NR1I2, XIAP, HMGCR, EGFR, SRM, RXRB, CDC42, MAPK8, TTR, RXRA, SULT1E1, ERBB4, CBS, CCL5, CTNNA1, MAPK1, CSK, PTPN1, NQO1, F10, NOS2, STAT1, NOS3, NR1H2, F11, NR1H4, NR1H3, PTPN11, MAPK14, RAB11A, GCK, MAPK10, FKBP1A, FABP3, NMNAT1, FABP5, FAP, TEK, CDK5R1, HSPA1A | 3.79E-06 |
| GOTERM_MF_DIRECT | GO:0008144~drug binding | 11 | 0.03 | 1.17E-07 | DHFR, CYP2C9, PNP, GSTP1, PDE4D, ALB, NR1I2, RARB, PPARG, PPARA, PPARD | 4.71E-06 |
| GOTERM_MF_DIRECT | GO:0004716~receptor signaling protein tyrosine kinase activity | 6 | 0.02 | 1.32E-07 | SYK, ERBB4, INSR, KIT, KDR, EGFR | 4.96E-06 |
| GOTERM_MF_DIRECT | GO:0042803~protein homodimerization activity | 31 | 0.08 | 1.76E-07 | MAOB, SHMT1, NPR3, HMGCR, ADH5, SRM, ACAT1, DPP4, IMPA1, ERBB4, CBS, CCL5, HMOX1, TGFB2, G6PD, HSP90AA1, GSTM1, NOS2, STAT1, EPHX2, APOA2, PGF, FKBP1A, FAP, KIT, LCN2, ANG, AGXT, FGFR2, FGFR1, BCL2L1 | 6.16E-06 |
| GOTERM_MF_DIRECT | GO:0046934~phosphatidylinositol-4,5-bisphosphate 3-kinase activity | 10 | 0.03 | 2.15E-07 | ERBB4, LCK, KIT, GRB2, PTPN11, PIK3R1, EGFR, FGFR2, PIK3CG, FGFR1 | 6.62E-06 |
| GOTERM_MF_DIRECT | GO:0004672~protein kinase activity | 21 | 0.06 | 2.19E-07 | GSK3B, MAP2K1, SYK, CSNK2A1, DAPK1, PDPK1, SRC, MAPK14, EGFR, TGFBR1, PIK3CG, MAPK10, AKT2, CCL5, CHEK1, ABL1, AKT1, TEK, JAK2, MET, CDK5R1 | 6.62E-06 |
| GOTERM_MF_DIRECT | GO:0043560~insulin receptor substrate binding | 6 | 0.02 | 2.40E-07 | INSR, GRB2, PTPN11, PIK3R1, JAK2, IGF1R | 6.62E-06 |
| GOTERM_MF_DIRECT | GO:0004715~non-membrane spanning protein tyrosine kinase activity | 9 | 0.02 | 2.43E-07 | HCK, ZAP70, SYK, SRC, LCK, ABL1, CSK, JAK2, JAK3 | 6.62E-06 |
| GOTERM_MF_DIRECT | GO:0019903~protein phosphatase binding | 10 | 0.03 | 2.48E-07 | HSP90AA1, LCK, CSK, GRB2, PPARG, PIK3R1, MAPK14, JAK3, MET, EGFR | 6.62E-06 |
| GOTERM_MF_DIRECT | GO:0004222~metalloendopeptidase activity | 12 | 0.03 | 6.20E-07 | MMP12, ADAMTS4, ADAM17, MMP13, MMP7, MME, FAP, MMP1, MMP2, MMP3, MMP8, MMP9 | 1.58E-05 |
| GOTERM_MF_DIRECT | GO:0005524~ATP binding | 46 | 0.13 | 1.06E-06 | GSK3B, SRC, ADK, EGFR, PIK3CG, IGF1R, HK1, MAPK8, ERBB4, AKT2, CHEK1, KDR, ABL1, AKT1, MAPK1, CSK, JAK2, PRKACA, JAK3, EPHB4, TGM2, HSPA8, MAP2K1, HSP90AA1, SYK, CSNK2A1, DAPK1, PDPK1, INSR, MAPK14, TGFBR1, GCK, TGFBR2, MAPK10, HCK, ZAP70, CDK6, NMNAT1, LCK, KIT, TEK, MET, FGFR2, FGFR1, HSPA1A, EPHA2 | 2.59E-05 |
| GOTERM_MF_DIRECT | GO:0030235~nitric-oxide synthase regulator activity | 5 | 0.01 | 2.67E-06 | HSP90AA1, AKT1, CALM1, ESR1, EGFR | 6.23E-05 |
| GOTERM_MF_DIRECT | GO:0005158~insulin receptor binding | 7 | 0.02 | 4.24E-06 | PTPN1, PDPK1, SRC, PTPN11, IGF1, PIK3R1, IGF1R | 9.52E-05 |
| GOTERM_MF_DIRECT | GO:0005088~Ras guanyl-nucleotide exchange factor activity | 11 | 0.03 | 5.65E-06 | ERBB4, KIT, GRB2, TEK, CALM1, JAK2, JAK3, EGFR, IL2, FGFR2, FGFR1 | 1.22E-04 |
| GOTERM_MF_DIRECT | GO:0043565~sequence-specific DNA binding | 22 | 0.06 | 1.69E-05 | ESRRA, THRA, VDR, NR1H2, NR1I3, NR1I2, NR1H4, NR1H3, RORA, ESRRG, NR3C1, ESR1, ESR2, NR3C2, RXRB, AR, RXRA, RARB, PGR, PPARG, PPARA, PPARD | 3.50E-04 |
| GOTERM_MF_DIRECT | GO:0008233~peptidase activity | 9 | 0.02 | 4.22E-05 | ADAMTS4, BACE1, FAP, CASP3, REN, CTSG, LTA4H, ELANE, CTSB | 8.46E-04 |
| GOTERM_MF_DIRECT | GO:0046965~retinoid X receptor binding | 5 | 0.01 | 4.81E-05 | VDR, NR1H2, NR1H4, RARB, PPARG | 9.30E-04 |
| GOTERM_MF_DIRECT | GO:0019901~protein kinase binding | 17 | 0.05 | 1.00E-04 | PTPN1, GSK3B, MAP2K1, SYK, PARP1, PDPK1, EGFR, CDC42, RHEB, LCK, NCS1, GRB2, CALM1, JAK2, PRKACA, CDK5R1, BCL2L1 | 0.001874611 |
| GOTERM_MF_DIRECT | GO:0050661~NADP binding | 6 | 0.02 | 1.28E-04 | DHFR, G6PD, NOS2, NOS3, GSR, HMGCR | 0.002219541 |
| GOTERM_MF_DIRECT | GO:0043548~phosphatidylinositol 3-kinase binding | 5 | 0.01 | 1.31E-04 | LCK, INSR, PIK3R1, JAK2, IGF1R | 0.002219541 |
| GOTERM_MF_DIRECT | GO:0005504~fatty acid binding | 5 | 0.01 | 1.31E-04 | FABP4, FABP5, ALB, ADH5, PPARD | 0.002219541 |
| GOTERM_MF_DIRECT | GO:0004887~thyroid hormone receptor activity | 4 | 0.01 | 1.51E-04 | THRA, NR1I3, NR1I2, NR1H4 | 0.002452135 |
| GOTERM_MF_DIRECT | GO:0008237~metallopeptidase activity | 8 | 0.02 | 1.53E-04 | ADAMTS4, ACE2, ADAM17, MME, MMP2, MMP3, LTA4H, MMP9 | 0.002452135 |
| GOTERM_MF_DIRECT | GO:0016597~amino acid binding | 5 | 0.01 | 2.38E-04 | TPH1, SHMT1, PAH, AGXT, OTC | 0.003713211 |
| GOTERM_MF_DIRECT | GO:0001948~glycoprotein binding | 7 | 0.02 | 3.18E-04 | SELP, ACE2, F7, HSP90AA1, SERPINA1, LCK, EGFR | 0.004821214 |
| GOTERM_MF_DIRECT | GO:0016303~1-phosphatidylinositol-3-kinase activity | 6 | 0.02 | 3.45E-04 | GRB2, PTPN11, PIK3R1, FGFR2, PIK3CG, FGFR1 | 0.005095291 |
| GOTERM_MF_DIRECT | GO:0046875~ephrin receptor binding | 5 | 0.01 | 4.66E-04 | PTPN1, SRC, GRB2, PIK3CG, CDK5R1 | 0.006699427 |
| GOTERM_MF_DIRECT | GO:0008201~heparin binding | 10 | 0.03 | 4.81E-04 | SELP, MMP7, F11, CTSG, ANG, BMP7, ELANE, FGFR2, PGF, FGFR1 | 0.006748179 |
| GOTERM_MF_DIRECT | GO:0008083~growth factor activity | 10 | 0.03 | 5.24E-04 | GPI, TGFB2, BMP2, REG1A, IGF1, F2, BMP7, IL2, PGF, TYMP | 0.007164474 |
| GOTERM_MF_DIRECT | GO:0002020~protease binding | 8 | 0.02 | 5.95E-04 | ADAMTS4, DPP4, SERPINA1, FAP, CASP3, KIT, LCN2, ELANE | 0.007793426 |
| GOTERM_MF_DIRECT | GO:0016491~oxidoreductase activity | 11 | 0.03 | 5.97E-04 | HSD11B1, CYP2C9, MAOB, ALDH2, ADH1C, GSTO1, ADH1B, NOS3, GSR, AKR1B1, ADH5 | 0.007793426 |
| GOTERM_MF_DIRECT | GO:0005178~integrin binding | 8 | 0.02 | 7.51E-04 | ADAM17, SYK, FAP, SRC, ICAM2, KDR, IGF1, EGFR | 0.009575968 |
| GOTERM_MF_DIRECT | GO:0004707~MAP kinase activity | 4 | 0.01 | 9.20E-04 | MAPK10, MAPK8, MAPK1, MAPK14 | 0.011468647 |
| GOTERM_MF_DIRECT | GO:0004674~protein serine/threonine kinase activity | 15 | 0.04 | 0.001017122 | GSK3B, MAP2K1, SYK, CSNK2A1, DAPK1, PDPK1, MAPK14, TGFBR1, PIK3CG, MAPK8, AKT2, CHEK1, AKT1, MAPK1, PRKACA | 0.012404462 |
| GOTERM_MF_DIRECT | GO:0005159~insulin-like growth factor receptor binding | 4 | 0.01 | 0.00113791 | INSR, REN, IGF1, PIK3R1 | 0.013582289 |
| GOTERM_MF_DIRECT | GO:0004190~aspartic-type endopeptidase activity | 5 | 0.01 | 0.00117926 | BACE1, CASP7, CASP3, REN, CTSD | 0.013782606 |
| GOTERM_MF_DIRECT | GO:0004197~cysteine-type endopeptidase activity | 6 | 0.02 | 0.001734038 | CASP7, CASP3, CASP1, CTSD, CTSS, CTSB | 0.01985297 |
| GOTERM_MF_DIRECT | GO:0043559~insulin binding | 3 | 0.01 | 0.001956804 | INSR, PIK3R1, IGF1R | 0.021194118 |
| GOTERM_MF_DIRECT | GO:0003708~retinoic acid receptor activity | 3 | 0.01 | 0.001956804 | RXRA, RARB, ESRRG | 0.021194118 |
| GOTERM_MF_DIRECT | GO:0043274~phospholipase binding | 4 | 0.01 | 0.001977489 | PDPK1, PTPN11, CALM1, SELE | 0.021194118 |
| GOTERM_MF_DIRECT | GO:0008236~serine-type peptidase activity | 6 | 0.02 | 0.002002296 | CFD, DPP4, F7, FAP, C1R, CMA1 | 0.021194118 |
| GOTERM_MF_DIRECT | GO:0020037~heme binding | 8 | 0.02 | 0.00346572 | CYP2C9, CYP2C8, NOS2, SRC, CBS, NOS3, HMOX1, JAK2 | 0.036004976 |
| GOTERM_MF_DIRECT | GO:0016004~phospholipase activator activity | 3 | 0.01 | 0.004032702 | GM2A, PDPK1, CCL5 | 0.040087271 |
| GOTERM_MF_DIRECT | GO:0051117~ATPase binding | 6 | 0.02 | 0.004046243 | AR, LCK, NR1H2, PDE4D, PGR, ESR1 | 0.040087271 |
| GOTERM_MF_DIRECT | GO:0004114~3',5'-cyclic-nucleotide phosphodiesterase activity | 4 | 0.01 | 0.004073038 | PDE4D, PDE3B, PDE4B, PDE5A | 0.040087271 |
| GOTERM_MF_DIRECT | GO:0019825~oxygen binding | 5 | 0.01 | 0.004402187 | CYP2C9, CYP2C8, CBS, ALB, SOD2 | 0.042579771 |
| GOTERM_MF_DIRECT | GO:0030145~manganese ion binding | 5 | 0.01 | 0.005499043 | IMPA1, ARG1, ABL1, PCK1, SOD2 | 0.052287513 |
| GOTERM_MF_DIRECT | GO:0051721~protein phosphatase 2A binding | 4 | 0.01 | 0.006452151 | PTPN1, STAT1, AKT1, HMGCR | 0.060327615 |
| GOTERM_MF_DIRECT | GO:0005509~calcium ion binding | 20 | 0.05 | 0.006915076 | APCS, ARSA, SPARC, C1S, F10, C1R, MMP1, PLA2G2A, ANXA5, MMP3, F2, MMP8, MMP12, F7, MMP13, NCS1, PADI4, CALM1, S100A9, CDK5R1 | 0.06359603 |
| GOTERM_MF_DIRECT | GO:0005543~phospholipid binding | 6 | 0.02 | 0.007629907 | SEC14L2, F10, ANXA5, PLA2G2A, APOA2, OTC | 0.069038353 |
| GOTERM_MF_DIRECT | GO:0046790~virion binding | 3 | 0.01 | 0.008401738 | APCS, CD209, PPIA | 0.074815475 |
| GOTERM_MF_DIRECT | GO:0005525~GTP binding | 13 | 0.04 | 0.009070794 | ARF1, HSP90AA1, DAPK1, INSR, RHOA, RAB11A, CDC42, RHEB, RAC2, PCK1, HRAS, RAB5A, TGM2 | 0.07941065 |
| GOTERM_MF_DIRECT | GO:0009055~electron carrier activity | 6 | 0.02 | 0.009200877 | MAOB, ALDH2, GSR, AKR1B1, ACADM, ADH5 | 0.07941065 |
| GOTERM_MF_DIRECT | GO:0042277~peptide binding | 5 | 0.01 | 0.009843651 | MME, CMA1, ANG, LTA4H, PPIA | 0.08367103 |
| GOTERM_MF_DIRECT | GO:0005536~glucose binding | 3 | 0.01 | 0.01017315 | G6PD, GCK, HK1 | 0.085181153 |
| GOTERM_MF_DIRECT | GO:0005518~collagen binding | 5 | 0.01 | 0.010431749 | MMP13, SPARC, MMP9, CTSS, CTSB | 0.086061929 |
| GOTERM_MF_DIRECT | GO:0004708~MAP kinase kinase activity | 3 | 0.01 | 0.012094214 | MAPK10, MAP2K1, MAPK14 | 0.098331215 |
| GOTERM_MF_DIRECT | GO:0050660~flavin adenine dinucleotide binding | 5 | 0.01 | 0.01300973 | MAOB, NOS2, NOS3, GSR, ACADM | 0.10426369 |
| GOTERM_MF_DIRECT | GO:0097153~cysteine-type endopeptidase activity involved in apoptotic process | 3 | 0.01 | 0.014160352 | CASP7, CASP3, CASP1 | 0.111886722 |
| GOTERM_MF_DIRECT | GO:0010181~FMN binding | 3 | 0.01 | 0.018710036 | NOS2, NOS3, PNPO | 0.143785346 |
| GOTERM_MF_DIRECT | GO:0004115~3',5'-cyclic-AMP phosphodiesterase activity | 3 | 0.01 | 0.018710036 | PDE4D, PDE3B, PDE4B | 0.143785346 |
| GOTERM_MF_DIRECT | GO:0003924~GTPase activity | 9 | 0.02 | 0.019049524 | CDC42, ARF1, RHEB, RAC2, HRAS, RAB5A, RHOA, RAB11A, TGM2 | 0.144415984 |
| GOTERM_MF_DIRECT | GO:0001077~transcriptional activator activity, RNA polymerase II core promoter proximal region sequence-specific binding | 9 | 0.02 | 0.020070469 | ESRRA, AR, NR1H2, NR1H4, NR1H3, PGR, PPARA, NR3C1, ESR1 | 0.150127108 |
| GOTERM_MF_DIRECT | GO:0008134~transcription factor binding | 10 | 0.03 | 0.020530704 | AR, THRA, PARP1, MAPK1, PPARG, RORA, PIK3R1, PPARA, ESR1, PPARD | 0.151549016 |
| GOTERM_MF_DIRECT | GO:0008289~lipid binding | 7 | 0.02 | 0.021008968 | FABP3, FABP5, FABP7, PSAP, APOA2, PPARA, PPARD | 0.153065336 |
| GOTERM_MF_DIRECT | GO:0005160~transforming growth factor beta receptor binding | 4 | 0.01 | 0.021678757 | FKBP1A, TGFB2, BMP2, BMP7 | 0.154816646 |
| GOTERM_MF_DIRECT | GO:0031625~ubiquitin protein ligase binding | 10 | 0.03 | 0.021801275 | HSPA8, GSK3B, GPI, TPI1, CBS, PDE4D, MDM2, PRKACA, EGFR, HSPA1A | 0.154816646 |
| GOTERM_MF_DIRECT | GO:0044325~ion channel binding | 6 | 0.02 | 0.022659479 | FKBP1A, HSP90AA1, SRC, PDE4D, PDE4B, CALM1 | 0.157804555 |
| GOTERM_MF_DIRECT | GO:0046332~SMAD binding | 4 | 0.01 | 0.023065906 | FKBP1A, BMP2, TGFBR1, TGFBR2 | 0.157804555 |
| GOTERM_MF_DIRECT | GO:0001046~core promoter sequence-specific DNA binding | 4 | 0.01 | 0.023065906 | PPARG, RORA, ESR1, ESR2 | 0.157804555 |
| GOTERM_MF_DIRECT | GO:0031435~mitogen-activated protein kinase kinase kinase binding | 3 | 0.01 | 0.023787535 | CDC42, MAPK1, TGFBR2 | 0.160780806 |
| GOTERM_MF_DIRECT | GO:0005215~transporter activity | 8 | 0.02 | 0.025738346 | SEC14L2, FABP3, RBP4, FABP4, FABP5, TTPA, FABP7, LCN2 | 0.171895385 |
| GOTERM_MF_DIRECT | GO:0043539~protein serine/threonine kinase activator activity | 3 | 0.01 | 0.02651379 | MAP2K1, CALM1, CDK5R1 | 0.174991016 |
| GOTERM_MF_DIRECT | GO:0005080~protein kinase C binding | 4 | 0.01 | 0.027510303 | HINT1, SRC, ABL1, AKT1 | 0.1779633 |
| GOTERM_MF_DIRECT | GO:0004053~arginase activity | 2 | 0.01 | 0.028233037 | ARG2, ARG1 | 0.1779633 |
| GOTERM_MF_DIRECT | GO:0004645~phosphorylase activity | 2 | 0.01 | 0.028233037 | MTAP, TYMP | 0.1779633 |
| GOTERM_MF_DIRECT | GO:0038052~RNA polymerase II transcription factor activity, estrogen-activated sequence-specific DNA binding | 2 | 0.01 | 0.028233037 | ESR1, ESR2 | 0.1779633 |
| GOTERM_MF_DIRECT | GO:0008013~beta-catenin binding | 5 | 0.01 | 0.029438967 | GSK3B, AR, CTNNA1, RORA, ESR1 | 0.183502892 |
| GOTERM_MF_DIRECT | GO:0097110~scaffold protein binding | 4 | 0.01 | 0.030708267 | SRC, NOS3, PDE4D, MDM2 | 0.189311402 |
| GOTERM_MF_DIRECT | GO:0003700~transcription factor activity, sequence-specific DNA binding | 22 | 0.06 | 0.033449293 | ESRRA, THRA, STAT1, VDR, NR1H2, NR1I3, NR1I2, NR1H4, NR1H3, RORA, ESRRG, NR3C1, ESR1, ESR2, NR3C2, RXRB, AR, RXRA, PGR, PPARG, PPARA, PPARD | 0.20396797 |
| GOTERM_MF_DIRECT | GO:0046982~protein heterodimerization activity | 13 | 0.04 | 0.034321424 | TGFB2, APOA2, PIK3R1, ITGAL, EGFR, PGF, BMP2, RBP4, TTR, RXRA, CTNNA1, BCL2L1, PPARD | 0.207035687 |
| GOTERM_MF_DIRECT | GO:0004702~receptor signaling protein serine/threonine kinase activity | 4 | 0.01 | 0.039516921 | TGFB2, MAP2K1, TGFBR1, TGFBR2 | 0.23127746 |
| GOTERM_MF_DIRECT | GO:0004705~JUN kinase activity | 2 | 0.01 | 0.042050447 | MAPK10, MAPK8 | 0.23127746 |
| GOTERM_MF_DIRECT | GO:0097200~cysteine-type endopeptidase activity involved in execution phase of apoptosis | 2 | 0.01 | 0.042050447 | CASP7, CASP3 | 0.23127746 |
| GOTERM_MF_DIRECT | GO:0004882~androgen receptor activity | 2 | 0.01 | 0.042050447 | AR, NR1I3 | 0.23127746 |
| GOTERM_MF_DIRECT | GO:0008431~vitamin E binding | 2 | 0.01 | 0.042050447 | SEC14L2, TTPA | 0.23127746 |
| GOTERM_MF_DIRECT | GO:0004517~nitric-oxide synthase activity | 2 | 0.01 | 0.042050447 | NOS2, NOS3 | 0.23127746 |
| GOTERM_MF_DIRECT | GO:0004886~9-cis retinoic acid receptor activity | 2 | 0.01 | 0.042050447 | RXRB, RXRA | 0.23127746 |
| GOTERM_MF_DIRECT | GO:0070644~vitamin D response element binding | 2 | 0.01 | 0.042050447 | RXRA, VDR | 0.23127746 |
| GOTERM_MF_DIRECT | GO:0070026~nitric oxide binding | 2 | 0.01 | 0.042050447 | CBS, GSTP1 | 0.23127746 |
| GOTERM_MF_DIRECT | GO:0008022~protein C-terminus binding | 7 | 0.02 | 0.046042748 | MAP2K1, SRC, LCK, ABL1, CSK, JAK2, HRAS | 0.250776522 |
| GOTERM_MF_DIRECT | GO:0030170~pyridoxal phosphate binding | 4 | 0.01 | 0.047385131 | CBS, SHMT1, ALB, AGXT | 0.255606333 |
| GOTERM_MF_DIRECT | GO:0004497~monooxygenase activity | 4 | 0.01 | 0.049463729 | CYP2C9, CYP2C8, TPH1, PAH | 0.264277635 |
| GOTERM_MF_DIRECT | GO:0004301~epoxide hydrolase activity | 2 | 0.01 | 0.055672197 | EPHX2, LTA4H | 0.266144027 |
| GOTERM_MF_DIRECT | GO:0016714~oxidoreductase activity, acting on paired donors, with incorporation or reduction of molecular oxygen, reduced pteridine as one donor, and incorporation of one atom of oxygen | 2 | 0.01 | 0.055672197 | TPH1, PAH | 0.266144027 |
| GOTERM_MF_DIRECT | GO:0034056~estrogen response element binding | 2 | 0.01 | 0.055672197 | ESR1, ESR2 | 0.266144027 |
| GOTERM_MF_DIRECT | GO:0034617~tetrahydrobiopterin binding | 2 | 0.01 | 0.055672197 | NOS2, NOS3 | 0.266144027 |
| GOTERM_MF_DIRECT | GO:0034875~caffeine oxidase activity | 2 | 0.01 | 0.055672197 | CYP2C9, CYP2C8 | 0.266144027 |
| GOTERM_MF_DIRECT | GO:0051022~Rho GDP-dissociation inhibitor binding | 2 | 0.01 | 0.055672197 | CDC42, HSP90AA1 | 0.266144027 |
| GOTERM_MF_DIRECT | GO:0043208~glycosphingolipid binding | 2 | 0.01 | 0.055672197 | SELP, IL2 | 0.266144027 |
| GOTERM_MF_DIRECT | GO:0005497~androgen binding | 2 | 0.01 | 0.055672197 | AR, SHBG | 0.266144027 |
| GOTERM_MF_DIRECT | GO:0034191~apolipoprotein A-I receptor binding | 2 | 0.01 | 0.055672197 | CDC42, NR1H2 | 0.266144027 |
| GOTERM_MF_DIRECT | GO:0034714~type III transforming growth factor beta receptor binding | 2 | 0.01 | 0.055672197 | TGFB2, TGFBR2 | 0.266144027 |
| GOTERM_MF_DIRECT | GO:0004515~nicotinate-nucleotide adenylyltransferase activity | 2 | 0.01 | 0.055672197 | NMNAT1, ABL1 | 0.266144027 |
| GOTERM_MF_DIRECT | GO:0019838~growth factor binding | 3 | 0.01 | 0.055980384 | KDR, TEK, TGFBR1 | 0.266144027 |
| GOTERM_MF_DIRECT | GO:0042056~chemoattractant activity | 3 | 0.01 | 0.055980384 | LGALS3, CCL5, MIF | 0.266144027 |
| GOTERM_MF_DIRECT | GO:0030246~carbohydrate binding | 7 | 0.02 | 0.061661705 | SELP, APCS, LGALS3, LGALS2, CD209, REG1A, IL2 | 0.290690894 |
| GOTERM_MF_DIRECT | GO:0004712~protein serine/threonine/tyrosine kinase activity | 3 | 0.01 | 0.063576617 | MAP2K1, AKT1, PRKACA | 0.293679443 |
| GOTERM_MF_DIRECT | GO:0042169~SH2 domain binding | 3 | 0.01 | 0.063576617 | SRC, LCK, JAK2 | 0.293679443 |
| GOTERM_MF_DIRECT | GO:0003713~transcription coactivator activity | 8 | 0.02 | 0.064451837 | RXRB, RXRA, NR1I3, NR1I2, NR1H4, NR1H3, ESR2, PPARD | 0.293679443 |
| GOTERM_MF_DIRECT | GO:0005506~iron ion binding | 6 | 0.02 | 0.067579103 | CYP2C9, CYP2C8, TPH1, NOS3, PAH, LCN2 | 0.293679443 |
| GOTERM_MF_DIRECT | GO:0030284~estrogen receptor activity | 2 | 0.01 | 0.069101045 | ESR1, ESR2 | 0.293679443 |
| GOTERM_MF_DIRECT | GO:0019158~mannokinase activity | 2 | 0.01 | 0.069101045 | GCK, HK1 | 0.293679443 |
| GOTERM_MF_DIRECT | GO:0008865~fructokinase activity | 2 | 0.01 | 0.069101045 | GCK, HK1 | 0.293679443 |
| GOTERM_MF_DIRECT | GO:0005007~fibroblast growth factor-activated receptor activity | 2 | 0.01 | 0.069101045 | FGFR2, FGFR1 | 0.293679443 |
| GOTERM_MF_DIRECT | GO:0004396~hexokinase activity | 2 | 0.01 | 0.069101045 | GCK, HK1 | 0.293679443 |
| GOTERM_MF_DIRECT | GO:0004340~glucokinase activity | 2 | 0.01 | 0.069101045 | GCK, HK1 | 0.293679443 |
| GOTERM_MF_DIRECT | GO:0050544~arachidonic acid binding | 2 | 0.01 | 0.069101045 | PPARG, S100A9 | 0.293679443 |
| GOTERM_MF_DIRECT | GO:0051525~NFAT protein binding | 2 | 0.01 | 0.069101045 | MAPK14, PPARA | 0.293679443 |
| GOTERM_MF_DIRECT | GO:0004784~superoxide dismutase activity | 2 | 0.01 | 0.069101045 | NQO1, SOD2 | 0.293679443 |
| GOTERM_MF_DIRECT | GO:0051082~unfolded protein binding | 5 | 0.01 | 0.071911409 | HSPA8, APCS, HSP90AA1, PPIA, HSPA1A | 0.303325566 |
| GOTERM_MF_DIRECT | GO:0000977~RNA polymerase II regulatory region sequence-specific DNA binding | 7 | 0.02 | 0.077243774 | RXRB, RXRA, NR1I3, NR1I2, RARB, ESRRG, RORA | 0.322932343 |
| GOTERM_MF_DIRECT | GO:0001618~virus receptor activity | 4 | 0.01 | 0.077710992 | DPP4, ACE2, CD209, HSPA1A | 0.322932343 |
| GOTERM_MF_DIRECT | GO:0070492~oligosaccharide binding | 2 | 0.01 | 0.082339713 | SELP, SELE | 0.327606942 |
| GOTERM_MF_DIRECT | GO:0005168~neurotrophin TRKA receptor binding | 2 | 0.01 | 0.082339713 | GRB2, PIK3R1 | 0.327606942 |
| GOTERM_MF_DIRECT | GO:0055131~C3HC4-type RING finger domain binding | 2 | 0.01 | 0.082339713 | HSPA8, HSPA1A | 0.327606942 |
| GOTERM_MF_DIRECT | GO:0004024~alcohol dehydrogenase activity, zinc-dependent | 2 | 0.01 | 0.082339713 | ADH1C, ADH1B | 0.327606942 |
| GOTERM_MF_DIRECT | GO:0004064~arylesterase activity | 2 | 0.01 | 0.082339713 | CA1, CA2 | 0.327606942 |
| GOTERM_MF_DIRECT | GO:0070324~thyroid hormone binding | 2 | 0.01 | 0.082339713 | TTR, THRA | 0.327606942 |
| GOTERM_MF_DIRECT | GO:0001540~beta-amyloid binding | 3 | 0.01 | 0.083932032 | BACE1, BCHE, TGFB2 | 0.331590633 |
| GOTERM_MF_DIRECT | GO:0004364~glutathione transferase activity | 3 | 0.01 | 0.088213594 | GSTM1, GSTO1, GSTP1 | 0.346068714 |
| GOTERM_MF_DIRECT | GO:0034618~arginine binding | 2 | 0.01 | 0.095390881 | NOS2, NOS3 | 0.361583003 |
| GOTERM_MF_DIRECT | GO:0036094~small molecule binding | 2 | 0.01 | 0.095390881 | RBP4, LCN2 | 0.361583003 |
| GOTERM_MF_DIRECT | GO:0005114~type II transforming growth factor beta receptor binding | 2 | 0.01 | 0.095390881 | TGFB2, TGFBR1 | 0.361583003 |
| GOTERM_MF_DIRECT | GO:0004022~alcohol dehydrogenase (NAD) activity | 2 | 0.01 | 0.095390881 | ADH1C, ADH5 | 0.361583003 |
| GOTERM_MF_DIRECT | GO:0005024~transforming growth factor beta-activated receptor activity | 2 | 0.01 | 0.095390881 | TGFBR1, TGFBR2 | 0.361583003 |
| GOTERM_MF_DIRECT | GO:0030331~estrogen receptor binding | 3 | 0.01 | 0.096965039 | PARP1, SRC, PPARG | 0.365083133 |
