# Supplementary material for: A Network Pharmacology Approach for Exploring the Mechanisms of Panax notoginseng Saponins in Ischaemic Stroke
Source: Evid Based Complement Alternat Med. 2021 Aug 13;2021:5582782. doi: 10.1155/2021/5582782 (PMC8382556; doi:10.1155/2021/5582782)
Supplement: Supplementary Materials — The supplementary materials are available online. Table S1: basic information of ingredients in PNS; Table S2: functions of potential target genes based on the GO molecular function; Table S3: functions of potential target genes based on the GO biological process; Table S4: functions of potential target genes based on the GO cellular component; Table S5: functions of potential target genes based on KEGG analysis. [file 5582782.f1.zip › 5582782.f1/Supplementary Table S3 Functions of potential target genes based on GO biological process.docx]

Supplementary Table 3 Functions of potential target genes based on GO biological process

| Category | Term | Count | Percent | PValue | Genes | FDR |
| --- | --- | --- | --- | --- | --- | --- |
| GOTERM_BP_DIRECT | GO:0043401~steroid hormone mediated signaling pathway | 20 | 0.05 | 1.41E-21 | ESRRA, THRA, VDR, NR1H2, NR1I3, NR1I2, NR1H4, NR1H3, RORA, ESRRG, BMP7, ESR1, NR3C2, RXRB, RXRA, RARB, PGR, PPARG, PPARA, PPARD | 3.41E-18 |
| GOTERM_BP_DIRECT | GO:0006367~transcription initiation from RNA polymerase II promoter | 22 | 0.06 | 3.72E-15 | ESRRA, THRA, VDR, NR1H2, NR1I3, NR1I2, NR1H4, NR1H3, RORA, ESRRG, NR3C1, ESR1, ESR2, NR3C2, RXRB, AR, RXRA, RARB, PGR, PPARG, PPARA, PPARD | 4.49E-12 |
| GOTERM_BP_DIRECT | GO:0038083~peptidyl-tyrosine autophosphorylation | 13 | 0.04 | 1.96E-13 | SYK, SRC, INSR, IGF1R, HCK, ZAP70, ERBB4, LCK, KDR, ABL1, CSK, JAK2, JAK3 | 1.58E-10 |
| GOTERM_BP_DIRECT | GO:0046777~protein autophosphorylation | 21 | 0.06 | 4.76E-13 | GSK3B, SYK, DAPK1, PDPK1, SRC, INSR, EGFR, IGF1R, HCK, ERBB4, KIT, KDR, ABL1, AKT1, CSK, TEK, JAK2, PRKACA, EPHB4, FGFR2, FGFR1 | 2.88E-10 |
| GOTERM_BP_DIRECT | GO:0048015~phosphatidylinositol-mediated signaling | 17 | 0.05 | 1.90E-12 | PDPK1, NPR3, PTPN11, PIK3R1, IGF1, RHOA, EGFR, PIK3CG, IGF1R, ERBB4, LCK, KIT, NCS1, AKT1, GRB2, FGFR2, FGFR1 | 9.20E-10 |
| GOTERM_BP_DIRECT | GO:0018108~peptidyl-tyrosine phosphorylation | 19 | 0.05 | 5.98E-12 | MAP2K1, HSP90AA1, SRC, INSR, EGFR, HCK, ZAP70, ERBB4, KIT, KDR, ABL1, TEK, JAK2, JAK3, MET, EPHB4, FGFR2, EPHA2, FGFR1 | 2.41E-09 |
| GOTERM_BP_DIRECT | GO:0042493~response to drug | 25 | 0.07 | 1.01E-11 | MAOB, FECH, SRC, PNP, CASP3, ABL1, HADH, BCHE, TGFB2, HSP90AA1, STAT1, ARG1, APOA2, SOD2, PGF, DUSP6, TGFBR2, ADAM17, FABP3, ADORA2A, LCK, LCN2, MDM2, PPARG, OTC | 3.49E-09 |
| GOTERM_BP_DIRECT | GO:0050900~leukocyte migration | 17 | 0.05 | 1.74E-11 | MMP1, SRC, PTPN11, PIK3R1, MIF, F2, ITGAL, SELE, MMP9, SELP, PROCR, LCK, PDE4B, GRB2, TEK, HRAS, PPIA | 5.25E-09 |
| GOTERM_BP_DIRECT | GO:0006508~proteolysis | 31 | 0.08 | 2.49E-11 | CFD, C1S, C1R, CTSS, DPP4, ADAMTS4, CASP7, PLAU, CASP3, CASP1, CTSG, LTA4H, CTSD, ELANE, CTSB, MMP7, MME, MMP1, MMP2, MMP3, F11, MMP8, F2, MMP9, BACE1, MMP12, ADAM17, MMP13, FAP, REN, CFB | 6.68E-09 |
| GOTERM_BP_DIRECT | GO:0007165~signal transduction | 48 | 0.13 | 4.54E-11 | SPARC, SRC, NR1I3, PDE3B, NR1I2, PIK3R1, NR3C1, ITGAL, EGFR, NR3C2, IGF1R, HINT1, IMPA1, PLAU, ERBB4, AKT2, CASP1, PDE4B, RAC2, AKT1, MAPK1, JAK2, HRAS, MAP2K1, HSP90AA1, CSNK2A1, DAPK1, VDR, PDE4D, ANXA5, NR1H4, IGF1, MAPK14, ESR1, TGFBR1, PGF, ESR2, MAPK10, AR, RHEB, KIT, RARB, PDE5A, PPARG, PGR, TEK, S100A9, MET | 1.10E-08 |
| GOTERM_BP_DIRECT | GO:0032869~cellular response to insulin stimulus | 14 | 0.04 | 5.53E-11 | PARP1, PDPK1, STAT1, SRC, GSTP1, INSR, PDE3B, PIK3R1, GCK, APRT, AKT2, AKT1, PPARG, PCK1 | 1.21E-08 |
| GOTERM_BP_DIRECT | GO:0043066~negative regulation of apoptotic process | 29 | 0.08 | 6.73E-11 | GSK3B, SRC, GSTP1, GLO1, XIAP, PIK3R1, EGFR, IGF1R, MAPK8, ERBB4, CASP3, KDR, AKT1, TGM2, NQO1, ANXA5, NR1H4, IGF1, MIF, SOD2, MMP9, IL2, HCK, ALB, MDM2, RARB, TEK, BCL2L1, PPARD | 1.36E-08 |
| GOTERM_BP_DIRECT | GO:0043627~response to estrogen | 13 | 0.04 | 1.03E-10 | ARSA, HSP90AA1, GBA, APOA2, ESR1, TGFBR2, F7, CA2, CTNNA1, HMOX1, MAPK1, PPARG, TEK | 1.92E-08 |
| GOTERM_BP_DIRECT | GO:0001666~response to hypoxia | 18 | 0.05 | 3.77E-10 | TGFB2, AHCY, NOS2, MMP2, SOD2, PGF, TGFBR2, DPP4, F7, BMP2, ADAM17, PLAU, CASP3, CASP1, HMOX1, ANG, TEK, PPARA | 6.52E-08 |
| GOTERM_BP_DIRECT | GO:0048661~positive regulation of smooth muscle cell proliferation | 12 | 0.03 | 6.85E-10 | STAT1, CCL5, AKR1B1, HMOX1, AKT1, HMGCR, IGF1, EGFR, ELANE, FGFR2, TGFBR2, TGM2 | 1.05E-07 |
| GOTERM_BP_DIRECT | GO:0022617~extracellular matrix disassembly | 13 | 0.04 | 6.94E-10 | MMP7, MMP1, CMA1, MMP2, MMP3, MMP8, MMP9, CTSS, MMP12, ADAMTS4, MMP13, CTSG, ELANE | 1.05E-07 |
| GOTERM_BP_DIRECT | GO:0030335~positive regulation of cell migration | 18 | 0.05 | 1.08E-09 | F10, INSR, PIK3R1, IGF1, EGFR, TGFBR1, IGF1R, F7, BMP2, ADAM17, PLAU, AKT2, CCL5, KIT, KDR, MAPK1, JAK2, HRAS | 1.53E-07 |
| GOTERM_BP_DIRECT | GO:0014068~positive regulation of phosphatidylinositol 3-kinase signaling | 12 | 0.03 | 1.68E-09 | SELP, TGFB2, ERBB4, CCL5, KIT, KDR, TEK, IGF1, F2, JAK2, FGFR1, PPARD | 2.26E-07 |
| GOTERM_BP_DIRECT | GO:0018107~peptidyl-threonine phosphorylation | 10 | 0.03 | 2.26E-09 | GSK3B, MAPK8, PDPK1, CHEK1, MAPK1, AKT1, PRKACA, TGFBR1, CDK5R1, TGFBR2 | 2.88E-07 |
| GOTERM_BP_DIRECT | GO:0008284~positive regulation of cell proliferation | 27 | 0.07 | 2.72E-09 | REG1A, EGFR, IGF1R, DPP4, ERBB4, KDR, RAC2, MAPK1, HRAS, TGFB2, CSNK2A1, INSR, IGF1, F2, TGFBR1, IL2, PGF, TGFBR2, AR, HCK, ADAM17, KIT, MDM2, RARB, FGFR2, FGFR1, BCL2L1 | 3.29E-07 |
| GOTERM_BP_DIRECT | GO:0001525~angiogenesis | 19 | 0.05 | 3.03E-09 | GPI, TGFB2, SYK, NOS3, MMP2, PDE3B, RORA, MAPK14, PGF, PIK3CG, TYMP, FAP, KDR, HMOX1, ANG, TEK, EPHB4, FGFR2, FGFR1 | 3.49E-07 |
| GOTERM_BP_DIRECT | GO:0007169~transmembrane receptor protein tyrosine kinase signaling pathway | 13 | 0.04 | 1.09E-08 | SYK, INSR, EGFR, IGF1R, HCK, ZAP70, ERBB4, LCK, KIT, KDR, CSK, TEK, MET | 1.20E-06 |
| GOTERM_BP_DIRECT | GO:0030574~collagen catabolic process | 11 | 0.03 | 2.10E-08 | MMP12, MMP13, MMP7, MMP1, MMP2, MMP3, MMP8, CTSD, MMP9, CTSS, CTSB | 2.21E-06 |
| GOTERM_BP_DIRECT | GO:0006468~protein phosphorylation | 25 | 0.07 | 3.52E-08 | GSK3B, PIK3R1, PIK3CG, MAPK8, AKT1, MAPK1, CSK, CTSG, JAK2, PRKACA, JAK3, TGFB2, SYK, CSNK2A1, DAPK1, PDPK1, TGFBR1, TGFBR2, MAPK10, HCK, ZAP70, BMP2, CDK6, LCK, FGFR1 | 3.54E-06 |
| GOTERM_BP_DIRECT | GO:0010628~positive regulation of gene expression | 19 | 0.05 | 3.80E-08 | TGFB2, MAP2K1, VDR, EPHX2, GBA, NR1I2, MAPK14, TGFBR1, CDC42, AR, BMP2, MAPK8, CDK6, KIT, MDM2, LCN2, HRAS, HSPA1A, PPARD | 3.68E-06 |
| GOTERM_BP_DIRECT | GO:0030522~intracellular receptor signaling pathway | 9 | 0.02 | 4.91E-08 | ESRRA, AR, THRA, NR1I2, NR1H4, NR1H3, RORA, PPARA, PPARD | 4.56E-06 |
| GOTERM_BP_DIRECT | GO:0071222~cellular response to lipopolysaccharide | 13 | 0.04 | 6.96E-08 | NOS2, SRC, ARG1, GSTP1, PDE4D, NR1H4, NR1H3, MAPK14, MAPK8, PDE4B, ABL1, LCN2, PPARD | 6.23E-06 |
| GOTERM_BP_DIRECT | GO:0007173~epidermal growth factor receptor signaling pathway | 10 | 0.03 | 8.42E-08 | ADAM17, PDPK1, SRC, ABL1, CSK, GRB2, PTPN11, PIK3R1, HRAS, EGFR | 7.06E-06 |
| GOTERM_BP_DIRECT | GO:0030168~platelet activation | 13 | 0.04 | 8.47E-08 | SYK, PDPK1, SRC, PTPN11, GP1BA, PIK3R1, F2, RHOA, PIK3CG, LCK, RAC2, AKT1, MAPK1 | 7.06E-06 |
| GOTERM_BP_DIRECT | GO:0043406~positive regulation of MAP kinase activity | 10 | 0.03 | 1.35E-07 | SRC, KIT, CSK, PDE5A, MIF, HRAS, EGFR, ELANE, PIK3CG, FGFR1 | 1.08E-05 |
| GOTERM_BP_DIRECT | GO:0014066~regulation of phosphatidylinositol 3-kinase signaling | 11 | 0.03 | 1.47E-07 | ERBB4, LCK, KIT, MAPK1, AKT1, GRB2, PTPN11, PIK3R1, EGFR, FGFR2, FGFR1 | 1.14E-05 |
| GOTERM_BP_DIRECT | GO:0070374~positive regulation of ERK1 and ERK2 cascade | 15 | 0.04 | 2.02E-07 | MAP2K1, SRC, PLA2G2A, PTPN11, HMGCR, MIF, EGFR, BMP2, ERBB4, CCL5, KDR, ABL1, TEK, HRAS, FGFR2 | 1.53E-05 |
| GOTERM_BP_DIRECT | GO:0018105~peptidyl-serine phosphorylation | 13 | 0.04 | 2.14E-07 | GSK3B, SYK, PDPK1, SRC, MAPK14, TGFBR1, TGFBR2, MAPK8, AKT2, AKT1, MAPK1, PRKACA, CDK5R1 | 1.57E-05 |
| GOTERM_BP_DIRECT | GO:0001934~positive regulation of protein phosphorylation | 13 | 0.04 | 2.54E-07 | INSR, F2, MMP9, EGFR, BMP2, ADAM17, ERBB4, AKT2, KDR, ABL1, AKT1, TEK, HRAS | 1.81E-05 |
| GOTERM_BP_DIRECT | GO:0007507~heart development | 15 | 0.04 | 3.50E-07 | TGFB2, MAP2K1, SPARC, PTPN11, SOD2, TGFBR1, TGFBR2, CASP7, BMP2, RBP4, ERBB4, PPARG, TEK, PPARA, PPARD | 2.42E-05 |
| GOTERM_BP_DIRECT | GO:0032496~response to lipopolysaccharide | 14 | 0.04 | 6.16E-07 | SPARC, MAOB, SOD2, SELE, ADH5, SELP, ADAM17, CASP3, CASP1, CTSG, REN, JAK2, PCK1, ELANE | 4.14E-05 |
| GOTERM_BP_DIRECT | GO:0006809~nitric oxide biosynthetic process | 6 | 0.02 | 6.43E-07 | NQO1, ARG2, NOS2, NOS3, AKT1, RORA | 4.20E-05 |
| GOTERM_BP_DIRECT | GO:0046854~phosphatidylinositol phosphorylation | 11 | 0.03 | 8.64E-07 | IMPA1, ERBB4, LCK, KIT, GRB2, PTPN11, PIK3R1, EGFR, FGFR2, PIK3CG, FGFR1 | 5.50E-05 |
| GOTERM_BP_DIRECT | GO:0038128~ERBB2 signaling pathway | 8 | 0.02 | 9.13E-07 | HSP90AA1, ERBB4, SRC, AKT1, GRB2, PIK3R1, HRAS, EGFR | 5.66E-05 |
| GOTERM_BP_DIRECT | GO:0051090~regulation of sequence-specific DNA binding transcription factor activity | 7 | 0.02 | 1.08E-06 | MAPK10, HCK, MAPK8, SYK, HMOX1, MAPK1, MAPK14 | 6.50E-05 |
| GOTERM_BP_DIRECT | GO:0031295~T cell costimulation | 10 | 0.03 | 1.55E-06 | CDC42, DPP4, PDPK1, SRC, LCK, AKT1, CSK, GRB2, PTPN11, PIK3R1 | 9.12E-05 |
| GOTERM_BP_DIRECT | GO:0050728~negative regulation of inflammatory response | 10 | 0.03 | 1.72E-06 | ADORA2A, GBA, NR1H4, NR1H3, RORA, TEK, PPARA, IL2, ELANE, PPARD | 9.92E-05 |
| GOTERM_BP_DIRECT | GO:0043410~positive regulation of MAPK cascade | 10 | 0.03 | 2.13E-06 | AR, BMP2, INSR, KIT, PSAP, KDR, IGF1, HRAS, FGFR2, FGFR1 | 1.20E-04 |
| GOTERM_BP_DIRECT | GO:0007596~blood coagulation | 14 | 0.04 | 2.26E-06 | SERPINA1, F10, F11, ANXA5, GP1BA, F2, CDC42, F7, PROCR, ADORA2A, PLAU, JAK2, PRKACA, RAB5A | 1.24E-04 |
| GOTERM_BP_DIRECT | GO:0048762~mesenchymal cell differentiation | 5 | 0.01 | 2.63E-06 | BMP2, BMP7, TGFBR1, FGFR2, FGFR1 | 1.41E-04 |
| GOTERM_BP_DIRECT | GO:0050727~regulation of inflammatory response | 9 | 0.02 | 2.86E-06 | ACE2, HCK, FABP4, CMA1, CASP1, XIAP, JAK2, SELE, ESR1 | 1.49E-04 |
| GOTERM_BP_DIRECT | GO:0030307~positive regulation of cell growth | 10 | 0.03 | 2.90E-06 | CDC42, TGFB2, ADAM17, CSNK2A1, AKT1, F2, S100A9, EGFR, IL2, TGFBR1 | 1.49E-04 |
| GOTERM_BP_DIRECT | GO:0045087~innate immune response | 21 | 0.06 | 3.46E-06 | APCS, SYK, C1S, C1R, SRC, NR1H4, MIF, PIK3CG, ZAP70, LGALS3, CD209, LCK, ABL1, LCN2, CSK, ANG, PPARG, PADI4, JAK2, JAK3, S100A9 | 1.74E-04 |
| GOTERM_BP_DIRECT | GO:0048013~ephrin receptor signaling pathway | 10 | 0.03 | 3.53E-06 | CDC42, SRC, MMP2, PTPN11, HRAS, MMP9, EPHB4, RHOA, EPHA2, CDK5R1 | 1.74E-04 |
| GOTERM_BP_DIRECT | GO:0000165~MAPK cascade | 16 | 0.04 | 5.19E-06 | MAP2K1, EGFR, IL2, DUSP6, ERBB4, CCL5, KIT, MAPK1, GRB2, TEK, CALM1, JAK2, HRAS, JAK3, FGFR2, FGFR1 | 2.51E-04 |
| GOTERM_BP_DIRECT | GO:0044267~cellular protein metabolic process | 11 | 0.03 | 6.92E-06 | BACE1, APCS, MMP13, TTR, ADORA2A, MMP1, MMP2, CTSG, IGF1, F2, LYZ | 3.28E-04 |
| GOTERM_BP_DIRECT | GO:0045944~positive regulation of transcription from RNA polymerase II promoter | 33 | 0.09 | 9.07E-06 | GSK3B, THRA, NR1I3, NR1I2, RORA, PIK3R1, NR3C1, EGFR, RXRB, RXRA, AKT1, HRAS, ESRRA, PARP1, STAT1, VDR, NR1H2, NR1H4, NR1H3, ESRRG, IGF1, MAPK14, BMP7, ESR1, IL2, AR, BMP2, RARB, PPARG, PGR, PPARA, MET, FGFR2 | 4.22E-04 |
| GOTERM_BP_DIRECT | GO:0035902~response to immobilization stress | 6 | 0.02 | 9.27E-06 | GPI, TPH1, REN, PPARG, HNMT, SOD2 | 4.23E-04 |
| GOTERM_BP_DIRECT | GO:0002003~angiotensin maturation | 5 | 0.01 | 1.20E-05 | ACE2, MME, CMA1, REN, CTSG | 5.38E-04 |
| GOTERM_BP_DIRECT | GO:0042127~regulation of cell proliferation | 13 | 0.04 | 1.33E-05 | ESRRA, NOS2, SRC, XIAP, TGFBR2, ACE2, PLAU, LCK, KIT, CHEK1, ABL1, CSK, JAK2 | 5.82E-04 |
| GOTERM_BP_DIRECT | GO:0045893~positive regulation of transcription, DNA-templated | 22 | 0.06 | 1.47E-05 | MAP2K1, STAT1, SRC, NR1H2, INSR, NR1I2, NR1H3, RORA, ESRRG, IGF1, BMP7, ESR1, TGFBR1, ESR2, SEC14L2, AR, BMP2, ERBB4, MAPK1, PPARG, PPARA, PPARD | 6.35E-04 |
| GOTERM_BP_DIRECT | GO:0045471~response to ethanol | 10 | 0.03 | 1.83E-05 | NQO1, ARSA, G6PD, RBP4, SPARC, MAOB, FECH, GSTP1, HMGCR, IL2 | 7.76E-04 |
| GOTERM_BP_DIRECT | GO:0000187~activation of MAPK activity | 10 | 0.03 | 2.13E-05 | MAPK10, MAP2K1, BMP2, INSR, KIT, MAPK1, PTPN11, IGF1, MAPK14, DUSP6 | 8.88E-04 |
| GOTERM_BP_DIRECT | GO:0007159~leukocyte cell-cell adhesion | 6 | 0.02 | 2.31E-05 | SELP, SYK, CD209, CCL5, ITGAL, SELE | 9.47E-04 |
| GOTERM_BP_DIRECT | GO:0001501~skeletal system development | 11 | 0.03 | 2.58E-05 | ADAMTS4, TGFB2, BMP2, VDR, NPR3, IGF1, MMP9, BMP7, TGFBR1, EPHA2, FGFR1 | 0.001038196 |
| GOTERM_BP_DIRECT | GO:0009636~response to toxic substance | 9 | 0.02 | 2.72E-05 | NQO1, MAOB, CCL5, EPHX2, GSTP1, TTPA, MDM2, MAPK1, CES1 | 0.001079126 |
| GOTERM_BP_DIRECT | GO:0045429~positive regulation of nitric oxide biosynthetic process | 7 | 0.02 | 2.99E-05 | HSP90AA1, INSR, AKT1, JAK2, SOD2, ESR1, EGFR | 0.00115 |
| GOTERM_BP_DIRECT | GO:0006954~inflammatory response | 18 | 0.05 | 3.00E-05 | SYK, EPHX2, NR1H4, MIF, ITGAL, LYZ, SELE, PIK3CG, SELP, HCK, ZAP70, BMP2, ADORA2A, CCL5, KIT, AKT1, S100A9, EPHA2 | 0.00115 |
| GOTERM_BP_DIRECT | GO:0016477~cell migration | 12 | 0.03 | 3.48E-05 | GSK3B, TGFB2, ERBB4, PDPK1, ABL1, CSK, ANG, JAK2, JAK3, RHOA, EPHA2, FGFR1 | 0.001313145 |
| GOTERM_BP_DIRECT | GO:0030593~neutrophil chemotaxis | 8 | 0.02 | 4.02E-05 | LGALS3, TGFB2, SYK, CCL5, PDE4D, PDE4B, S100A9, PIK3CG | 0.001495888 |
| GOTERM_BP_DIRECT | GO:0045725~positive regulation of glycogen biosynthetic process | 5 | 0.01 | 4.75E-05 | AKT2, INSR, AKT1, IGF1, GCK | 0.001739646 |
| GOTERM_BP_DIRECT | GO:0043542~endothelial cell migration | 6 | 0.02 | 4.93E-05 | DPP4, FAP, STAT1, NOS3, RHOA, TGFBR1 | 0.001753205 |
| GOTERM_BP_DIRECT | GO:0045909~positive regulation of vasodilation | 6 | 0.02 | 4.93E-05 | NOS2, NOS3, EPHX2, HMOX1, EGFR, PPARD | 0.001753205 |
| GOTERM_BP_DIRECT | GO:0008285~negative regulation of cell proliferation | 18 | 0.05 | 5.23E-05 | BCHE, TGFB2, MAP2K1, NOS3, VDR, SOD2, BMP7, AR, BMP2, FABP3, CDK6, ADORA2A, ERBB4, FABP7, RARB, CSK, JAK2, HRAS | 0.001831341 |
| GOTERM_BP_DIRECT | GO:0001938~positive regulation of endothelial cell proliferation | 8 | 0.02 | 5.38E-05 | BMP2, ARG1, KDR, AKT1, ANG, TEK, TGFBR1, PGF | 0.001854877 |
| GOTERM_BP_DIRECT | GO:0010887~negative regulation of cholesterol storage | 4 | 0.01 | 5.45E-05 | NR1H2, NR1H3, PPARG, PPARA | 0.001854877 |
| GOTERM_BP_DIRECT | GO:0046326~positive regulation of glucose import | 6 | 0.02 | 5.85E-05 | AKT2, INSR, AKT1, IGF1, PIK3R1, MAPK14 | 0.001964146 |
| GOTERM_BP_DIRECT | GO:0048010~vascular endothelial growth factor receptor signaling pathway | 8 | 0.02 | 7.08E-05 | CDC42, HSP90AA1, SRC, KDR, PIK3R1, MAPK14, RHOA, PGF | 0.002344436 |
| GOTERM_BP_DIRECT | GO:0050729~positive regulation of inflammatory response | 8 | 0.02 | 7.74E-05 | FABP4, CCL5, PLA2G2A, JAK2, S100A9, EGFR, IL2, TGM2 | 0.002527554 |
| GOTERM_BP_DIRECT | GO:0031663~lipopolysaccharide-mediated signaling pathway | 6 | 0.02 | 8.08E-05 | HCK, NOS3, CCL5, MAPK1, AKT1, MAPK14 | 0.002575987 |
| GOTERM_BP_DIRECT | GO:0042523~positive regulation of tyrosine phosphorylation of Stat5 protein | 5 | 0.01 | 8.10E-05 | ERBB4, KIT, IGF1, JAK2, IL2 | 0.002575987 |
| GOTERM_BP_DIRECT | GO:0007584~response to nutrient | 8 | 0.02 | 8.45E-05 | NQO1, ARSA, AHCY, STAT1, TTPA, PPARG, HMGCR, TGFBR2 | 0.002650888 |
| GOTERM_BP_DIRECT | GO:0042593~glucose homeostasis | 9 | 0.02 | 9.42E-05 | GPI, RBP4, INSR, NR1H4, AKT1, PTPN11, PPARG, PCK1, GCK | 0.002886757 |
| GOTERM_BP_DIRECT | GO:0060687~regulation of branching involved in prostate gland morphogenesis | 4 | 0.01 | 9.44E-05 | RXRA, ESR1, BMP7, FGFR2 | 0.002886757 |
| GOTERM_BP_DIRECT | GO:0048384~retinoic acid receptor signaling pathway | 5 | 0.01 | 1.03E-04 | RXRB, RXRA, NR1H2, RARB, ESRRG | 0.003111495 |
| GOTERM_BP_DIRECT | GO:0002576~platelet degranulation | 9 | 0.02 | 1.08E-04 | SELP, CFD, TGFB2, SERPINA1, SPARC, ALB, PSAP, IGF1, CALM1 | 0.003215528 |
| GOTERM_BP_DIRECT | GO:0001837~epithelial to mesenchymal transition | 6 | 0.02 | 1.09E-04 | GSK3B, TGFB2, BMP2, BMP7, TGFBR1, FGFR2 | 0.003215528 |
| GOTERM_BP_DIRECT | GO:0006805~xenobiotic metabolic process | 8 | 0.02 | 1.18E-04 | NQO1, CYP2C9, CYP2C8, EPHX2, GSTP1, NR1I2, RORA, CES1 | 0.003441368 |
| GOTERM_BP_DIRECT | GO:0007568~aging | 11 | 0.03 | 1.23E-04 | NQO1, CASP7, MMP7, ARG1, PDE4D, CTNNA1, AKT1, GRB2, HMGCR, PCK1, TGFBR2 | 0.003541673 |
| GOTERM_BP_DIRECT | GO:0006749~glutathione metabolic process | 7 | 0.02 | 1.37E-04 | G6PD, GSTM1, GSTO1, GLO1, GSTP1, GSR, SOD2 | 0.003886098 |
| GOTERM_BP_DIRECT | GO:0042060~wound healing | 8 | 0.02 | 1.39E-04 | TGFB2, SPARC, CASP3, PPARA, EGFR, TGFBR1, TGFBR2, PPARD | 0.003899139 |
| GOTERM_BP_DIRECT | GO:0050714~positive regulation of protein secretion | 6 | 0.02 | 1.44E-04 | TGFB2, ARF1, ADORA2A, ANG, IGF1, PPIA | 0.004012528 |
| GOTERM_BP_DIRECT | GO:0010629~negative regulation of gene expression | 10 | 0.03 | 1.47E-04 | CDC42, TGFB2, MAP2K1, NOS2, GBA, AKT1, PGR, MIF, HRAS, ESR1 | 0.004049951 |
| GOTERM_BP_DIRECT | GO:2001237~negative regulation of extrinsic apoptotic signaling pathway | 6 | 0.02 | 1.88E-04 | LGALS3, AR, SRC, GSTP1, IGF1, TGFBR1 | 0.005063924 |
| GOTERM_BP_DIRECT | GO:0051897~positive regulation of protein kinase B signaling | 8 | 0.02 | 1.89E-04 | F7, F10, SRC, INSR, TEK, EGFR, TGFBR1, PIK3CG | 0.005063924 |
| GOTERM_BP_DIRECT | GO:0060020~Bergmann glial cell differentiation | 4 | 0.01 | 2.22E-04 | MAP2K1, ABL1, MAPK1, PTPN11 | 0.005825346 |
| GOTERM_BP_DIRECT | GO:2000188~regulation of cholesterol homeostasis | 4 | 0.01 | 2.22E-04 | NR1H2, NR1H4, NR1H3, RORA | 0.005825346 |
| GOTERM_BP_DIRECT | GO:0051000~positive regulation of nitric-oxide synthase activity | 5 | 0.01 | 2.35E-04 | DHFR, NPR3, AKT1, CALM1, ESR1 | 0.006119553 |
| GOTERM_BP_DIRECT | GO:0043124~negative regulation of I-kappaB kinase/NF-kappaB signaling | 6 | 0.02 | 2.41E-04 | STAT1, GSTP1, NR1H4, ABL1, RORA, ESR1 | 0.006190059 |
| GOTERM_BP_DIRECT | GO:0007267~cell-cell signaling | 13 | 0.04 | 2.81E-04 | TGFB2, IL2, PGF, ESR2, AR, BMP2, ADORA2A, CCL5, GRB2, PGR, TEK, S100A9, FGFR2 | 0.007147138 |
| GOTERM_BP_DIRECT | GO:0045740~positive regulation of DNA replication | 6 | 0.02 | 3.04E-04 | CDC42, INSR, IGF1, HRAS, EGFR, IGF1R | 0.007501104 |
| GOTERM_BP_DIRECT | GO:0055114~oxidation-reduction process | 21 | 0.06 | 3.05E-04 | NQO1, G6PD, TPH1, MAOB, NOS2, GSTO1, NOS3, GSR, AKR1B1, HMGCR, SOD2, DHFR, HSD11B1, CYP2C9, BMP2, CYP2C8, CBS, PAH, PNPO, BLVRB, ACADM | 0.007501104 |
| GOTERM_BP_DIRECT | GO:0032355~response to estradiol | 8 | 0.02 | 3.10E-04 | ESRRA, NQO1, GPI, F7, CASP3, GSTP1, ESR1, BMP7 | 0.007501104 |
| GOTERM_BP_DIRECT | GO:0008217~regulation of blood pressure | 7 | 0.02 | 3.13E-04 | NOS3, EPHX2, NPR3, HMOX1, REN, PPARG, SOD2 | 0.007501104 |
| GOTERM_BP_DIRECT | GO:0051384~response to glucocorticoid | 7 | 0.02 | 3.13E-04 | BCHE, SPARC, CASP3, GBA, APOA2, HNMT, AGXT | 0.007501104 |
| GOTERM_BP_DIRECT | GO:0051156~glucose 6-phosphate metabolic process | 4 | 0.01 | 3.13E-04 | GPI, G6PD, GCK, HK1 | 0.007501104 |
| GOTERM_BP_DIRECT | GO:0043525~positive regulation of neuron apoptotic process | 6 | 0.02 | 3.40E-04 | CDC42, NQO1, TGFB2, CASP3, ABL1, CDK5R1 | 0.008061999 |
| GOTERM_BP_DIRECT | GO:0006006~glucose metabolic process | 7 | 0.02 | 3.69E-04 | G6PD, FABP5, AKT2, AKT1, PCK1, MAPK14, PPARD | 0.008659478 |
| GOTERM_BP_DIRECT | GO:0008584~male gonad development | 8 | 0.02 | 3.78E-04 | TGFB2, INSR, KIT, CTNNA1, REN, ESR1, TGFBR1, BCL2L1 | 0.008787375 |
| GOTERM_BP_DIRECT | GO:0042327~positive regulation of phosphorylation | 5 | 0.01 | 3.94E-04 | AR, CCL5, ANG, MIF, EGFR | 0.009065676 |
| GOTERM_BP_DIRECT | GO:0006687~glycosphingolipid metabolic process | 6 | 0.02 | 4.22E-04 | ARSA, STS, GM2A, GBA, KIT, PSAP | 0.009428001 |
| GOTERM_BP_DIRECT | GO:0033628~regulation of cell adhesion mediated by integrin | 4 | 0.01 | 4.26E-04 | PLAU, PTPN11, PIK3CG, EPHA2 | 0.009428001 |
| GOTERM_BP_DIRECT | GO:0060445~branching involved in salivary gland morphogenesis | 4 | 0.01 | 4.26E-04 | BMP7, FGFR2, FGFR1, TGM2 | 0.009428001 |
| GOTERM_BP_DIRECT | GO:0003181~atrioventricular valve morphogenesis | 4 | 0.01 | 4.26E-04 | TGFB2, BMP2, MDM2, TGFBR2 | 0.009428001 |
| GOTERM_BP_DIRECT | GO:0071456~cellular response to hypoxia | 8 | 0.02 | 4.30E-04 | SRC, MDM2, HMOX1, AKT1, RORA, PCK1, BMP7, PPARD | 0.009428001 |
| GOTERM_BP_DIRECT | GO:0043154~negative regulation of cysteine-type endopeptidase activity involved in apoptotic process | 7 | 0.02 | 4.33E-04 | GPI, CSNK2A1, ADORA2A, SRC, MDM2, XIAP, AKT1 | 0.009428001 |
| GOTERM_BP_DIRECT | GO:0038096~Fc-gamma receptor signaling pathway involved in phagocytosis | 9 | 0.02 | 4.56E-04 | CDC42, HCK, HSP90AA1, SYK, SRC, ABL1, MAPK1, GRB2, PIK3R1 | 0.009759103 |
| GOTERM_BP_DIRECT | GO:0050999~regulation of nitric-oxide synthase activity | 5 | 0.01 | 4.60E-04 | HSP90AA1, NOS3, AKT1, CALM1, EGFR | 0.009759103 |
| GOTERM_BP_DIRECT | GO:0032148~activation of protein kinase B activity | 5 | 0.01 | 4.60E-04 | PDPK1, SRC, INSR, ANG, IGF1 | 0.009759103 |
| GOTERM_BP_DIRECT | GO:0045600~positive regulation of fat cell differentiation | 6 | 0.02 | 5.18E-04 | BMP2, TPH1, SULT1E1, AKT1, PPARG, PPARD | 0.010696736 |
| GOTERM_BP_DIRECT | GO:0031100~organ regeneration | 6 | 0.02 | 5.18E-04 | F7, GSTP1, APOA2, PPARG, PGF, TGFBR2 | 0.010696736 |
| GOTERM_BP_DIRECT | GO:0008630~intrinsic apoptotic signaling pathway in response to DNA damage | 6 | 0.02 | 5.18E-04 | ABL1, HMOX1, PIK3R1, SOD2, EPHA2, BCL2L1 | 0.010696736 |
| GOTERM_BP_DIRECT | GO:0009268~response to pH | 4 | 0.01 | 5.63E-04 | ARSA, CA2, TTPA, GBA | 0.011429321 |
| GOTERM_BP_DIRECT | GO:0006069~ethanol oxidation | 4 | 0.01 | 5.63E-04 | ALDH2, ADH1C, ADH1B, ADH5 | 0.011429321 |
| GOTERM_BP_DIRECT | GO:0033688~regulation of osteoblast proliferation | 3 | 0.01 | 5.95E-04 | NPR3, RHOA, FGFR2 | 0.011974963 |
| GOTERM_BP_DIRECT | GO:2000145~regulation of cell motility | 5 | 0.01 | 6.17E-04 | CDK6, ERBB4, ABL1, RHOA, EGFR | 0.012315948 |
| GOTERM_BP_DIRECT | GO:0036092~phosphatidylinositol-3-phosphate biosynthetic process | 6 | 0.02 | 6.29E-04 | GRB2, PTPN11, PIK3R1, FGFR2, PIK3CG, FGFR1 | 0.012462892 |
| GOTERM_BP_DIRECT | GO:0048662~negative regulation of smooth muscle cell proliferation | 5 | 0.01 | 7.07E-04 | NPR3, HMOX1, ANG, PPARG, PPARD | 0.013841593 |
| GOTERM_BP_DIRECT | GO:2001275~positive regulation of glucose import in response to insulin stimulus | 4 | 0.01 | 7.24E-04 | AKT2, NR1H4, PTPN11, PIK3R1 | 0.013841593 |
| GOTERM_BP_DIRECT | GO:0060389~pathway-restricted SMAD protein phosphorylation | 4 | 0.01 | 7.24E-04 | TGFB2, BMP2, TGFBR1, TGFBR2 | 0.013841593 |
| GOTERM_BP_DIRECT | GO:0010745~negative regulation of macrophage derived foam cell differentiation | 4 | 0.01 | 7.24E-04 | NR1H2, NR1H3, PPARG, PPARA | 0.013841593 |
| GOTERM_BP_DIRECT | GO:0030324~lung development | 7 | 0.02 | 7.27E-04 | HSD11B1, RBP4, SPARC, NOS3, ARG1, EGFR, FGFR2 | 0.013841593 |
| GOTERM_BP_DIRECT | GO:2000379~positive regulation of reactive oxygen species metabolic process | 5 | 0.01 | 8.07E-04 | ACE2, GRB2, F2, MAPK14, TGFBR2 | 0.015241007 |
| GOTERM_BP_DIRECT | GO:0008286~insulin receptor signaling pathway | 7 | 0.02 | 8.35E-04 | PTPN1, AKT2, INSR, AKT1, GRB2, PIK3R1, IGF1R | 0.01563886 |
| GOTERM_BP_DIRECT | GO:0048009~insulin-like growth factor receptor signaling pathway | 4 | 0.01 | 9.12E-04 | AKT1, IGF1, PIK3R1, IGF1R | 0.016655417 |
| GOTERM_BP_DIRECT | GO:0043101~purine-containing compound salvage | 4 | 0.01 | 9.12E-04 | MTAP, PNP, ADK, APRT | 0.016655417 |
| GOTERM_BP_DIRECT | GO:0060065~uterus development | 4 | 0.01 | 9.12E-04 | TGFB2, RBP4, SRC, ESR1 | 0.016655417 |
| GOTERM_BP_DIRECT | GO:0043552~positive regulation of phosphatidylinositol 3-kinase activity | 5 | 0.01 | 9.16E-04 | CDC42, ERBB4, SRC, KIT, TEK | 0.016655417 |
| GOTERM_BP_DIRECT | GO:0048146~positive regulation of fibroblast proliferation | 6 | 0.02 | 9.85E-04 | CDK6, ABL1, IGF1, MIF, ESR1, EGFR | 0.017769431 |
| GOTERM_BP_DIRECT | GO:1904707~positive regulation of vascular smooth muscle cell proliferation | 4 | 0.01 | 0.001127774 | MMP2, MDM2, JAK2, MMP9 | 0.01989656 |
| GOTERM_BP_DIRECT | GO:0042026~protein refolding | 4 | 0.01 | 0.001127774 | FKBP1A, HSPA8, HSP90AA1, HSPA1A | 0.01989656 |
| GOTERM_BP_DIRECT | GO:0060397~JAK-STAT cascade involved in growth hormone signaling pathway | 4 | 0.01 | 0.001127774 | PTPN1, MAPK1, JAK2, JAK3 | 0.01989656 |
| GOTERM_BP_DIRECT | GO:0015758~glucose transport | 5 | 0.01 | 0.001165779 | FABP5, AKT1, GCK, HK1, PPARD | 0.020418028 |
| GOTERM_BP_DIRECT | GO:0016032~viral process | 13 | 0.04 | 0.001191757 | HSPA8, SYK, MMP1, APOA2, PIK3R1, RHOA, HCK, LCK, MDM2, KDR, MAPK1, GRB2, EPHA2 | 0.020722848 |
| GOTERM_BP_DIRECT | GO:0006915~apoptotic process | 19 | 0.05 | 0.001239758 | CSNK2A1, DAPK1, STAT1, XIAP, MAPK14, NR3C1, TGFBR1, TGFBR2, CASP7, ADORA2A, CASP3, AKT2, CASP1, LCN2, MAPK1, JAK2, S100A9, FGFR2, PPARD | 0.021175106 |
| GOTERM_BP_DIRECT | GO:0002250~adaptive immune response | 9 | 0.02 | 0.001244048 | ZAP70, SYK, CD209, CSK, JAK2, JAK3, CTSS, IL2, PIK3CG | 0.021175106 |
| GOTERM_BP_DIRECT | GO:0050852~T cell receptor signaling pathway | 9 | 0.02 | 0.001244048 | ZAP70, PDPK1, LCK, PDE4D, PDE4B, MAPK1, CSK, PIK3R1, HRAS | 0.021175106 |
| GOTERM_BP_DIRECT | GO:0097192~extrinsic apoptotic signaling pathway in absence of ligand | 5 | 0.01 | 0.001306671 | GSK3B, CASP3, LCN2, IL2, BCL2L1 | 0.022085477 |
| GOTERM_BP_DIRECT | GO:0001701~in utero embryonic development | 10 | 0.03 | 0.001406177 | GPI, AR, BMP2, RXRA, NOS3, TGFBR1, FGFR2, FGFR1, BCL2L1, TGFBR2 | 0.023602294 |
| GOTERM_BP_DIRECT | GO:0071902~positive regulation of protein serine/threonine kinase activity | 5 | 0.01 | 0.001459039 | MAP2K1, SRC, CALM1, RHOA, CDK5R1 | 0.024320678 |
| GOTERM_BP_DIRECT | GO:0043407~negative regulation of MAP kinase activity | 5 | 0.01 | 0.001623365 | PTPN1, GSTP1, GBA, HMGCR, BMP7 | 0.026511313 |
| GOTERM_BP_DIRECT | GO:0007611~learning or memory | 5 | 0.01 | 0.001623365 | GPI, GM2A, THRA, CASP3, EGFR | 0.026511313 |
| GOTERM_BP_DIRECT | GO:0009409~response to cold | 5 | 0.01 | 0.001623365 | HSP90AA1, THRA, PPARG, ACADM, SOD2 | 0.026511313 |
| GOTERM_BP_DIRECT | GO:0060749~mammary gland alveolus development | 4 | 0.01 | 0.001650555 | AR, TPH1, ERBB4, ESR1 | 0.026774442 |
| GOTERM_BP_DIRECT | GO:0006935~chemotaxis | 8 | 0.02 | 0.001772551 | MAP2K1, PLAU, CCL5, RAC2, MAPK1, MAPK14, HRAS, TYMP | 0.028561697 |
| GOTERM_BP_DIRECT | GO:0034332~adherens junction organization | 5 | 0.01 | 0.001800127 | CDC42, SRC, CTNNA1, CSK, ANG | 0.02881395 |
| GOTERM_BP_DIRECT | GO:0035556~intracellular signal transduction | 15 | 0.04 | 0.001896097 | GSK3B, SYK, DAPK1, PDPK1, SRC, MAPK14, TGFBR1, ZAP70, CD209, AKT2, KIT, AKT1, HMOX1, JAK2, JAK3 | 0.030150444 |
| GOTERM_BP_DIRECT | GO:0033591~response to L-ascorbic acid | 3 | 0.01 | 0.001944885 | SPARC, GSTP1, SOD2 | 0.030280455 |
| GOTERM_BP_DIRECT | GO:1905007~positive regulation of epithelial to mesenchymal transition involved in endocardial cushion formation | 3 | 0.01 | 0.001944885 | TGFB2, TGFBR1, TGFBR2 | 0.030280455 |
| GOTERM_BP_DIRECT | GO:0060336~negative regulation of interferon-gamma-mediated signaling pathway | 3 | 0.01 | 0.001944885 | NR1H2, NR1H3, PPARG | 0.030280455 |
| GOTERM_BP_DIRECT | GO:0007597~blood coagulation, intrinsic pathway | 4 | 0.01 | 0.001960069 | F10, F11, GP1BA, F2 | 0.030280455 |
| GOTERM_BP_DIRECT | GO:0007179~transforming growth factor beta receptor signaling pathway | 7 | 0.02 | 0.001966914 | FKBP1A, TGFB2, PARP1, SRC, RHOA, TGFBR1, TGFBR2 | 0.030280455 |
| GOTERM_BP_DIRECT | GO:0001657~ureteric bud development | 5 | 0.01 | 0.001989798 | ARG2, RARB, BMP7, FGFR2, FGFR1 | 0.030438875 |
| GOTERM_BP_DIRECT | GO:0042632~cholesterol homeostasis | 6 | 0.02 | 0.002118318 | FABP3, FABP4, NR1H2, EPHX2, APOA2, NR1H3 | 0.032201095 |
| GOTERM_BP_DIRECT | GO:0008283~cell proliferation | 14 | 0.04 | 0.002234291 | TGFB2, SYK, SRC, IGF1, MIF, EGFR, AR, ERBB4, AKT1, HRAS, MET, CDK5R1, BCL2L1, PPARD | 0.033751756 |
| GOTERM_BP_DIRECT | GO:0060314~regulation of ryanodine-sensitive calcium-release channel activity | 4 | 0.01 | 0.002303393 | FKBP1A, PDE4D, CALM1, PRKACA | 0.034579514 |
| GOTERM_BP_DIRECT | GO:0030163~protein catabolic process | 5 | 0.01 | 0.002640965 | BACE1, AKT1, REN, CTSD, ELANE | 0.039402544 |
| GOTERM_BP_DIRECT | GO:0043524~negative regulation of neuron apoptotic process | 8 | 0.02 | 0.002769639 | GPI, ADORA2A, PDPK1, HMOX1, JAK2, SOD2, HRAS, BCL2L1 | 0.041068823 |
| GOTERM_BP_DIRECT | GO:0006469~negative regulation of protein kinase activity | 7 | 0.02 | 0.002848287 | RTN4R, FABP4, ADORA2A, PDPK1, GSTP1, AKT1, GP1BA | 0.041977499 |
| GOTERM_BP_DIRECT | GO:0007566~embryo implantation | 5 | 0.01 | 0.002886946 | RXRA, MMP2, MMP9, TGFBR2, PPARD | 0.042080315 |
| GOTERM_BP_DIRECT | GO:0060440~trachea formation | 3 | 0.01 | 0.002890084 | MAP2K1, MAPK1, TGFBR2 | 0.042080315 |
| GOTERM_BP_DIRECT | GO:0045861~negative regulation of proteolysis | 4 | 0.01 | 0.00309622 | NR1H2, NR1H3, AKT1, F2 | 0.044545026 |
| GOTERM_BP_DIRECT | GO:1900182~positive regulation of protein localization to nucleus | 4 | 0.01 | 0.00309622 | TGFB2, SRC, AKT1, F2 | 0.044545026 |
| GOTERM_BP_DIRECT | GO:0030855~epithelial cell differentiation | 6 | 0.02 | 0.003140075 | LGALS3, PPARG, BMP7, FGFR2, CTSB, CES1 | 0.044908643 |
| GOTERM_BP_DIRECT | GO:0071260~cellular response to mechanical stimulus | 6 | 0.02 | 0.003339681 | MAPK8, MMP7, CHEK1, CASP1, AKT1, EGFR | 0.04748241 |
| GOTERM_BP_DIRECT | GO:0023014~signal transduction by protein phosphorylation | 5 | 0.01 | 0.003425018 | TGFB2, INSR, KDR, TGFBR1, TGFBR2 | 0.048410923 |
| GOTERM_BP_DIRECT | GO:0070207~protein homotrimerization | 4 | 0.01 | 0.003547918 | ARG1, LCN2, MIF, OTC | 0.049288147 |
| GOTERM_BP_DIRECT | GO:0060045~positive regulation of cardiac muscle cell proliferation | 4 | 0.01 | 0.003547918 | ERBB4, MAPK14, FGFR2, FGFR1 | 0.049288147 |
| GOTERM_BP_DIRECT | GO:0016485~protein processing | 6 | 0.02 | 0.003548257 | F7, CASP7, CMA1, CASP1, CTSG, CTSS | 0.049288147 |
| GOTERM_BP_DIRECT | GO:0043065~positive regulation of apoptotic process | 12 | 0.03 | 0.003785442 | BMP2, MAPK8, ERBB4, SRC, ABL1, HMOX1, RARB, AKT1, BMP7, DUSP6, BCL2L1, TGM2 | 0.052282368 |
| GOTERM_BP_DIRECT | GO:0002223~stimulatory C-type lectin receptor signaling pathway | 7 | 0.02 | 0.003814514 | SYK, PDPK1, CD209, SRC, ICAM2, PRKACA, HRAS | 0.052384549 |
| GOTERM_BP_DIRECT | GO:0038095~Fc-epsilon receptor signaling pathway | 9 | 0.02 | 0.003888633 | MAPK10, MAPK8, SYK, PDPK1, MAPK1, GRB2, PIK3R1, CALM1, HRAS | 0.05310071 |
| GOTERM_BP_DIRECT | GO:0045822~negative regulation of heart contraction | 3 | 0.01 | 0.004008375 | PDE4D, JAK2, IL2 | 0.054219002 |
| GOTERM_BP_DIRECT | GO:0051591~response to cAMP | 5 | 0.01 | 0.004027512 | SPARC, STAT1, REN, TEK, AGXT | 0.054219002 |
| GOTERM_BP_DIRECT | GO:0003148~outflow tract septum morphogenesis | 4 | 0.01 | 0.004037824 | TGFB2, RARB, FGFR2, TGFBR2 | 0.054219002 |
| GOTERM_BP_DIRECT | GO:0051149~positive regulation of muscle cell differentiation | 4 | 0.01 | 0.004566868 | CDC42, CTNNA1, ABL1, MAPK14 | 0.060649005 |
| GOTERM_BP_DIRECT | GO:0048870~cell motility | 4 | 0.01 | 0.004566868 | MAP2K1, ADAM17, TGFBR1, EPHA2 | 0.060649005 |
| GOTERM_BP_DIRECT | GO:0021762~substantia nigra development | 5 | 0.01 | 0.005059539 | CDC42, G6PD, MAOB, CALM1, RHOA | 0.064960928 |
| GOTERM_BP_DIRECT | GO:0042752~regulation of circadian rhythm | 5 | 0.01 | 0.005059539 | MAPK10, MAPK8, NR1H3, PPARG, PPARA | 0.064960928 |
| GOTERM_BP_DIRECT | GO:0046686~response to cadmium ion | 4 | 0.01 | 0.005135918 | GPI, SPARC, ARG1, SOD2 | 0.064960928 |
| GOTERM_BP_DIRECT | GO:0070372~regulation of ERK1 and ERK2 cascade | 4 | 0.01 | 0.005135918 | SYK, GSTP1, FGFR2, EPHA2 | 0.064960928 |
| GOTERM_BP_DIRECT | GO:0019433~triglyceride catabolic process | 4 | 0.01 | 0.005135918 | FABP3, FABP4, FABP5, FABP7 | 0.064960928 |
| GOTERM_BP_DIRECT | GO:0031659~positive regulation of cyclin-dependent protein serine/threonine kinase activity involved in G1/S transition of mitotic cell cycle | 3 | 0.01 | 0.005294705 | ADAM17, AKT1, EGFR | 0.064960928 |
| GOTERM_BP_DIRECT | GO:0070723~response to cholesterol | 3 | 0.01 | 0.005294705 | F7, TGFBR1, TGFBR2 | 0.064960928 |
| GOTERM_BP_DIRECT | GO:0051597~response to methylmercury | 3 | 0.01 | 0.005294705 | ARSA, FECH, ARG1 | 0.064960928 |
| GOTERM_BP_DIRECT | GO:0002674~negative regulation of acute inflammatory response | 3 | 0.01 | 0.005294705 | APCS, GSTP1, PPARG | 0.064960928 |
| GOTERM_BP_DIRECT | GO:0042129~regulation of T cell proliferation | 3 | 0.01 | 0.005294705 | LGALS3, CD209, RAC2 | 0.064960928 |
| GOTERM_BP_DIRECT | GO:0010269~response to selenium ion | 3 | 0.01 | 0.005294705 | MAOB, ARG1, SOD2 | 0.064960928 |
| GOTERM_BP_DIRECT | GO:0006527~arginine catabolic process | 3 | 0.01 | 0.005294705 | NOS2, NOS3, ARG1 | 0.064960928 |
| GOTERM_BP_DIRECT | GO:2000641~regulation of early endosome to late endosome transport | 3 | 0.01 | 0.005294705 | MAP2K1, SRC, MAPK1 | 0.064960928 |
| GOTERM_BP_DIRECT | GO:0010518~positive regulation of phospholipase activity | 3 | 0.01 | 0.005294705 | PDPK1, FGFR2, FGFR1 | 0.064960928 |
| GOTERM_BP_DIRECT | GO:0032872~regulation of stress-activated MAPK cascade | 3 | 0.01 | 0.005294705 | MAP2K1, GSTP1, MAPK1 | 0.064960928 |
| GOTERM_BP_DIRECT | GO:0035264~multicellular organism growth | 6 | 0.02 | 0.0055663 | CDC42, AR, PDE4D, RARB, PTPN11, FGFR2 | 0.06794822 |
| GOTERM_BP_DIRECT | GO:0002053~positive regulation of mesenchymal cell proliferation | 4 | 0.01 | 0.005745781 | STAT1, FGFR2, FGFR1, TGFBR2 | 0.069437766 |
| GOTERM_BP_DIRECT | GO:0061621~canonical glycolysis | 4 | 0.01 | 0.005745781 | GPI, TPI1, GCK, HK1 | 0.069437766 |
| GOTERM_BP_DIRECT | GO:0042542~response to hydrogen peroxide | 5 | 0.01 | 0.005837885 | STAT1, SRC, CASP3, HMOX1, SOD2 | 0.070199845 |
| GOTERM_BP_DIRECT | GO:0045766~positive regulation of angiogenesis | 7 | 0.02 | 0.005936993 | NOS3, CMA1, KDR, HMOX1, TEK, PGF, TGFBR2 | 0.071038182 |
| GOTERM_BP_DIRECT | GO:0050731~positive regulation of peptidyl-tyrosine phosphorylation | 6 | 0.02 | 0.00617589 | SYK, SRC, ABL1, IGF1, MIF, JAK2 | 0.073532647 |
| GOTERM_BP_DIRECT | GO:0010800~positive regulation of peptidyl-threonine phosphorylation | 4 | 0.01 | 0.006397206 | GSK3B, MAPK1, CALM1, BMP7 | 0.075424622 |
| GOTERM_BP_DIRECT | GO:0014065~phosphatidylinositol 3-kinase signaling | 4 | 0.01 | 0.006397206 | IGF1, PIK3R1, PIK3CG, IGF1R | 0.075424622 |
| GOTERM_BP_DIRECT | GO:0006919~activation of cysteine-type endopeptidase activity involved in apoptotic process | 6 | 0.02 | 0.00649763 | LCK, CASP3, CASP1, PPARG, JAK2, S100A9 | 0.076236757 |
| GOTERM_BP_DIRECT | GO:0010863~positive regulation of phospholipase C activity | 3 | 0.01 | 0.006744126 | KIT, ESR1, FGFR1 | 0.0772538 |
| GOTERM_BP_DIRECT | GO:0007167~enzyme linked receptor protein signaling pathway | 3 | 0.01 | 0.006744126 | SYK, JAK2, JAK3 | 0.0772538 |
| GOTERM_BP_DIRECT | GO:0010907~positive regulation of glucose metabolic process | 3 | 0.01 | 0.006744126 | SRC, AKT2, AKT1 | 0.0772538 |
| GOTERM_BP_DIRECT | GO:0050778~positive regulation of immune response | 3 | 0.01 | 0.006744126 | TGFB2, CTSG, ELANE | 0.0772538 |
| GOTERM_BP_DIRECT | GO:0046638~positive regulation of alpha-beta T cell differentiation | 3 | 0.01 | 0.006744126 | ZAP70, PNP, SYK | 0.0772538 |
| GOTERM_BP_DIRECT | GO:0043388~positive regulation of DNA binding | 4 | 0.01 | 0.007090884 | IGF1, CALM1, JAK2, MMP9 | 0.080298651 |
| GOTERM_BP_DIRECT | GO:0032715~negative regulation of interleukin-6 production | 4 | 0.01 | 0.007090884 | GBA, NR1H4, CSK, BPI | 0.080298651 |
| GOTERM_BP_DIRECT | GO:0048511~rhythmic process | 5 | 0.01 | 0.007148731 | MAPK10, MAPK8, CSNK2A1, PPARG, CDK5R1 | 0.080298651 |
| GOTERM_BP_DIRECT | GO:0050853~B cell receptor signaling pathway | 5 | 0.01 | 0.007148731 | ZAP70, SYK, LCK, ABL1, MAPK1 | 0.080298651 |
| GOTERM_BP_DIRECT | GO:0050830~defense response to Gram-positive bacterium | 6 | 0.02 | 0.007176048 | ADAM17, PLA2G2A, ANG, RNASE3, LYZ, EPHA2 | 0.080298651 |
| GOTERM_BP_DIRECT | GO:0050680~negative regulation of epithelial cell proliferation | 5 | 0.01 | 0.008122502 | AR, TGFB2, CDK6, FGFR2, PPARD | 0.090470451 |
| GOTERM_BP_DIRECT | GO:0033160~positive regulation of protein import into nucleus, translocation | 3 | 0.01 | 0.008351794 | HSP90AA1, IGF1, JAK2 | 0.092174825 |
| GOTERM_BP_DIRECT | GO:0003149~membranous septum morphogenesis | 3 | 0.01 | 0.008351794 | TGFB2, FGFR2, TGFBR2 | 0.092174825 |
| GOTERM_BP_DIRECT | GO:0090004~positive regulation of establishment of protein localization to plasma membrane | 4 | 0.01 | 0.008607483 | PDPK1, AKT1, PIK3R1, EPHA2 | 0.093649619 |
| GOTERM_BP_DIRECT | GO:0030217~T cell differentiation | 4 | 0.01 | 0.008607483 | ZAP70, LCK, KIT, IL2 | 0.093649619 |
| GOTERM_BP_DIRECT | GO:0006730~one-carbon metabolic process | 4 | 0.01 | 0.008607483 | DHFR, CA1, AHCY, CA2 | 0.093649619 |
| GOTERM_BP_DIRECT | GO:0070301~cellular response to hydrogen peroxide | 5 | 0.01 | 0.008640408 | ARG1, MDM2, ABL1, LCN2, AKR1B1 | 0.093649619 |
| GOTERM_BP_DIRECT | GO:0070373~negative regulation of ERK1 and ERK2 cascade | 5 | 0.01 | 0.009179441 | PTPN1, GSTP1, ABL1, CSK, DUSP6 | 0.099047812 |
| GOTERM_BP_DIRECT | GO:0071407~cellular response to organic cyclic compound | 5 | 0.01 | 0.009739928 | BMP2, STAT1, CCL5, CASP3, AKT1 | 0.104628475 |
| GOTERM_BP_DIRECT | GO:0008152~metabolic process | 8 | 0.02 | 0.010033646 | ARSA, GSTM1, GSTO1, EPHX2, GSTP1, LYZ, ACAT1, CES1 | 0.10673823 |
| GOTERM_BP_DIRECT | GO:0017187~peptidyl-glutamic acid carboxylation | 3 | 0.01 | 0.010112973 | F7, F10, F2 | 0.10673823 |
| GOTERM_BP_DIRECT | GO:0002523~leukocyte migration involved in inflammatory response | 3 | 0.01 | 0.010112973 | SELE, S100A9, ELANE | 0.10673823 |
| GOTERM_BP_DIRECT | GO:0042770~signal transduction in response to DNA damage | 3 | 0.01 | 0.010112973 | ABL1, GRB2, MAPK14 | 0.10673823 |
| GOTERM_BP_DIRECT | GO:0001974~blood vessel remodeling | 4 | 0.01 | 0.010300032 | TGFB2, NOS3, MDM2, TGM2 | 0.107076041 |
| GOTERM_BP_DIRECT | GO:0003007~heart morphogenesis | 4 | 0.01 | 0.010300032 | FKBP1A, TGFB2, INSR, EPHB4 | 0.107076041 |
| GOTERM_BP_DIRECT | GO:0046677~response to antibiotic | 4 | 0.01 | 0.010300032 | HSP90AA1, CASP3, MDM2, JAK2 | 0.107076041 |
| GOTERM_BP_DIRECT | GO:0050679~positive regulation of epithelial cell proliferation | 5 | 0.01 | 0.010322184 | CCL5, IGF1, HRAS, EGFR, FGFR2 | 0.107076041 |
| GOTERM_BP_DIRECT | GO:0006950~response to stress | 5 | 0.01 | 0.010926512 | HSP90AA1, MAPK8, AKR1B1, MAPK1, EGFR | 0.112677421 |
| GOTERM_BP_DIRECT | GO:0019221~cytokine-mediated signaling pathway | 7 | 0.02 | 0.010955397 | RTN4R, FKBP1A, HCK, KIT, GP1BA, JAK2, TGM2 | 0.112677421 |
| GOTERM_BP_DIRECT | GO:0010634~positive regulation of epithelial cell migration | 4 | 0.01 | 0.011213457 | TGFB2, SRC, RAB11A, TGFBR2 | 0.112928857 |
| GOTERM_BP_DIRECT | GO:0043491~protein kinase B signaling | 4 | 0.01 | 0.011213457 | CCL5, AKT1, IGF1, EPHA2 | 0.112928857 |
| GOTERM_BP_DIRECT | GO:0002027~regulation of heart rate | 4 | 0.01 | 0.011213457 | PDE4D, MDM2, CALM1, PRKACA | 0.112928857 |
| GOTERM_BP_DIRECT | GO:0010718~positive regulation of epithelial to mesenchymal transition | 4 | 0.01 | 0.011213457 | TGFB2, BMP2, TGFBR1, TGFBR2 | 0.112928857 |
| GOTERM_BP_DIRECT | GO:0071364~cellular response to epidermal growth factor stimulus | 4 | 0.01 | 0.011213457 | PDPK1, GSTP1, AKT1, EGFR | 0.112928857 |
| GOTERM_BP_DIRECT | GO:0016525~negative regulation of angiogenesis | 5 | 0.01 | 0.01155321 | SPARC, STAT1, PDE3B, TEK, EPHA2 | 0.115867669 |
| GOTERM_BP_DIRECT | GO:0005975~carbohydrate metabolic process | 8 | 0.02 | 0.01201018 | CHIT1, GPI, ALDH2, GLO1, INSR, GBA, AKR1B1, ABO | 0.116238591 |
| GOTERM_BP_DIRECT | GO:0097067~cellular response to thyroid hormone stimulus | 3 | 0.01 | 0.012023024 | KIT, CTSS, CTSB | 0.116238591 |
| GOTERM_BP_DIRECT | GO:0031000~response to caffeine | 3 | 0.01 | 0.012023024 | FKBP1A, ADORA2A, PPARG | 0.116238591 |
| GOTERM_BP_DIRECT | GO:0071872~cellular response to epinephrine stimulus | 3 | 0.01 | 0.012023024 | PDE4D, PDE4B, PRKACA | 0.116238591 |
| GOTERM_BP_DIRECT | GO:0019430~removal of superoxide radicals | 3 | 0.01 | 0.012023024 | NQO1, NOS3, SOD2 | 0.116238591 |
| GOTERM_BP_DIRECT | GO:0000050~urea cycle | 3 | 0.01 | 0.012023024 | ARG2, ARG1, OTC | 0.116238591 |
| GOTERM_BP_DIRECT | GO:0045667~regulation of osteoblast differentiation | 3 | 0.01 | 0.012023024 | ESRRA, PRKACA, FGFR2 | 0.116238591 |
| GOTERM_BP_DIRECT | GO:0042738~exogenous drug catabolic process | 3 | 0.01 | 0.012023024 | CYP2C9, CYP2C8, NR1I2 | 0.116238591 |
| GOTERM_BP_DIRECT | GO:0060315~negative regulation of ryanodine-sensitive calcium-release channel activity | 3 | 0.01 | 0.012023024 | FKBP1A, GSTO1, CALM1 | 0.116238591 |
| GOTERM_BP_DIRECT | GO:0006096~glycolytic process | 4 | 0.01 | 0.012172178 | GPI, TPI1, GCK, HK1 | 0.117211772 |
| GOTERM_BP_DIRECT | GO:0030512~negative regulation of transforming growth factor beta receptor signaling pathway | 5 | 0.01 | 0.012874845 | ADAM17, PDPK1, TGFBR1, HSPA1A, TGFBR2 | 0.123486115 |
| GOTERM_BP_DIRECT | GO:0050918~positive chemotaxis | 4 | 0.01 | 0.013176535 | LGALS3, CCL5, MIF, MET | 0.125880179 |
| GOTERM_BP_DIRECT | GO:0033630~positive regulation of cell adhesion mediated by integrin | 3 | 0.01 | 0.01407741 | ZAP70, TGFB2, SYK | 0.131880229 |
| GOTERM_BP_DIRECT | GO:0060317~cardiac epithelial to mesenchymal transition | 3 | 0.01 | 0.01407741 | TGFB2, BMP2, TGFBR1 | 0.131880229 |
| GOTERM_BP_DIRECT | GO:0050435~beta-amyloid metabolic process | 3 | 0.01 | 0.01407741 | BACE1, MME, REN | 0.131880229 |
| GOTERM_BP_DIRECT | GO:0045780~positive regulation of bone resorption | 3 | 0.01 | 0.01407741 | SYK, CA2, EGFR | 0.131880229 |
| GOTERM_BP_DIRECT | GO:0060644~mammary gland epithelial cell differentiation | 3 | 0.01 | 0.01407741 | ERBB4, AKT2, AKT1 | 0.131880229 |
| GOTERM_BP_DIRECT | GO:0010043~response to zinc ion | 4 | 0.01 | 0.014226823 | CA2, ARG1, SOD2, OTC | 0.132765373 |
| GOTERM_BP_DIRECT | GO:0000122~negative regulation of transcription from RNA polymerase II promoter | 19 | 0.05 | 0.014307804 | ESRRA, PARP1, STAT1, VDR, NR1H2, NR1H4, NR1H3, ESR1, ESR2, BMP2, RXRA, MDM2, RARB, PPARG, PPARA, ELANE, FGFR2, FGFR1, PPARD | 0.133007548 |
| GOTERM_BP_DIRECT | GO:0032868~response to insulin | 5 | 0.01 | 0.015031885 | FABP3, PCK1, HADH, PPARA, OTC | 0.139203321 |
| GOTERM_BP_DIRECT | GO:0007050~cell cycle arrest | 7 | 0.02 | 0.015304257 | TGFB2, MAP2K1, CDK6, RHEB, ABL1, HRAS, TGFBR1 | 0.140822842 |
| GOTERM_BP_DIRECT | GO:2001240~negative regulation of extrinsic apoptotic signaling pathway in absence of ligand | 4 | 0.01 | 0.015323296 | CTNNA1, AKT1, BCL2L1, HSPA1A | 0.140822842 |
| GOTERM_BP_DIRECT | GO:0031103~axon regeneration | 3 | 0.01 | 0.016271691 | DHFR, CTNNA1, JAK2 | 0.146748798 |
| GOTERM_BP_DIRECT | GO:0001678~cellular glucose homeostasis | 3 | 0.01 | 0.016271691 | PIK3R1, GCK, HK1 | 0.146748798 |
| GOTERM_BP_DIRECT | GO:0060347~heart trabecula formation | 3 | 0.01 | 0.016271691 | FKBP1A, RBP4, TEK | 0.146748798 |
| GOTERM_BP_DIRECT | GO:0043254~regulation of protein complex assembly | 3 | 0.01 | 0.016271691 | HSPA8, HSP90AA1, PTPN11 | 0.146748798 |
| GOTERM_BP_DIRECT | GO:0030194~positive regulation of blood coagulation | 3 | 0.01 | 0.016271691 | F7, F2, S100A9 | 0.146748798 |
| GOTERM_BP_DIRECT | GO:0042157~lipoprotein metabolic process | 4 | 0.01 | 0.016466168 | ALB, APOA2, PPARA, PRKACA | 0.147950659 |
| GOTERM_BP_DIRECT | GO:0051091~positive regulation of sequence-specific DNA binding transcription factor activity | 6 | 0.02 | 0.016853508 | KIT, AKT1, PPARG, JAK2, ESR1, ESR2 | 0.150870106 |
| GOTERM_BP_DIRECT | GO:0098869~cellular oxidant detoxification | 5 | 0.01 | 0.017404267 | GSTO1, GSTP1, ALB, GSR, S100A9 | 0.15522551 |
| GOTERM_BP_DIRECT | GO:2000251~positive regulation of actin cytoskeleton reorganization | 3 | 0.01 | 0.018601525 | HCK, TEK, HRAS | 0.164087173 |
| GOTERM_BP_DIRECT | GO:0032270~positive regulation of cellular protein metabolic process | 3 | 0.01 | 0.018601525 | NR1H2, NR1H3, AKT1 | 0.164087173 |
| GOTERM_BP_DIRECT | GO:0006198~cAMP catabolic process | 3 | 0.01 | 0.018601525 | PDE4D, PDE3B, PDE4B | 0.164087173 |
| GOTERM_BP_DIRECT | GO:0030218~erythrocyte differentiation | 4 | 0.01 | 0.018891761 | THRA, CASP3, KIT, JAK2 | 0.165439809 |
| GOTERM_BP_DIRECT | GO:0051262~protein tetramerization | 4 | 0.01 | 0.018891761 | CCL5, SHMT1, HMGCR, IGF1R | 0.165439809 |
| GOTERM_BP_DIRECT | GO:0032259~methylation | 5 | 0.01 | 0.019997809 | PNMT, MTAP, AHCY, GSTO1, HNMT | 0.173702024 |
| GOTERM_BP_DIRECT | GO:0048839~inner ear development | 4 | 0.01 | 0.020174719 | TGFB2, BMP2, SPARC, PTPN11 | 0.173702024 |
| GOTERM_BP_DIRECT | GO:0044255~cellular lipid metabolic process | 4 | 0.01 | 0.020174719 | RXRA, NR1H2, NR1H3, PPARA | 0.173702024 |
| GOTERM_BP_DIRECT | GO:0032024~positive regulation of insulin secretion | 4 | 0.01 | 0.020174719 | RBP4, JAK2, GCK, PPARD | 0.173702024 |
| GOTERM_BP_DIRECT | GO:0071356~cellular response to tumor necrosis factor | 6 | 0.02 | 0.020194567 | FABP4, CCL5, GBA, LCN2, RORA, PCK1 | 0.173702024 |
| GOTERM_BP_DIRECT | GO:0001889~liver development | 5 | 0.01 | 0.020912357 | ARG1, ACADM, SOD2, OTC, ACAT1 | 0.176765453 |
| GOTERM_BP_DIRECT | GO:0010613~positive regulation of cardiac muscle hypertrophy | 3 | 0.01 | 0.021062661 | PARP1, PDE5A, IGF1 | 0.176765453 |
| GOTERM_BP_DIRECT | GO:0003203~endocardial cushion morphogenesis | 3 | 0.01 | 0.021062661 | TGFB2, BMP2, MDM2 | 0.176765453 |
| GOTERM_BP_DIRECT | GO:0048011~neurotrophin TRK receptor signaling pathway | 3 | 0.01 | 0.021062661 | SRC, CASP3, PTPN11 | 0.176765453 |
| GOTERM_BP_DIRECT | GO:0035162~embryonic hemopoiesis | 3 | 0.01 | 0.021062661 | KIT, KDR, TGFBR2 | 0.176765453 |
| GOTERM_BP_DIRECT | GO:0031396~regulation of protein ubiquitination | 3 | 0.01 | 0.021062661 | HSP90AA1, TGFBR1, HSPA1A | 0.176765453 |
| GOTERM_BP_DIRECT | GO:0097194~execution phase of apoptosis | 3 | 0.01 | 0.021062661 | CASP7, CASP3, AKT1 | 0.176765453 |
| GOTERM_BP_DIRECT | GO:0001541~ovarian follicle development | 4 | 0.01 | 0.021504548 | KIT, CTNNA1, ANG, BCL2L1 | 0.179849453 |
| GOTERM_BP_DIRECT | GO:1900034~regulation of cellular response to heat | 5 | 0.01 | 0.021852207 | HSPA8, GSK3B, HSP90AA1, MAPK1, HSPA1A | 0.18150098 |
| GOTERM_BP_DIRECT | GO:0007623~circadian rhythm | 5 | 0.01 | 0.021852207 | GSK3B, F7, TPH1, NOS2, EGFR | 0.18150098 |
| GOTERM_BP_DIRECT | GO:0014823~response to activity | 4 | 0.01 | 0.022881278 | PCK1, SOD2, HADH, PPARD | 0.188109007 |
| GOTERM_BP_DIRECT | GO:0008202~steroid metabolic process | 4 | 0.01 | 0.022881278 | CYP2C9, CYP2C8, SULT1E1, NR1I2 | 0.188109007 |
| GOTERM_BP_DIRECT | GO:0048538~thymus development | 4 | 0.01 | 0.022881278 | MAP2K1, ABL1, MAPK1, TGFBR1 | 0.188109007 |
| GOTERM_BP_DIRECT | GO:0090201~negative regulation of release of cytochrome c from mitochondria | 3 | 0.01 | 0.023650941 | AKT1, IGF1, BCL2L1 | 0.19312272 |
| GOTERM_BP_DIRECT | GO:1901216~positive regulation of neuron death | 3 | 0.01 | 0.023650941 | GSK3B, PARP1, ABL1 | 0.19312272 |
| GOTERM_BP_DIRECT | GO:0007219~Notch signaling pathway | 6 | 0.02 | 0.023942298 | ADAM17, BMP2, CDK6, NR1H4, AGXT, TGFBR2 | 0.194706765 |
| GOTERM_BP_DIRECT | GO:0043434~response to peptide hormone | 4 | 0.01 | 0.024304906 | SPARC, STAT1, TEK, BMP7 | 0.194706765 |
| GOTERM_BP_DIRECT | GO:0006635~fatty acid beta-oxidation | 4 | 0.01 | 0.024304906 | ACADM, HADH, ACAT1, PPARD | 0.194706765 |
| GOTERM_BP_DIRECT | GO:0006094~gluconeogenesis | 4 | 0.01 | 0.024304906 | GPI, RBP4, TPI1, PCK1 | 0.194706765 |
| GOTERM_BP_DIRECT | GO:0016241~regulation of macroautophagy | 4 | 0.01 | 0.024304906 | MAPK8, CASP3, GBA, CDK5R1 | 0.194706765 |
| GOTERM_BP_DIRECT | GO:0007264~small GTPase mediated signal transduction | 9 | 0.02 | 0.024328276 | CDC42, ARF1, RHEB, RAC2, HMOX1, HRAS, RAB5A, RHOA, RAB11A | 0.194706765 |
| GOTERM_BP_DIRECT | GO:0045666~positive regulation of neuron differentiation | 5 | 0.01 | 0.024825068 | BMP2, RARB, BMP7, RHOA, FGFR1 | 0.198027027 |
| GOTERM_BP_DIRECT | GO:0045892~negative regulation of transcription, DNA-templated | 14 | 0.04 | 0.025930095 | HSPA8, THRA, SRC, VDR, NR1H2, NR1I3, NR1I2, BMP7, BMP2, FABP4, MDM2, PPARG, CDK5R1, PPARD | 0.206161314 |
| GOTERM_BP_DIRECT | GO:0033189~response to vitamin A | 3 | 0.01 | 0.0263623 | ARG1, PPARG, PPARD | 0.206875584 |
| GOTERM_BP_DIRECT | GO:0019373~epoxygenase P450 pathway | 3 | 0.01 | 0.0263623 | CYP2C9, CYP2C8, EPHX2 | 0.206875584 |
| GOTERM_BP_DIRECT | GO:0030225~macrophage differentiation | 3 | 0.01 | 0.0263623 | CDC42, PARP1, MMP9 | 0.206875584 |
| GOTERM_BP_DIRECT | GO:0010880~regulation of release of sequestered calcium ion into cytosol by sarcoplasmic reticulum | 3 | 0.01 | 0.0263623 | GSTO1, PDE4D, CALM1 | 0.206875584 |
| GOTERM_BP_DIRECT | GO:0001503~ossification | 5 | 0.01 | 0.026936065 | SPARC, THRA, MMP9, BMP7, EGFR | 0.210014418 |
| GOTERM_BP_DIRECT | GO:0046718~viral entry into host cell | 5 | 0.01 | 0.026936065 | DPP4, ACE2, CD209, CTSB, HSPA1A | 0.210014418 |
| GOTERM_BP_DIRECT | GO:0045165~cell fate commitment | 4 | 0.01 | 0.027292695 | BMP2, ERBB4, PPARG, FGFR2 | 0.211430907 |
| GOTERM_BP_DIRECT | GO:0032729~positive regulation of interferon-gamma production | 4 | 0.01 | 0.027292695 | PDE4D, PDE4B, HRAS, IL2 | 0.211430907 |
| GOTERM_BP_DIRECT | GO:0044209~AMP salvage | 2 | 0.01 | 0.028146778 | ADK, APRT | 0.21326258 |
| GOTERM_BP_DIRECT | GO:0038183~bile acid signaling pathway | 2 | 0.01 | 0.028146778 | VDR, NR1H4 | 0.21326258 |
| GOTERM_BP_DIRECT | GO:0035607~fibroblast growth factor receptor signaling pathway involved in orbitofrontal cortex development | 2 | 0.01 | 0.028146778 | FGFR2, FGFR1 | 0.21326258 |
| GOTERM_BP_DIRECT | GO:0006738~nicotinamide riboside catabolic process | 2 | 0.01 | 0.028146778 | MTAP, PNP | 0.21326258 |
| GOTERM_BP_DIRECT | GO:0043366~beta selection | 2 | 0.01 | 0.028146778 | ZAP70, SYK | 0.21326258 |
| GOTERM_BP_DIRECT | GO:0014806~smooth muscle hyperplasia | 2 | 0.01 | 0.028146778 | NOS3, HMOX1 | 0.21326258 |
| GOTERM_BP_DIRECT | GO:1901898~negative regulation of relaxation of cardiac muscle | 2 | 0.01 | 0.028146778 | PDE4D, PDE4B | 0.21326258 |
| GOTERM_BP_DIRECT | GO:0032956~regulation of actin cytoskeleton organization | 4 | 0.01 | 0.028856697 | TGFB2, ABL1, RHOA, CDK5R1 | 0.215776455 |
| GOTERM_BP_DIRECT | GO:0031532~actin cytoskeleton reorganization | 4 | 0.01 | 0.028856697 | PTPN1, KIT, S100A9, RHOA | 0.215776455 |
| GOTERM_BP_DIRECT | GO:0042110~T cell activation | 4 | 0.01 | 0.028856697 | FKBP1A, DPP4, ZAP70, PIK3CG | 0.215776455 |
| GOTERM_BP_DIRECT | GO:0008543~fibroblast growth factor receptor signaling pathway | 5 | 0.01 | 0.029151395 | MAPK1, GRB2, PTPN11, FGFR2, FGFR1 | 0.215776455 |
| GOTERM_BP_DIRECT | GO:0010881~regulation of cardiac muscle contraction by regulation of the release of sequestered calcium ion | 3 | 0.01 | 0.02919276 | GSTO1, CALM1, PRKACA | 0.215776455 |
| GOTERM_BP_DIRECT | GO:0046827~positive regulation of protein export from nucleus | 3 | 0.01 | 0.02919276 | GSK3B, MDM2, PRKACA | 0.215776455 |
| GOTERM_BP_DIRECT | GO:0043393~regulation of protein binding | 3 | 0.01 | 0.02919276 | SRC, PRKACA, TGFBR1 | 0.215776455 |
| GOTERM_BP_DIRECT | GO:0009116~nucleoside metabolic process | 3 | 0.01 | 0.02919276 | MTAP, PNP, APRT | 0.215776455 |
| GOTERM_BP_DIRECT | GO:0010862~positive regulation of pathway-restricted SMAD protein phosphorylation | 4 | 0.01 | 0.030467285 | TGFB2, BMP2, BMP7, TGFBR1 | 0.223149783 |
| GOTERM_BP_DIRECT | GO:0009408~response to heat | 4 | 0.01 | 0.030467285 | HSP90AA1, NOS3, AKT1, IGF1 | 0.223149783 |
| GOTERM_BP_DIRECT | GO:0006909~phagocytosis | 4 | 0.01 | 0.030467285 | ADORA2A, ITGAL, RAB5A, ELANE | 0.223149783 |
| GOTERM_BP_DIRECT | GO:0043547~positive regulation of GTPase activity | 15 | 0.04 | 0.03096338 | RTN4R, GSK3B, EGFR, IL2, ERBB4, CCL5, KIT, GRB2, TEK, CALM1, JAK2, HRAS, JAK3, FGFR2, FGFR1 | 0.226098158 |
| GOTERM_BP_DIRECT | GO:1901796~regulation of signal transduction by p53 class mediator | 6 | 0.02 | 0.031759734 | CSNK2A1, CHEK1, MDM2, AKT1, MAPK14, CDK5R1 | 0.229140367 |
| GOTERM_BP_DIRECT | GO:0042981~regulation of apoptotic process | 8 | 0.02 | 0.032015847 | STAT1, DAPK1, CASP1, JAK2, ESR1, BMP7, CTSB, BCL2L1 | 0.229140367 |
| GOTERM_BP_DIRECT | GO:0050850~positive regulation of calcium-mediated signaling | 3 | 0.01 | 0.03213843 | ZAP70, HINT1, SYK | 0.229140367 |
| GOTERM_BP_DIRECT | GO:0048545~response to steroid hormone | 3 | 0.01 | 0.03213843 | CA2, MDM2, TGFBR2 | 0.229140367 |
| GOTERM_BP_DIRECT | GO:0046697~decidualization | 3 | 0.01 | 0.03213843 | VDR, CTSB, PPARD | 0.229140367 |
| GOTERM_BP_DIRECT | GO:0071375~cellular response to peptide hormone stimulus | 3 | 0.01 | 0.03213843 | SRC, MDM2, CSK | 0.229140367 |
| GOTERM_BP_DIRECT | GO:0035584~calcium-mediated signaling using intracellular calcium source | 3 | 0.01 | 0.03213843 | SELP, KDR, PRKACA | 0.229140367 |
| GOTERM_BP_DIRECT | GO:0014911~positive regulation of smooth muscle cell migration | 3 | 0.01 | 0.03213843 | SRC, CCL5, IGF1 | 0.229140367 |
| GOTERM_BP_DIRECT | GO:0010506~regulation of autophagy | 4 | 0.01 | 0.033827604 | DAPK1, PSAP, CASP1, ABL1 | 0.238866196 |
| GOTERM_BP_DIRECT | GO:0007157~heterophilic cell-cell adhesion via plasma membrane cell adhesion molecules | 4 | 0.01 | 0.033827604 | SELP, CD209, ITGAL, SELE | 0.238866196 |
| GOTERM_BP_DIRECT | GO:0006928~movement of cell or subcellular component | 5 | 0.01 | 0.033897851 | MAP2K1, IGF1, ITGAL, JAK2, MAPK14 | 0.238866196 |
| GOTERM_BP_DIRECT | GO:0001822~kidney development | 5 | 0.01 | 0.033897851 | TGFB2, MME, CA2, REN, TGFBR1 | 0.238866196 |
| GOTERM_BP_DIRECT | GO:0042730~fibrinolysis | 3 | 0.01 | 0.035195504 | PLAU, GP1BA, F2 | 0.246572562 |
| GOTERM_BP_DIRECT | GO:0030520~intracellular estrogen receptor signaling pathway | 3 | 0.01 | 0.035195504 | SRC, ESR1, ESR2 | 0.246572562 |
| GOTERM_BP_DIRECT | GO:2001243~negative regulation of intrinsic apoptotic signaling pathway | 3 | 0.01 | 0.038360263 | SRC, MMP9, BCL2L1 | 0.265664056 |
| GOTERM_BP_DIRECT | GO:0046427~positive regulation of JAK-STAT cascade | 3 | 0.01 | 0.038360263 | CCL5, KIT, AKR1B1 | 0.265664056 |
| GOTERM_BP_DIRECT | GO:0001502~cartilage condensation | 3 | 0.01 | 0.038360263 | TGFB2, THRA, MAPK14 | 0.265664056 |
| GOTERM_BP_DIRECT | GO:1901687~glutathione derivative biosynthetic process | 3 | 0.01 | 0.038360263 | GSTM1, GSTO1, GSTP1 | 0.265664056 |
| GOTERM_BP_DIRECT | GO:0030316~osteoclast differentiation | 3 | 0.01 | 0.041629067 | GLO1, MAPK14, EPHA2 | 0.272386147 |
| GOTERM_BP_DIRECT | GO:0009880~embryonic pattern specification | 3 | 0.01 | 0.041629067 | ERBB4, BMP7, FGFR2 | 0.272386147 |
| GOTERM_BP_DIRECT | GO:0035924~cellular response to vascular endothelial growth factor stimulus | 3 | 0.01 | 0.041629067 | KDR, AKT1, MAPK14 | 0.272386147 |
| GOTERM_BP_DIRECT | GO:0016239~positive regulation of macroautophagy | 3 | 0.01 | 0.041629067 | GBA, KDR, HMOX1 | 0.272386147 |
| GOTERM_BP_DIRECT | GO:0046835~carbohydrate phosphorylation | 3 | 0.01 | 0.041629067 | ADK, GCK, HK1 | 0.272386147 |
| GOTERM_BP_DIRECT | GO:0009887~organ morphogenesis | 5 | 0.01 | 0.041813617 | GSK3B, BMP2, SYK, HRAS, FGFR2 | 0.272386147 |
| GOTERM_BP_DIRECT | GO:0060745~mammary gland branching involved in pregnancy | 2 | 0.01 | 0.041922899 | VDR, ESR1 | 0.272386147 |
| GOTERM_BP_DIRECT | GO:0003274~endocardial cushion fusion | 2 | 0.01 | 0.041922899 | TGFB2, TGFBR2 | 0.272386147 |
| GOTERM_BP_DIRECT | GO:0072136~metanephric mesenchymal cell proliferation involved in metanephros development | 2 | 0.01 | 0.041922899 | STAT1, BMP7 | 0.272386147 |
| GOTERM_BP_DIRECT | GO:0051791~medium-chain fatty acid metabolic process | 2 | 0.01 | 0.041922899 | ACADM, CES1 | 0.272386147 |
| GOTERM_BP_DIRECT | GO:0008295~spermidine biosynthetic process | 2 | 0.01 | 0.041922899 | AMD1, SRM | 0.272386147 |
| GOTERM_BP_DIRECT | GO:0019254~carnitine metabolic process, CoA-linked | 2 | 0.01 | 0.041922899 | ACADM, CRAT | 0.272386147 |
| GOTERM_BP_DIRECT | GO:0061684~chaperone-mediated autophagy | 2 | 0.01 | 0.041922899 | HSPA8, HSP90AA1 | 0.272386147 |
| GOTERM_BP_DIRECT | GO:0060688~regulation of morphogenesis of a branching structure | 2 | 0.01 | 0.041922899 | FGFR2, PGF | 0.272386147 |
| GOTERM_BP_DIRECT | GO:0010871~negative regulation of receptor biosynthetic process | 2 | 0.01 | 0.041922899 | PPARG, PPARA | 0.272386147 |
| GOTERM_BP_DIRECT | GO:0090135~actin filament branching | 2 | 0.01 | 0.041922899 | CDC42, ABL1 | 0.272386147 |
| GOTERM_BP_DIRECT | GO:0021697~cerebellar cortex formation | 2 | 0.01 | 0.041922899 | MAP2K1, PTPN11 | 0.272386147 |
| GOTERM_BP_DIRECT | GO:0010641~positive regulation of platelet-derived growth factor receptor signaling pathway | 2 | 0.01 | 0.041922899 | F7, SRC | 0.272386147 |
| GOTERM_BP_DIRECT | GO:0031281~positive regulation of cyclase activity | 2 | 0.01 | 0.041922899 | MAPK8, MAPK14 | 0.272386147 |
| GOTERM_BP_DIRECT | GO:0060523~prostate epithelial cord elongation | 2 | 0.01 | 0.041922899 | ESR1, FGFR2 | 0.272386147 |
| GOTERM_BP_DIRECT | GO:0021847~ventricular zone neuroblast division | 2 | 0.01 | 0.041922899 | FGFR2, FGFR1 | 0.272386147 |
| GOTERM_BP_DIRECT | GO:0070141~response to UV-A | 2 | 0.01 | 0.041922899 | AKT1, EGFR | 0.272386147 |
| GOTERM_BP_DIRECT | GO:0045726~positive regulation of integrin biosynthetic process | 2 | 0.01 | 0.041922899 | AR, TGFB2 | 0.272386147 |
| GOTERM_BP_DIRECT | GO:0042475~odontogenesis of dentin-containing tooth | 4 | 0.01 | 0.043030268 | BMP2, CA2, CTNNA1, BMP7 | 0.278085983 |
| GOTERM_BP_DIRECT | GO:0050829~defense response to Gram-negative bacterium | 4 | 0.01 | 0.043030268 | SELP, NOS2, BPI, LYZ | 0.278085983 |
| GOTERM_BP_DIRECT | GO:0070371~ERK1 and ERK2 cascade | 3 | 0.01 | 0.044998357 | MAP2K1, MAPK1, IGF1 | 0.290029413 |
| GOTERM_BP_DIRECT | GO:0006897~endocytosis | 6 | 0.02 | 0.047996532 | CDC42, CD209, HRAS, RAB5A, PIK3CG, BCL2L1 | 0.304257337 |
| GOTERM_BP_DIRECT | GO:0071158~positive regulation of cell cycle arrest | 3 | 0.01 | 0.048464657 | FAP, PRKACA, CDK5R1 | 0.304257337 |
| GOTERM_BP_DIRECT | GO:0050715~positive regulation of cytokine secretion | 3 | 0.01 | 0.048464657 | SYK, SRC, MIF | 0.304257337 |
| GOTERM_BP_DIRECT | GO:0002040~sprouting angiogenesis | 3 | 0.01 | 0.048464657 | CDC42, TEK, PGF | 0.304257337 |
| GOTERM_BP_DIRECT | GO:0060135~maternal process involved in female pregnancy | 3 | 0.01 | 0.048464657 | MMP7, ARG1, AKR1B1 | 0.304257337 |
| GOTERM_BP_DIRECT | GO:0034612~response to tumor necrosis factor | 3 | 0.01 | 0.048464657 | CASP3, JAK2, SELE | 0.304257337 |
| GOTERM_BP_DIRECT | GO:0006465~signal peptide processing | 3 | 0.01 | 0.048464657 | F7, F10, F2 | 0.304257337 |
| GOTERM_BP_DIRECT | GO:0001816~cytokine production | 3 | 0.01 | 0.048464657 | FABP4, S100A9, PIK3CG | 0.304257337 |
| GOTERM_BP_DIRECT | GO:0030878~thyroid gland development | 3 | 0.01 | 0.048464657 | MAP2K1, THRA, MAPK1 | 0.304257337 |
| GOTERM_BP_DIRECT | GO:0055010~ventricular cardiac muscle tissue morphogenesis | 3 | 0.01 | 0.048464657 | FKBP1A, RXRA, FGFR2 | 0.304257337 |
| GOTERM_BP_DIRECT | GO:0006661~phosphatidylinositol biosynthetic process | 4 | 0.01 | 0.049090885 | ARF1, IMPA1, PIK3R1, PIK3CG | 0.307390333 |
| GOTERM_BP_DIRECT | GO:0030097~hemopoiesis | 4 | 0.01 | 0.051198972 | TGFB2, LCK, KIT, SOD2 | 0.315142562 |
| GOTERM_BP_DIRECT | GO:0009612~response to mechanical stimulus | 4 | 0.01 | 0.051198972 | STAT1, SRC, PPARG, TGFBR2 | 0.315142562 |
| GOTERM_BP_DIRECT | GO:0060338~regulation of type I interferon-mediated signaling pathway | 3 | 0.01 | 0.052024563 | PTPN1, STAT1, PTPN11 | 0.315142562 |
| GOTERM_BP_DIRECT | GO:0040014~regulation of multicellular organism growth | 3 | 0.01 | 0.052024563 | PTPN11, IGF1, FGFR2 | 0.315142562 |
| GOTERM_BP_DIRECT | GO:0030041~actin filament polymerization | 3 | 0.01 | 0.052024563 | ABL1, ANG, JAK2 | 0.315142562 |
| GOTERM_BP_DIRECT | GO:0005978~glycogen biosynthetic process | 3 | 0.01 | 0.052024563 | AKT2, AKT1, ACADM | 0.315142562 |
| GOTERM_BP_DIRECT | GO:0007202~activation of phospholipase C activity | 3 | 0.01 | 0.052024563 | ANG, SELE, EGFR | 0.315142562 |
| GOTERM_BP_DIRECT | GO:0007229~integrin-mediated signaling pathway | 5 | 0.01 | 0.05225943 | HCK, SYK, SRC, PTPN11, ITGAL | 0.315142562 |
| GOTERM_BP_DIRECT | GO:0010468~regulation of gene expression | 5 | 0.01 | 0.053857669 | CDK6, IGF1, F2, TGFBR1, TGFBR2 | 0.315142562 |
| GOTERM_BP_DIRECT | GO:0007258~JUN phosphorylation | 2 | 0.01 | 0.055504551 | MAPK10, MAPK8 | 0.315142562 |
| GOTERM_BP_DIRECT | GO:0015936~coenzyme A metabolic process | 2 | 0.01 | 0.055504551 | HMGCR, ACAT1 | 0.315142562 |
| GOTERM_BP_DIRECT | GO:0006166~purine ribonucleoside salvage | 2 | 0.01 | 0.055504551 | MTAP, ADK | 0.315142562 |
| GOTERM_BP_DIRECT | GO:0010664~negative regulation of striated muscle cell apoptotic process | 2 | 0.01 | 0.055504551 | HMGCR, BMP7 | 0.315142562 |
| GOTERM_BP_DIRECT | GO:0060485~mesenchyme development | 2 | 0.01 | 0.055504551 | BMP2, BMP7 | 0.315142562 |
| GOTERM_BP_DIRECT | GO:0032376~positive regulation of cholesterol transport | 2 | 0.01 | 0.055504551 | NR1H2, NR1H3 | 0.315142562 |
| GOTERM_BP_DIRECT | GO:0019448~L-cysteine catabolic process | 2 | 0.01 | 0.055504551 | CBS, AGXT | 0.315142562 |
| GOTERM_BP_DIRECT | GO:0018119~peptidyl-cysteine S-nitrosylation | 2 | 0.01 | 0.055504551 | NOS2, ADH5 | 0.315142562 |
| GOTERM_BP_DIRECT | GO:0060571~morphogenesis of an epithelial fold | 2 | 0.01 | 0.055504551 | AR, EGFR | 0.315142562 |
| GOTERM_BP_DIRECT | GO:0070232~regulation of T cell apoptotic process | 2 | 0.01 | 0.055504551 | LGALS3, JAK3 | 0.315142562 |
| GOTERM_BP_DIRECT | GO:0002283~neutrophil activation involved in immune response | 2 | 0.01 | 0.055504551 | ZAP70, SYK | 0.315142562 |
| GOTERM_BP_DIRECT | GO:0055118~negative regulation of cardiac muscle contraction | 2 | 0.01 | 0.055504551 | PDE5A, PIK3CG | 0.315142562 |
| GOTERM_BP_DIRECT | GO:0038127~ERBB signaling pathway | 2 | 0.01 | 0.055504551 | MAPK1, PTPN11 | 0.315142562 |
| GOTERM_BP_DIRECT | GO:0051409~response to nitrosative stress | 2 | 0.01 | 0.055504551 | ADH5, DUSP6 | 0.315142562 |
| GOTERM_BP_DIRECT | GO:1903800~positive regulation of production of miRNAs involved in gene silencing by miRNA | 2 | 0.01 | 0.055504551 | MAP2K1, EGFR | 0.315142562 |
| GOTERM_BP_DIRECT | GO:0048550~negative regulation of pinocytosis | 2 | 0.01 | 0.055504551 | NR1H2, NR1H3 | 0.315142562 |
| GOTERM_BP_DIRECT | GO:0010716~negative regulation of extracellular matrix disassembly | 2 | 0.01 | 0.055504551 | DPP4, FAP | 0.315142562 |
| GOTERM_BP_DIRECT | GO:0032369~negative regulation of lipid transport | 2 | 0.01 | 0.055504551 | NR1H2, NR1H3 | 0.315142562 |
| GOTERM_BP_DIRECT | GO:0051882~mitochondrial depolarization | 2 | 0.01 | 0.055504551 | CASP1, ABL1 | 0.315142562 |
| GOTERM_BP_DIRECT | GO:1900015~regulation of cytokine production involved in inflammatory response | 2 | 0.01 | 0.055504551 | NOS2, MAPK14 | 0.315142562 |
| GOTERM_BP_DIRECT | GO:0010748~negative regulation of plasma membrane long-chain fatty acid transport | 2 | 0.01 | 0.055504551 | AKT2, AKT1 | 0.315142562 |
| GOTERM_BP_DIRECT | GO:0060742~epithelial cell differentiation involved in prostate gland development | 2 | 0.01 | 0.055504551 | AR, PSAP | 0.315142562 |
| GOTERM_BP_DIRECT | GO:0090170~regulation of Golgi inheritance | 2 | 0.01 | 0.055504551 | MAP2K1, MAPK1 | 0.315142562 |
| GOTERM_BP_DIRECT | GO:0043415~positive regulation of skeletal muscle tissue regeneration | 2 | 0.01 | 0.055504551 | TGFBR2, PPARD | 0.315142562 |
| GOTERM_BP_DIRECT | GO:0006565~L-serine catabolic process | 2 | 0.01 | 0.055504551 | CBS, SHMT1 | 0.315142562 |
| GOTERM_BP_DIRECT | GO:0007260~tyrosine phosphorylation of STAT protein | 2 | 0.01 | 0.055504551 | JAK2, JAK3 | 0.315142562 |
| GOTERM_BP_DIRECT | GO:0001523~retinoid metabolic process | 4 | 0.01 | 0.05554472 | RBP4, TTR, APOA2, ADH5 | 0.315142562 |
| GOTERM_BP_DIRECT | GO:0015721~bile acid and bile salt transport | 3 | 0.01 | 0.055674751 | RXRA, ALB, NR1H4 | 0.315142562 |
| GOTERM_BP_DIRECT | GO:0031069~hair follicle morphogenesis | 3 | 0.01 | 0.055674751 | CDC42, TGFB2, FGFR2 | 0.315142562 |
| GOTERM_BP_DIRECT | GO:0017144~drug metabolic process | 3 | 0.01 | 0.055674751 | CYP2C9, CYP2C8, EPHX2 | 0.315142562 |
| GOTERM_BP_DIRECT | GO:0051281~positive regulation of release of sequestered calcium ion into cytosol | 3 | 0.01 | 0.055674751 | PDPK1, ABL1, F2 | 0.315142562 |
| GOTERM_BP_DIRECT | GO:0035987~endodermal cell differentiation | 3 | 0.01 | 0.055674751 | MMP2, MMP8, MMP9 | 0.315142562 |
| GOTERM_BP_DIRECT | GO:0000902~cell morphogenesis | 4 | 0.01 | 0.057781576 | TGFB2, VDR, GP1BA, MAPK14 | 0.326303901 |
| GOTERM_BP_DIRECT | GO:0009755~hormone-mediated signaling pathway | 3 | 0.01 | 0.059411972 | THRA, REN, PTPN11 | 0.330112037 |
| GOTERM_BP_DIRECT | GO:0045931~positive regulation of mitotic cell cycle | 3 | 0.01 | 0.059411972 | MDM2, ABL1, PTPN11 | 0.330112037 |
| GOTERM_BP_DIRECT | GO:0051881~regulation of mitochondrial membrane potential | 3 | 0.01 | 0.059411972 | ADORA2A, SOD2, BCL2L1 | 0.330112037 |
| GOTERM_BP_DIRECT | GO:2000352~negative regulation of endothelial cell apoptotic process | 3 | 0.01 | 0.059411972 | ABL1, KDR, TEK | 0.330112037 |
| GOTERM_BP_DIRECT | GO:0050690~regulation of defense response to virus by virus | 3 | 0.01 | 0.059411972 | HCK, ARF1, LCK | 0.330112037 |
| GOTERM_BP_DIRECT | GO:0040007~growth | 3 | 0.01 | 0.059411972 | BMP2, BMP7, BCL2L1 | 0.330112037 |
| GOTERM_BP_DIRECT | GO:0001707~mesoderm formation | 3 | 0.01 | 0.059411972 | GPI, PRKACA, BMP7 | 0.330112037 |
| GOTERM_BP_DIRECT | GO:0009611~response to wounding | 4 | 0.01 | 0.060060527 | TGFB2, NMNAT1, FABP5, F2 | 0.332950214 |
| GOTERM_BP_DIRECT | GO:0034599~cellular response to oxidative stress | 4 | 0.01 | 0.062381134 | G6PD, PARP1, ABL1, HSPA1A | 0.344235619 |
| GOTERM_BP_DIRECT | GO:0032436~positive regulation of proteasomal ubiquitin-dependent protein catabolic process | 4 | 0.01 | 0.062381134 | GSK3B, MDM2, AKT1, HSPA1A | 0.344235619 |
| GOTERM_BP_DIRECT | GO:0071549~cellular response to dexamethasone stimulus | 3 | 0.01 | 0.063233048 | FECH, ARG1, EGFR | 0.345778906 |
| GOTERM_BP_DIRECT | GO:0060412~ventricular septum morphogenesis | 3 | 0.01 | 0.063233048 | TGFB2, TGFBR1, TGFBR2 | 0.345778906 |
| GOTERM_BP_DIRECT | GO:0048008~platelet-derived growth factor receptor signaling pathway | 3 | 0.01 | 0.063233048 | SRC, PTPN11, JAK2 | 0.345778906 |
| GOTERM_BP_DIRECT | GO:0001937~negative regulation of endothelial cell proliferation | 3 | 0.01 | 0.063233048 | SPARC, STAT1, TGFBR1 | 0.345778906 |
| GOTERM_BP_DIRECT | GO:0000077~DNA damage checkpoint | 3 | 0.01 | 0.067134874 | CHEK1, PTPN11, MAPK14 | 0.360428413 |
| GOTERM_BP_DIRECT | GO:0045776~negative regulation of blood pressure | 3 | 0.01 | 0.067134874 | NOS2, NOS3, PPARA | 0.360428413 |
| GOTERM_BP_DIRECT | GO:0060325~face morphogenesis | 3 | 0.01 | 0.067134874 | TGFB2, MMP2, PTPN11 | 0.360428413 |
| GOTERM_BP_DIRECT | GO:0033574~response to testosterone | 3 | 0.01 | 0.067134874 | GPI, MTAP, GBA | 0.360428413 |
| GOTERM_BP_DIRECT | GO:0048143~astrocyte activation | 2 | 0.01 | 0.068894467 | ADORA2A, EGFR | 0.360428413 |
| GOTERM_BP_DIRECT | GO:1990966~ATP generation from poly-ADP-D-ribose | 2 | 0.01 | 0.068894467 | NMNAT1, PARP1 | 0.360428413 |
| GOTERM_BP_DIRECT | GO:0060850~regulation of transcription involved in cell fate commitment | 2 | 0.01 | 0.068894467 | PPARG, RORA | 0.360428413 |
| GOTERM_BP_DIRECT | GO:0099565~chemical synaptic transmission, postsynaptic | 2 | 0.01 | 0.068894467 | GSK3B, AKT1 | 0.360428413 |
| GOTERM_BP_DIRECT | GO:0071801~regulation of podosome assembly | 2 | 0.01 | 0.068894467 | HCK, SRC | 0.360428413 |
| GOTERM_BP_DIRECT | GO:0007262~STAT protein import into nucleus | 2 | 0.01 | 0.068894467 | JAK2, JAK3 | 0.360428413 |
| GOTERM_BP_DIRECT | GO:0051180~vitamin transport | 2 | 0.01 | 0.068894467 | TTPA, GC | 0.360428413 |
| GOTERM_BP_DIRECT | GO:0072656~maintenance of protein location in mitochondrion | 2 | 0.01 | 0.068894467 | AKT1, HK1 | 0.360428413 |
| GOTERM_BP_DIRECT | GO:0007598~blood coagulation, extrinsic pathway | 2 | 0.01 | 0.068894467 | F7, F10 | 0.360428413 |
| GOTERM_BP_DIRECT | GO:0003417~growth plate cartilage development | 2 | 0.01 | 0.068894467 | MMP13, RARB | 0.360428413 |
| GOTERM_BP_DIRECT | GO:0050819~negative regulation of coagulation | 2 | 0.01 | 0.068894467 | PROCR, ANXA5 | 0.360428413 |
| GOTERM_BP_DIRECT | GO:0010891~negative regulation of sequestering of triglyceride | 2 | 0.01 | 0.068894467 | PPARG, PPARA | 0.360428413 |
| GOTERM_BP_DIRECT | GO:0035791~platelet-derived growth factor receptor-beta signaling pathway | 2 | 0.01 | 0.068894467 | PTPN1, ABL1 | 0.360428413 |
| GOTERM_BP_DIRECT | GO:0045329~carnitine biosynthetic process | 2 | 0.01 | 0.068894467 | SHMT1, ACADM | 0.360428413 |
| GOTERM_BP_DIRECT | GO:0035630~bone mineralization involved in bone maturation | 2 | 0.01 | 0.068894467 | BMP2, IGF1 | 0.360428413 |
| GOTERM_BP_DIRECT | GO:0048562~embryonic organ morphogenesis | 2 | 0.01 | 0.068894467 | RBP4, FGFR2 | 0.360428413 |
| GOTERM_BP_DIRECT | GO:0030901~midbrain development | 3 | 0.01 | 0.071114416 | CMA1, FGFR2, FGFR1 | 0.368848804 |
| GOTERM_BP_DIRECT | GO:0048701~embryonic cranial skeleton morphogenesis | 3 | 0.01 | 0.071114416 | TGFBR1, FGFR2, TGFBR2 | 0.368848804 |
| GOTERM_BP_DIRECT | GO:0030100~regulation of endocytosis | 3 | 0.01 | 0.071114416 | PTPN1, ABL1, RAB5A | 0.368848804 |
| GOTERM_BP_DIRECT | GO:0043200~response to amino acid | 3 | 0.01 | 0.071114416 | ARG1, CASP3, GSTP1 | 0.368848804 |
| GOTERM_BP_DIRECT | GO:0009749~response to glucose | 4 | 0.01 | 0.07207094 | CASP3, APOA2, TGFBR2, PPARD | 0.373009555 |
| GOTERM_BP_DIRECT | GO:0006629~lipid metabolic process | 6 | 0.02 | 0.072928179 | G6PD, FABP5, TTPA, PPARG, PPARA, PPARD | 0.376639763 |
| GOTERM_BP_DIRECT | GO:0035690~cellular response to drug | 4 | 0.01 | 0.074592839 | NOS2, PDE4B, REN, EGFR | 0.383296976 |
| GOTERM_BP_DIRECT | GO:0007411~axon guidance | 6 | 0.02 | 0.075110904 | TGFB2, MAPK1, GRB2, HRAS, BMP7, CDK5R1 | 0.383296976 |
| GOTERM_BP_DIRECT | GO:0045907~positive regulation of vasoconstriction | 3 | 0.01 | 0.075168708 | ABL1, AKT1, EGFR | 0.383296976 |
| GOTERM_BP_DIRECT | GO:0007093~mitotic cell cycle checkpoint | 3 | 0.01 | 0.075168708 | CHEK1, HRAS, BCL2L1 | 0.383296976 |
| GOTERM_BP_DIRECT | GO:0048705~skeletal system morphogenesis | 3 | 0.01 | 0.075168708 | TGFBR1, FGFR2, FGFR1 | 0.383296976 |
| GOTERM_BP_DIRECT | GO:0042220~response to cocaine | 3 | 0.01 | 0.075168708 | HSP90AA1, MDM2, HNMT | 0.383296976 |
| GOTERM_BP_DIRECT | GO:0006955~immune response | 11 | 0.03 | 0.076675548 | CHIT1, ZAP70, PNP, C1R, CMA1, CCL5, CTSG, BPI, CTSS, IL2, IGF1R | 0.390125649 |
| GOTERM_BP_DIRECT | GO:0033138~positive regulation of peptidyl-serine phosphorylation | 4 | 0.01 | 0.077153521 | CDC42, GSK3B, AKT1, MIF | 0.390125649 |
| GOTERM_BP_DIRECT | GO:0007265~Ras protein signal transduction | 4 | 0.01 | 0.077153521 | GRB2, IGF1, MAPK14, HRAS | 0.390125649 |
| GOTERM_BP_DIRECT | GO:0031647~regulation of protein stability | 4 | 0.01 | 0.077153521 | CDC42, HSPA8, APOA2, MAPK1 | 0.390125649 |
| GOTERM_BP_DIRECT | GO:0043123~positive regulation of I-kappaB kinase/NF-kappaB signaling | 6 | 0.02 | 0.07826347 | FKBP1A, CASP1, ABL1, HMOX1, RHOA, TGM2 | 0.394911917 |
| GOTERM_BP_DIRECT | GO:0070555~response to interleukin-1 | 3 | 0.01 | 0.079294853 | SRC, HNMT, SELE | 0.39762585 |
| GOTERM_BP_DIRECT | GO:1902042~negative regulation of extrinsic apoptotic signaling pathway via death domain receptors | 3 | 0.01 | 0.079294853 | DAPK1, NOS3, HMOX1 | 0.39762585 |
| GOTERM_BP_DIRECT | GO:2001244~positive regulation of intrinsic apoptotic signaling pathway | 3 | 0.01 | 0.079294853 | LCK, S100A9, BCL2L1 | 0.39762585 |
| GOTERM_BP_DIRECT | GO:0071347~cellular response to interleukin-1 | 4 | 0.01 | 0.079752473 | CCL5, LCN2, RORA, PCK1 | 0.399092603 |
| GOTERM_BP_DIRECT | GO:0046321~positive regulation of fatty acid oxidation | 2 | 0.01 | 0.082095344 | PPARG, PPARA | 0.400049286 |
| GOTERM_BP_DIRECT | GO:0060527~prostate epithelial cord arborization involved in prostate glandular acinus morphogenesis | 2 | 0.01 | 0.082095344 | ESR1, FGFR2 | 0.400049286 |
| GOTERM_BP_DIRECT | GO:0071638~negative regulation of monocyte chemotactic protein-1 production | 2 | 0.01 | 0.082095344 | GSTP1, NR1H4 | 0.400049286 |
| GOTERM_BP_DIRECT | GO:0010742~macrophage derived foam cell differentiation | 2 | 0.01 | 0.082095344 | STAT1, PPARG | 0.400049286 |
| GOTERM_BP_DIRECT | GO:0006739~NADP metabolic process | 2 | 0.01 | 0.082095344 | G6PD, GCK | 0.400049286 |
| GOTERM_BP_DIRECT | GO:0090330~regulation of platelet aggregation | 2 | 0.01 | 0.082095344 | ZAP70, SYK | 0.400049286 |
| GOTERM_BP_DIRECT | GO:0042423~catecholamine biosynthetic process | 2 | 0.01 | 0.082095344 | PNMT, PAH | 0.400049286 |
| GOTERM_BP_DIRECT | GO:0010042~response to manganese ion | 2 | 0.01 | 0.082095344 | ARG1, SOD2 | 0.400049286 |
| GOTERM_BP_DIRECT | GO:0006563~L-serine metabolic process | 2 | 0.01 | 0.082095344 | CBS, SHMT1 | 0.400049286 |
| GOTERM_BP_DIRECT | GO:0055012~ventricular cardiac muscle cell differentiation | 2 | 0.01 | 0.082095344 | RXRA, RARB | 0.400049286 |
| GOTERM_BP_DIRECT | GO:0097066~response to thyroid hormone | 2 | 0.01 | 0.082095344 | GBA, AKR1B1 | 0.400049286 |
| GOTERM_BP_DIRECT | GO:0032463~negative regulation of protein homooligomerization | 2 | 0.01 | 0.082095344 | SRC, GBA | 0.400049286 |
| GOTERM_BP_DIRECT | GO:0072655~establishment of protein localization to mitochondrion | 2 | 0.01 | 0.082095344 | AKT1, HK1 | 0.400049286 |
| GOTERM_BP_DIRECT | GO:0030282~bone mineralization | 3 | 0.01 | 0.08349002 | BMP2, MMP13, FGFR2 | 0.405211604 |
| GOTERM_BP_DIRECT | GO:0045787~positive regulation of cell cycle | 3 | 0.01 | 0.08349002 | TGFB2, FGFR2, FGFR1 | 0.405211604 |
| GOTERM_BP_DIRECT | GO:0009791~post-embryonic development | 4 | 0.01 | 0.085063088 | ACADM, SOD2, TGFBR1, FGFR2 | 0.412019003 |
| GOTERM_BP_DIRECT | GO:0031648~protein destabilization | 3 | 0.01 | 0.087751444 | BMP2, SRC, MDM2 | 0.420930533 |
| GOTERM_BP_DIRECT | GO:0048565~digestive tract development | 3 | 0.01 | 0.087751444 | KIT, FGFR2, TGFBR2 | 0.420930533 |
| GOTERM_BP_DIRECT | GO:0001890~placenta development | 3 | 0.01 | 0.087751444 | ANG, PPARG, MAPK14 | 0.420930533 |
| GOTERM_BP_DIRECT | GO:0042594~response to starvation | 3 | 0.01 | 0.087751444 | PPARG, ACADM, ACAT1 | 0.420930533 |
| GOTERM_BP_DIRECT | GO:0030334~regulation of cell migration | 4 | 0.01 | 0.087773682 | ERBB4, AKT2, AKT1, RHOA | 0.420930533 |
| GOTERM_BP_DIRECT | GO:0007417~central nervous system development | 5 | 0.01 | 0.091214517 | ARSA, ADORA2A, SRC, GSTP1, CSK | 0.434556672 |
| GOTERM_BP_DIRECT | GO:0090307~mitotic spindle assembly | 3 | 0.01 | 0.092076421 | XIAP, RHOA, RAB11A | 0.434556672 |
| GOTERM_BP_DIRECT | GO:0010507~negative regulation of autophagy | 3 | 0.01 | 0.092076421 | AKT1, MET, BCL2L1 | 0.434556672 |
| GOTERM_BP_DIRECT | GO:0042059~negative regulation of epidermal growth factor receptor signaling pathway | 3 | 0.01 | 0.092076421 | CDC42, GRB2, EGFR | 0.434556672 |
| GOTERM_BP_DIRECT | GO:0030177~positive regulation of Wnt signaling pathway | 3 | 0.01 | 0.092076421 | BMP2, CSNK2A1, FGFR2 | 0.434556672 |
| GOTERM_BP_DIRECT | GO:0048469~cell maturation | 3 | 0.01 | 0.092076421 | REN, PPARG, FGFR1 | 0.434556672 |
| GOTERM_BP_DIRECT | GO:0046500~S-adenosylmethionine metabolic process | 2 | 0.01 | 0.095109838 | BHMT, AMD1 | 0.434556672 |
| GOTERM_BP_DIRECT | GO:0009635~response to herbicide | 2 | 0.01 | 0.095109838 | ARG1, LCN2 | 0.434556672 |
| GOTERM_BP_DIRECT | GO:0048699~generation of neurons | 2 | 0.01 | 0.095109838 | TGFB2, CDK6 | 0.434556672 |
| GOTERM_BP_DIRECT | GO:0060463~lung lobe morphogenesis | 2 | 0.01 | 0.095109838 | FGFR2, TGFBR2 | 0.434556672 |
| GOTERM_BP_DIRECT | GO:0006029~proteoglycan metabolic process | 2 | 0.01 | 0.095109838 | BMP2, PPARD | 0.434556672 |
| GOTERM_BP_DIRECT | GO:0006595~polyamine metabolic process | 2 | 0.01 | 0.095109838 | AMD1, SRM | 0.434556672 |
| GOTERM_BP_DIRECT | GO:0042167~heme catabolic process | 2 | 0.01 | 0.095109838 | BLVRB, HMOX1 | 0.434556672 |
| GOTERM_BP_DIRECT | GO:0019048~modulation by virus of host morphology or physiology | 2 | 0.01 | 0.095109838 | RXRA, CD209 | 0.434556672 |
| GOTERM_BP_DIRECT | GO:0009072~aromatic amino acid family metabolic process | 2 | 0.01 | 0.095109838 | TPH1, PAH | 0.434556672 |
| GOTERM_BP_DIRECT | GO:0045656~negative regulation of monocyte differentiation | 2 | 0.01 | 0.095109838 | APCS, CDK6 | 0.434556672 |
| GOTERM_BP_DIRECT | GO:0043031~negative regulation of macrophage activation | 2 | 0.01 | 0.095109838 | NR1H3, BPI | 0.434556672 |
| GOTERM_BP_DIRECT | GO:0042178~xenobiotic catabolic process | 2 | 0.01 | 0.095109838 | GSTM1, GSTO1 | 0.434556672 |
| GOTERM_BP_DIRECT | GO:1901841~regulation of high voltage-gated calcium channel activity | 2 | 0.01 | 0.095109838 | PDE4B, CALM1 | 0.434556672 |
| GOTERM_BP_DIRECT | GO:0071312~cellular response to alkaloid | 2 | 0.01 | 0.095109838 | MDM2, BCL2L1 | 0.434556672 |
| GOTERM_BP_DIRECT | GO:2000121~regulation of removal of superoxide radicals | 2 | 0.01 | 0.095109838 | DHFR, BMP7 | 0.434556672 |
| GOTERM_BP_DIRECT | GO:0060501~positive regulation of epithelial cell proliferation involved in lung morphogenesis | 2 | 0.01 | 0.095109838 | CDC42, FGFR2 | 0.434556672 |
| GOTERM_BP_DIRECT | GO:0032287~peripheral nervous system myelin maintenance | 2 | 0.01 | 0.095109838 | AKT2, AKT1 | 0.434556672 |
| GOTERM_BP_DIRECT | GO:0035357~peroxisome proliferator activated receptor signaling pathway | 2 | 0.01 | 0.095109838 | RXRA, PPARG | 0.434556672 |
| GOTERM_BP_DIRECT | GO:0007435~salivary gland morphogenesis | 2 | 0.01 | 0.095109838 | TGFB2, EGFR | 0.434556672 |
| GOTERM_BP_DIRECT | GO:0007005~mitochondrion organization | 4 | 0.01 | 0.096120057 | ESRRA, PARP1, NOS3, GBA | 0.438343732 |
| GOTERM_BP_DIRECT | GO:0045786~negative regulation of cell cycle | 3 | 0.01 | 0.096462314 | BMP2, CDK6, BMP7 | 0.439076108 |
