# Supplementary material for: A Network Pharmacology Approach for Exploring the Mechanisms of Panax notoginseng Saponins in Ischaemic Stroke
Source: Evid Based Complement Alternat Med. 2021 Aug 13;2021:5582782. doi: 10.1155/2021/5582782 (PMC8382556; doi:10.1155/2021/5582782)
Supplement: Supplementary Materials — The supplementary materials are available online. Table S1: basic information of ingredients in PNS; Table S2: functions of potential target genes based on the GO molecular function; Table S3: functions of potential target genes based on the GO biological process; Table S4: functions of potential target genes based on the GO cellular component; Table S5: functions of potential target genes based on KEGG analysis. [file 5582782.f1.zip › 5582782.f1/Supplementary Table S4 Functions of potential target genes based on GO cellular component.docx]

Supplementary Table 4 Functions of potential target genes based on GO cellular component

| Category | Term | Count | Percent | PValue | Genes | FDR |
| --- | --- | --- | --- | --- | --- | --- |
| GOTERM_CC_DIRECT | GO:0005829~cytosol | 121 | 0.33 | 9.79E-30 | PNMT, HNMT, PNP, AKT2, CHEK1, AKT1, PRKACA, EPHB4, TGM2, MAP2K1, G6PD, CSNK2A1, AR, RBP4, MTAP, PADI4, PPIA, S100A9, CES1, TPH1, SHMT1, GLO1, PIK3R1, ADH5, TTPA, ABL1, PNPO, HMOX1, LTA4H, PCK1, HSPA8, GSTM1, EPHX2, DHFR, BHMT, CDK6, RHEB, PAH, LCN2, MDM2, GRB2, CALM1, RAB5A, BCL2L1, FGFR1, GPI, GSK3B, ARF1, THRA, AMD1, PDE3B, ADK, AKR1B1, PIK3CG, HK1, CASP7, CA1, IMPA1, CA2, CASP3, NCS1, PDE4B, CASP1, RAC2, JAK2, JAK3, HRAS, HSP90AA1, TPI1, SYK, PDPK1, ARG1, GSTO1, PDE4D, APOA2, DUSP6, RHOA, APRT, TGFBR2, ZAP70, HCK, LCK, BLVRB, PPARG, PDE5A, AHCY, ADH1C, SRC, ADH1B, GSTP1, NR1I3, XIAP, SRM, TYMP, CDC42, MAPK8, SULT1E1, ERBB4, CBS, CTNNA1, MAPK1, CSK, GC, PTPN1, NQO1, NOS2, STAT1, NOS3, GSR, PTPN11, MAPK14, RAB11A, GCK, MAPK10, FKBP1A, FABP3, FABP4, FABP5, FABP7, CDK5R1, HSPA1A | 2.43E-27 |
| GOTERM_CC_DIRECT | GO:0070062~extracellular exosome | 107 | 0.29 | 6.82E-27 | REG1A, ICAM2, HNMT, LGALS3, PNP, PLAU, PRKACA, EPHB4, TGM2, ARSA, MAP2K1, G6PD, RNASE3, MIF, PROCR, ACE2, RBP4, MTAP, PPIA, S100A9, CFB, RTN4R, CFD, MAOB, SHMT1, GLO1, NPR3, ADH5, ACAT1, DPP4, SEC14L2, PSAP, PNPO, LTA4H, PCK1, ELANE, HSPA8, EPHX2, INSR, LYZ, BHMT, CD209, RHEB, PAH, ALB, LCN2, GRB2, CALM1, SHBG, RAB5A, GPI, ARF1, SERPINA1, AKR1B1, ITGAL, HINT1, CA1, IMPA1, GM2A, ALDH2, CA2, NCS1, RAC2, CTSG, ACADM, CTSD, CTSB, HSP90AA1, MMP7, TPI1, MME, ARG1, GSTO1, ANXA5, APOA2, GP1BA, F2, MMP9, RHOA, APRT, LCK, BLVRB, ANG, BPI, APCS, AHCY, C1S, SRC, C1R, GSTP1, GBA, CDC42, TTR, MAPK1, CSK, GC, NQO1, F11, GSR, PLA2G2A, MAPK14, SOD2, RAB11A, FKBP1A, FABP3, FABP4, FABP5 | 8.46E-25 |
| GOTERM_CC_DIRECT | GO:0005615~extracellular space | 64 | 0.18 | 1.38E-19 | GPI, SERPINA1, SPARC, AKR1B1, CTSS, ADAMTS4, LGALS3, PLAU, CA2, CHEK1, CTSG, CTSD, CTSB, ARSA, MMP7, TPI1, ARG1, MMP2, MMP3, RNASE3, MIF, MMP8, F2, MMP9, PGF, CHIT1, ACE2, F7, RBP4, MMP13, KIT, ANG, BPI, S100A9, PPIA, CFB, CES1, CFD, APCS, GSTP1, GBA, EGFR, TTR, CCL5, PSAP, HMOX1, GC, ELANE, HSPA8, TGFB2, PLA2G2A, F11, IGF1, LYZ, SELE, BMP7, IL2, SELP, BMP2, FABP3, FAP, ALB, LCN2, REN | 1.14E-17 |
| GOTERM_CC_DIRECT | GO:0005576~extracellular region | 67 | 0.18 | 1.34E-17 | SERPINA1, SPARC, CTSS, ADAMTS4, PNP, PLAU, CASP1, KDR, CTSG, CTSD, EPHB4, CTSB, HSP90AA1, MMP7, MMP1, MMP2, MMP3, APOA2, RNASE3, MIF, MMP8, F2, MMP9, PGF, CHIT1, MMP12, ACE2, PROCR, F7, RBP4, MMP13, ANG, S100A9, MET, PPIA, CFB, CFD, APCS, C1S, C1R, ABO, TTR, ERBB4, CCL5, PSAP, GC, ELANE, BCHE, TGFB2, F10, CMA1, PLA2G2A, F11, IGF1, LYZ, BMP7, ESR2, IL2, BMP2, ALB, LCN2, REN, TEK, CALM1, SHBG, FGFR2, FGFR1 | 8.33E-16 |
| GOTERM_CC_DIRECT | GO:0045121~membrane raft | 18 | 0.05 | 1.95E-09 | SELE, EGFR, TGFBR1, TGFBR2, HK1, BACE1, DPP4, ACE2, ZAP70, ADAM17, LCK, CASP3, KDR, CSK, TEK, JAK2, CTSD, RAB5A | 9.65E-08 |
| GOTERM_CC_DIRECT | GO:0005925~focal adhesion | 23 | 0.06 | 1.07E-08 | HSPA8, MAP2K1, ARF1, MME, PDPK1, ANXA5, RHOA, EGFR, CDC42, DPP4, HCK, PROCR, ADAM17, FAP, PLAU, CTNNA1, RAC2, MAPK1, TEK, PPIA, HSPA1A, TGM2, EPHA2 | 4.44E-07 |
| GOTERM_CC_DIRECT | GO:0031012~extracellular matrix | 19 | 0.05 | 7.71E-08 | APCS, HSPA8, TGFB2, HSP90AA1, MMP7, MMP1, CMA1, MMP2, MMP8, BMP7, ADAMTS4, FKBP1A, LGALS3, MMP13, CTSG, CTSD, S100A9, FGFR2, TGM2 | 2.73E-06 |
| GOTERM_CC_DIRECT | GO:0031234~extrinsic component of cytoplasmic side of plasma membrane | 10 | 0.03 | 2.48E-07 | HCK, ZAP70, SYK, SRC, LCK, ABL1, CSK, JAK2, JAK3, RHOA | 7.70E-06 |
| GOTERM_CC_DIRECT | GO:0005886~plasma membrane | 89 | 0.24 | 4.06E-07 | GPI, GSK3B, ARF1, SPARC, ICAM2, ITGAL, PIK3CG, IGF1R, LGALS3, HINT1, STS, PLAU, CA2, CASP3, AKT2, NCS1, KDR, RAC2, AKT1, CTSG, PRKACA, HRAS, EPHB4, TGM2, MAP2K1, HSP90AA1, CSNK2A1, SYK, MME, PDPK1, DAPK1, MMP2, GP1BA, F2, RHOA, TGFBR1, TGFBR2, BACE1, ACE2, PROCR, F7, AR, ZAP70, ADAM17, ADORA2A, LCK, KIT, BLVRB, S100A9, MET, CFB, EPHA2, RTN4R, SRC, GSTP1, NPR3, PIK3R1, EGFR, DPP4, CDC42, ERBB4, CTNNA1, HMOX1, CSK, LTA4H, PTPN1, HSPA8, F10, NOS3, INSR, PLA2G2A, F11, IGF1, SELE, ESR1, RAB11A, SELP, MAPK10, FAP, CD209, MDM2, GRB2, REN, TEK, CALM1, RAB5A, FGFR2, CDK5R1, FGFR1 | 1.12E-05 |
| GOTERM_CC_DIRECT | GO:0005654~nucleoplasm | 67 | 0.18 | 6.32E-07 | GPI, THRA, ADK, AKR1B1, RORA, NR3C1, HNMT, NR3C2, CASP7, CASP3, AKT2, CHEK1, AKT1, JAK2, PRKACA, HADH, HSP90AA1, PARP1, CSNK2A1, PDPK1, MIF, DUSP6, APRT, AR, BLVRB, RARB, PPARG, PGR, PADI4, PPARA, PPARD, NR1I3, NR1I2, XIAP, RXRB, MAPK8, RXRA, ERBB4, ABL1, PNPO, MAPK1, LTA4H, ESRRA, HSPA8, STAT1, VDR, NR1H2, NR1H4, NR1H3, ESRRG, MAPK14, ESR1, ESR2, GCK, SELP, DHFR, MAPK10, CDK6, NMNAT1, FABP5, FABP7, MDM2, GRB2, CALM1, FGFR2, CDK5R1, HSPA1A | 1.57E-05 |
| GOTERM_CC_DIRECT | GO:0009986~cell surface | 24 | 0.07 | 8.01E-07 | RTN4R, HSP90AA1, SPARC, MMP7, GP1BA, MIF, ITGAL, EGFR, TGFBR1, BACE1, DPP4, ACE2, PROCR, BMP2, ADAM17, FAP, PLAU, CD209, CTSG, TEK, MET, ELANE, FGFR2, EPHA2 | 1.81E-05 |
| GOTERM_CC_DIRECT | GO:0005901~caveola | 9 | 0.02 | 2.06E-06 | HCK, SRC, NOS3, INSR, HMOX1, MAPK1, JAK2, SELE, TGFBR2 | 4.26E-05 |
| GOTERM_CC_DIRECT | GO:0005739~mitochondrion | 39 | 0.11 | 4.48E-06 | GSK3B, SPARC, MAOB, FECH, SRC, GSTP1, SHMT1, ADH5, HK1, ACAT1, MAPK8, GM2A, ERBB4, PSAP, CASP1, ABL1, AKT1, MAPK1, ACADM, HADH, PRKACA, TGM2, CTSB, ARG2, MAP2K1, PARP1, MMP2, GSR, PTPN11, MAPK14, SOD2, GCK, ESR2, RAB11A, MAPK10, CRAT, OTC, BCL2L1, HSPA1A | 8.54E-05 |
| GOTERM_CC_DIRECT | GO:0043235~receptor complex | 11 | 0.03 | 6.82E-06 | RXRA, ERBB4, VDR, INSR, NR1H3, EGFR, TGFBR1, NR3C2, FGFR1, IGF1R, TGFBR2 | 1.21E-04 |
| GOTERM_CC_DIRECT | GO:0043202~lysosomal lumen | 9 | 0.02 | 1.57E-05 | HSPA8, ARSA, HSP90AA1, GM2A, GBA, PSAP, GC, CTSD, CTSS | 2.60E-04 |
| GOTERM_CC_DIRECT | GO:0072562~blood microparticle | 11 | 0.03 | 3.29E-05 | HSPA8, APCS, BCHE, C1S, C1R, ALB, APOA2, F2, GC, CFB, HSPA1A | 5.10E-04 |
| GOTERM_CC_DIRECT | GO:0005737~cytoplasm | 98 | 0.27 | 4.55E-05 | SPARC, NR3C1, HNMT, LGALS3, PNP, AKT1, HADH, MAP2K1, G6PD, DAPK1, MIF, AR, ACE2, MTAP, ADAM17, PADI4, RTN4R, SHMT1, GLO1, PIK3R1, SEC14L2, PSAP, ABL1, PNPO, LTA4H, PCK1, ELANE, GSTM1, EPHX2, ESR1, SELP, BHMT, CDK6, CD209, MDM2, GRB2, CALM1, RAB5A, FGFR2, BCL2L1, GPI, GSK3B, ADK, AKR1B1, PIK3CG, HINT1, CASP7, CA1, IMPA1, CA2, CASP3, NCS1, RAC2, JAK2, HRAS, HSP90AA1, SYK, MME, PDPK1, ARG1, GSTO1, ANXA5, GP1BA, DUSP6, APRT, ZAP70, RARB, BLVRB, BPI, AHCY, SRC, GSTP1, NR1I3, XIAP, EGFR, CDC42, TTR, CBS, CCL5, MAPK1, CSK, NQO1, NOS2, STAT1, CMA1, NOS3, NR1H2, PTPN11, MAPK14, MAPK10, FKBP1A, FABP4, FABP5, FAP, FABP7, TEK, CDK5R1, HSPA1A | 6.64E-04 |
| GOTERM_CC_DIRECT | GO:0005911~cell-cell junction | 11 | 0.03 | 9.34E-05 | CDC42, ZAP70, ADAM17, FABP7, KIT, CTNNA1, AKT1, CSK, GRB2, TEK, PIK3R1 | 0.001286162 |
| GOTERM_CC_DIRECT | GO:0030424~axon | 11 | 0.03 | 7.22E-04 | BACE1, TGFB2, MME, STAT1, CA2, NCS1, MAPK1, ACADM, RAB5A, RAB11A, CDK5R1 | 0.009407622 |
| GOTERM_CC_DIRECT | GO:0031093~platelet alpha granule lumen | 6 | 0.02 | 7.59E-04 | CFD, TGFB2, SERPINA1, SPARC, ALB, IGF1 | 0.009407622 |
| GOTERM_CC_DIRECT | GO:0016020~membrane | 47 | 0.13 | 8.75E-04 | GPI, PDE3B, ICAM2, PIK3R1, ITGAL, ABO, EGFR, PIK3CG, IGF1R, DPP4, HSD11B1, CDC42, LGALS3, STS, PDE4B, RAC2, HMOX1, JAK2, PRKACA, HRAS, JAK3, BCHE, HSPA8, G6PD, HSP90AA1, PARP1, PDPK1, PDE4D, INSR, F11, ANXA5, GP1BA, ESR1, TGFBR1, PGF, RAB11A, SELP, FKBP1A, ADAM17, ADORA2A, CD209, RHEB, KIT, PPIA, FGFR2, CDK5R1, BCL2L1 | 0.010330556 |
| GOTERM_CC_DIRECT | GO:0043025~neuronal cell body | 13 | 0.04 | 9.66E-04 | RTN4R, NQO1, GSK3B, TGFB2, HSP90AA1, ARG1, CDC42, ADORA2A, FABP7, ABL1, ANG, RAB5A, CDK5R1 | 0.010785744 |
| GOTERM_CC_DIRECT | GO:0005788~endoplasmic reticulum lumen | 10 | 0.03 | 0.001000291 | BACE1, ARSA, F7, BCHE, SERPINA1, STS, F10, APOA2, F2, CES1 | 0.010785744 |
| GOTERM_CC_DIRECT | GO:0005759~mitochondrial matrix | 13 | 0.04 | 0.001334897 | ARG2, FECH, GSR, SOD2, NR3C1, ACAT1, ALDH2, ERBB4, ACADM, HADH, AGXT, OTC, BCL2L1 | 0.013793931 |
| GOTERM_CC_DIRECT | GO:0048471~perinuclear region of cytoplasm | 19 | 0.05 | 0.0014646 | GSK3B, ARF1, HSP90AA1, NOS2, STAT1, SRC, PLA2G2A, AKR1B1, SELE, EGFR, RAB11A, NCS1, ABL1, HMOX1, PPARG, HRAS, CTSB, HSPA1A, CDK5R1 | 0.014375919 |
| GOTERM_CC_DIRECT | GO:0031982~vesicle | 8 | 0.02 | 0.001507153 | F7, GSTP1, AKT1, MIF, CALM1, RHOA, ABO, RAB11A | 0.014375919 |
| GOTERM_CC_DIRECT | GO:0005578~proteinaceous extracellular matrix | 11 | 0.03 | 0.002913035 | MMP12, ADAMTS4, SERPINA1, MMP13, SPARC, MMP7, MMP1, MMP2, MMP3, MMP8, MMP9 | 0.026084054 |
| GOTERM_CC_DIRECT | GO:0005768~endosome | 10 | 0.03 | 0.002960904 | BACE1, ARSA, TGFB2, STS, KDR, GRB2, RAB5A, RHOA, EGFR, TGFBR1 | 0.026084054 |
| GOTERM_CC_DIRECT | GO:0005764~lysosome | 10 | 0.03 | 0.003050152 | CHIT1, ARSA, HCK, STS, SRC, PSAP, REN, CTSD, CTSS, CTSB | 0.026084054 |
| GOTERM_CC_DIRECT | GO:0043209~myelin sheath | 8 | 0.02 | 0.003961594 | CDC42, HSPA8, GPI, HSP90AA1, CA2, ALB, MIF, SOD2 | 0.032749177 |
| GOTERM_CC_DIRECT | GO:0043005~neuron projection | 10 | 0.03 | 0.004196148 | CDC42, RTN4R, GPI, ARF1, HSP90AA1, TPH1, AHCY, SRC, ARG1, HNMT | 0.033569185 |
| GOTERM_CC_DIRECT | GO:0032587~ruffle membrane | 6 | 0.02 | 0.004473745 | ADAM17, HSP90AA1, FAP, SRC, AKT2, EPHA2 | 0.034671525 |
| GOTERM_CC_DIRECT | GO:0005770~late endosome | 7 | 0.02 | 0.005460893 | BACE1, HSPA8, MAP2K1, ARF1, SRC, TTPA, MAPK1 | 0.04103944 |
| GOTERM_CC_DIRECT | GO:0043231~intracellular membrane-bounded organelle | 16 | 0.04 | 0.007005944 | G6PD, CMA1, INSR, CTSS, IGF1R, CYP2C9, CASP7, STS, NCS1, CHEK1, CTNNA1, PPARG, HRAS, FGFR2, CTSB, CDK5R1 | 0.051102179 |
| GOTERM_CC_DIRECT | GO:0043234~protein complex | 13 | 0.04 | 0.008406825 | HSP90AA1, SYK, PARP1, PTPN11, NR3C1, RAB11A, AR, RBP4, TTR, ALB, MDM2, AKT1, MAPK1 | 0.05956836 |
| GOTERM_CC_DIRECT | GO:0090575~RNA polymerase II transcription factor complex | 4 | 0.01 | 0.009093142 | RXRA, VDR, NR1H3, PPARG | 0.062641647 |
| GOTERM_CC_DIRECT | GO:0042470~melanosome | 6 | 0.02 | 0.010641179 | HSPA8, HSP90AA1, AHCY, CTSD, RAB5A, CTSB | 0.07132466 |
| GOTERM_CC_DIRECT | GO:0014069~postsynaptic density | 8 | 0.02 | 0.010938103 | GSK3B, ARF1, ADORA2A, PDPK1, SRC, NCS1, PDE4B, CDK5R1 | 0.071385517 |
| GOTERM_CC_DIRECT | GO:0005856~cytoskeleton | 11 | 0.03 | 0.025256848 | HCK, HINT1, PNP, NOS3, NR1I3, MAPK1, TEK, JAK2, JAK3, S100A9, RHOA | 0.156802177 |
| GOTERM_CC_DIRECT | GO:0031258~lamellipodium membrane | 3 | 0.01 | 0.025290674 | DPP4, FAP, EPHA2 | 0.156802177 |
| GOTERM_CC_DIRECT | GO:0042629~mast cell granule | 3 | 0.01 | 0.030532441 | KIT, AKR1B1, PIK3CG | 0.184684032 |
| GOTERM_CC_DIRECT | GO:0005634~nucleus | 85 | 0.23 | 0.039143445 | GSK3B, THRA, ADK, RORA, NR3C1, NR3C2, LGALS3, HINT1, CASP7, PNP, CASP3, AKT2, CHEK1, KDR, AKT1, CTSG, ACADM, JAK2, PRKACA, HRAS, MAP2K1, G6PD, HSP90AA1, TPI1, PARP1, CSNK2A1, SYK, ARG1, MMP2, AR, HCK, MTAP, RARB, ANG, PPARG, PGR, PADI4, PPARA, S100A9, PPIA, PPARD, APCS, AHCY, SRC, GSTP1, SHMT1, NR1I3, NR1I2, XIAP, PIK3R1, EGFR, RXRB, SEC14L2, MAPK8, RXRA, ERBB4, CBS, ABL1, HMOX1, MAPK1, LTA4H, ESRRA, HSPA8, NOS2, NOS3, STAT1, VDR, NR1H2, NR1H4, NR1H3, PTPN11, ESRRG, MAPK14, ESR1, ESR2, FABP4, CDK6, NMNAT1, ALB, MDM2, GRB2, CALM1, FGFR2, CDK5R1, FGFR1 | 0.231132723 |
| GOTERM_CC_DIRECT | GO:0034704~calcium channel complex | 3 | 0.01 | 0.042156939 | PDE4D, CALM1, PRKACA | 0.240399593 |
| GOTERM_CC_DIRECT | GO:0005623~cell | 5 | 0.01 | 0.042651541 | LCN2, GCK, IL2, TGFBR1, HK1 | 0.240399593 |
| GOTERM_CC_DIRECT | GO:0005777~peroxisome | 5 | 0.01 | 0.04806813 | NOS2, EPHX2, ACADM, AGXT, CRAT | 0.260346796 |
| GOTERM_CC_DIRECT | GO:0035867~alphav-beta3 integrin-IGF-1-IGF1R complex | 2 | 0.01 | 0.051439488 | IGF1, IGF1R | 0.260346796 |
| GOTERM_CC_DIRECT | GO:0097443~sorting endosome | 2 | 0.01 | 0.051439488 | PTPN1, KDR | 0.260346796 |
| GOTERM_CC_DIRECT | GO:0036021~endolysosome lumen | 2 | 0.01 | 0.051439488 | CTSS, CTSB | 0.260346796 |
| GOTERM_CC_DIRECT | GO:0070022~transforming growth factor beta receptor homodimeric complex | 2 | 0.01 | 0.051439488 | TGFBR1, TGFBR2 | 0.260346796 |
| GOTERM_CC_DIRECT | GO:0005884~actin filament | 4 | 0.01 | 0.053761471 | HCK, SRC, RAC2, TEK | 0.266656895 |
| GOTERM_CC_DIRECT | GO:0005813~centrosome | 11 | 0.03 | 0.055268753 | PROCR, GSK3B, G6PD, CDK6, PDE4D, CHEK1, PDE4B, CALM1, PRKACA, RAB11A, BCL2L1 | 0.268757856 |
| GOTERM_CC_DIRECT | GO:0030027~lamellipodium | 6 | 0.02 | 0.059885762 | DPP4, FAP, CTNNA1, RAC2, RHOA, EPHA2 | 0.285609018 |
| GOTERM_CC_DIRECT | GO:0005794~Golgi apparatus | 18 | 0.05 | 0.062484221 | MAP2K1, SERPINA1, PDE3B, ESR1, ABO, RAB11A, BACE1, HCK, STS, ALB, NCS1, KDR, CTNNA1, MAPK1, GRB2, PPARG, HRAS, FGFR2 | 0.292378997 |
| GOTERM_CC_DIRECT | GO:0030426~growth cone | 5 | 0.01 | 0.066483457 | GSK3B, ABL1, ANG, CALM1, CDK5R1 | 0.305331432 |
| GOTERM_CC_DIRECT | GO:0005622~intracellular | 25 | 0.07 | 0.070450053 | GSTP1, NPR3, PIK3CG, CDC42, SEC14L2, MAPK8, PNP, RAC2, MAPK1, HRAS, TGM2, CTSB, HSPA8, NOS2, PDPK1, ANXA5, MAPK14, RHOA, TGFBR1, IL2, RAB11A, MAPK10, RHEB, RAB5A, EPHA2 | 0.317665693 |
| GOTERM_CC_DIRECT | GO:0001772~immunological synapse | 3 | 0.01 | 0.073057989 | LGALS3, ZAP70, LCK | 0.319167985 |
| GOTERM_CC_DIRECT | GO:0030141~secretory granule | 4 | 0.01 | 0.073357158 | CDC42, PLA2G2A, CTSG, ELANE | 0.319167985 |
| GOTERM_CC_DIRECT | GO:0042995~cell projection | 4 | 0.01 | 0.075706463 | CDC42, HCK, PDPK1, FABP7 | 0.320140013 |
| GOTERM_CC_DIRECT | GO:0071438~invadopodium membrane | 2 | 0.01 | 0.076162342 | DPP4, FAP | 0.320140013 |
| GOTERM_CC_DIRECT | GO:0005769~early endosome | 7 | 0.02 | 0.081566382 | PTPN1, MAP2K1, AKT2, APOA2, KDR, MAPK1, RAB5A | 0.337141047 |
| GOTERM_CC_DIRECT | GO:0030017~sarcomere | 3 | 0.01 | 0.092536624 | ARF1, MMP2, CALM1 | 0.366298638 |
| GOTERM_CC_DIRECT | GO:0071944~cell periphery | 3 | 0.01 | 0.092536624 | FABP7, PDE4B, RHOA | 0.366298638 |
| GOTERM_CC_DIRECT | GO:0001669~acrosomal vesicle | 4 | 0.01 | 0.09305167 | ARSA, KIT, CTNNA1, PRKACA | 0.366298638 |
