# Supplementary material for: A Network Pharmacology Approach for Exploring the Mechanisms of Panax notoginseng Saponins in Ischaemic Stroke
Source: Evid Based Complement Alternat Med. 2021 Aug 13;2021:5582782. doi: 10.1155/2021/5582782 (PMC8382556; doi:10.1155/2021/5582782)
Supplement: Supplementary Materials — The supplementary materials are available online. Table S1: basic information of ingredients in PNS; Table S2: functions of potential target genes based on the GO molecular function; Table S3: functions of potential target genes based on the GO biological process; Table S4: functions of potential target genes based on the GO cellular component; Table S5: functions of potential target genes based on KEGG analysis. [file 5582782.f1.zip › 5582782.f1/Supplementary Table S5 Functions of potential target genes based on KEGG analysis..docx]

Supplementary Table 5 Functions of potential target genes based on KEGG analysis.

| Category | Term | Count | Percent | PValue | Genes | FDR |
| --- | --- | --- | --- | --- | --- | --- |
| KEGG_PATHWAY | bta05200:Pathways in cancer | 47 | 0.13 | 4.02E-18 | GSK3B, XIAP, PIK3R1, PIK3CG, EGFR, IGF1R, CDC42, RXRB, MAPK8, RXRA, AKT2, CASP3, ABL1, CTNNA1, RAC2, AKT1, MAPK1, PRKACA, HRAS, MAP2K1, HSP90AA1, TGFB2, NOS2, DAPK1, MMP1, STAT1, MMP2, IGF1, MMP9, RHOA, TGFBR1, PGF, TGFBR2, MAPK10, AR, BMP2, CDK6, KIT, MDM2, RARB, GRB2, PPARG, MET, FGFR2, PPARD, FGFR1, BCL2L1 | 5.51E-16 |
| KEGG_PATHWAY | bta04014:Ras signaling pathway | 34 | 0.09 | 1.56E-15 | PIK3R1, PIK3CG, EGFR, IGF1R, CDC42, MAPK8, AKT2, KDR, ABL1, RAC2, AKT1, MAPK1, PRKACA, HRAS, MAP2K1, INSR, PLA2G2A, PTPN11, IGF1, RHOA, PGF, MAPK10, ZAP70, KIT, GRB2, TEK, CALM1, MET, RAB5A, FGFR2, EPHA2, FGFR1, BCL2L1 | 1.07E-13 |
| KEGG_PATHWAY | bta05205:Proteoglycans in cancer | 29 | 0.08 | 4.25E-13 | SRC, PIK3R1, PIK3CG, EGFR, IGF1R, CDC42, PLAU, ERBB4, AKT2, CASP3, KDR, AKT1, MAPK1, PRKACA, HRAS, MAP2K1, TGFB2, PDPK1, MMP2, PTPN11, IGF1, MAPK14, ESR1, MMP9, RHOA, MDM2, GRB2, MET, FGFR1 | 1.94E-11 |
| KEGG_PATHWAY | bta04915:Estrogen signaling pathway | 21 | 0.06 | 6.90E-13 | HSPA8, MAP2K1, HSP90AA1, NOS3, SRC, MMP2, PIK3R1, ESR1, MMP9, EGFR, PIK3CG, ESR2, AKT2, AKT1, MAPK1, GRB2, CALM1, PRKACA, HRAS, HSPA1A | 2.36E-11 |
| KEGG_PATHWAY | bta04068:FoxO signaling pathway | 23 | 0.06 | 3.50E-12 | MAP2K1, TGFB2, PDPK1, INSR, PIK3R1, IGF1, SOD2, MAPK14, TGFBR1, EGFR, PIK3CG, TGFBR2, IGF1R, MAPK10, MAPK8, AKT2, MDM2, AKT1, MAPK1, GRB2, PCK1, HRAS | 9.58E-11 |
| KEGG_PATHWAY | bta04917:Prolactin signaling pathway | 18 | 0.05 | 5.21E-12 | GSK3B, MAP2K1, SRC, STAT1, PIK3R1, MAPK14, ESR1, GCK, PIK3CG, ESR2, MAPK10, MAPK8, AKT2, AKT1, MAPK1, GRB2, JAK2, HRAS | 1.19E-10 |
| KEGG_PATHWAY | bta05212:Pancreatic cancer | 17 | 0.05 | 7.21E-12 | MAP2K1, TGFB2, STAT1, PIK3R1, TGFBR1, EGFR, PIK3CG, TGFBR2, CDC42, MAPK10, MAPK8, CDK6, AKT2, RAC2, AKT1, MAPK1, BCL2L1 | 1.41E-10 |
| KEGG_PATHWAY | bta04015:Rap1 signaling pathway | 27 | 0.07 | 5.93E-11 | SRC, PIK3R1, ITGAL, PIK3CG, EGFR, IGF1R, CDC42, AKT2, KDR, RAC2, AKT1, MAPK1, HRAS, MAP2K1, INSR, IGF1, MAPK14, RHOA, PGF, ADORA2A, KIT, TEK, CALM1, MET, FGFR2, EPHA2, FGFR1 | 1.02E-09 |
| KEGG_PATHWAY | bta05215:Prostate cancer | 18 | 0.05 | 6.83E-11 | GSK3B, MAP2K1, HSP90AA1, PDPK1, PIK3R1, IGF1, EGFR, PIK3CG, IGF1R, AR, AKT2, MDM2, AKT1, MAPK1, GRB2, HRAS, FGFR2, FGFR1 | 1.04E-09 |
| KEGG_PATHWAY | bta04151:PI3K-Akt signaling pathway | 34 | 0.09 | 1.06E-10 | GSK3B, PIK3R1, PIK3CG, EGFR, IGF1R, RXRA, AKT2, KDR, AKT1, MAPK1, PCK1, JAK2, HRAS, JAK3, MAP2K1, HSP90AA1, SYK, PDPK1, NOS3, INSR, IGF1, IL2, PGF, CDK6, RHEB, KIT, MDM2, GRB2, TEK, MET, FGFR2, EPHA2, FGFR1, BCL2L1 | 1.45E-09 |
| KEGG_PATHWAY | bta04910:Insulin signaling pathway | 21 | 0.06 | 3.69E-10 | PTPN1, GSK3B, MAP2K1, PDPK1, INSR, PDE3B, PIK3R1, GCK, PIK3CG, HK1, MAPK10, MAPK8, RHEB, AKT2, AKT1, MAPK1, GRB2, PCK1, CALM1, PRKACA, HRAS | 4.59E-09 |
| KEGG_PATHWAY | bta05220:Chronic myeloid leukemia | 16 | 0.04 | 5.30E-10 | MAP2K1, TGFB2, PTPN11, PIK3R1, TGFBR1, PIK3CG, TGFBR2, CDK6, AKT2, ABL1, MDM2, AKT1, MAPK1, GRB2, HRAS, BCL2L1 | 5.69E-09 |
| KEGG_PATHWAY | bta05230:Central carbon metabolism in cancer | 15 | 0.04 | 5.40E-10 | MAP2K1, G6PD, PIK3R1, GCK, EGFR, PIK3CG, HK1, AKT2, KIT, AKT1, MAPK1, HRAS, MET, FGFR2, FGFR1 | 5.69E-09 |
| KEGG_PATHWAY | bta05210:Colorectal cancer | 15 | 0.04 | 1.31E-09 | GSK3B, MAP2K1, TGFB2, PIK3R1, RHOA, TGFBR1, PIK3CG, TGFBR2, MAPK10, MAPK8, AKT2, CASP3, RAC2, AKT1, MAPK1 | 1.28E-08 |
| KEGG_PATHWAY | bta05223:Non-small cell lung cancer | 14 | 0.04 | 1.58E-09 | MAP2K1, PDPK1, PIK3R1, EGFR, PIK3CG, RXRB, RXRA, CDK6, AKT2, RARB, AKT1, MAPK1, GRB2, HRAS | 1.45E-08 |
| KEGG_PATHWAY | bta04520:Adherens junction | 15 | 0.04 | 1.99E-09 | PTPN1, CSNK2A1, SRC, INSR, RHOA, TGFBR1, EGFR, TGFBR2, IGF1R, CDC42, CTNNA1, RAC2, MAPK1, MET, FGFR1 | 1.70E-08 |
| KEGG_PATHWAY | bta03320:PPAR signaling pathway | 15 | 0.04 | 2.98E-09 | PDPK1, MMP1, APOA2, NR1H3, RXRB, FABP3, RXRA, FABP4, FABP5, FABP7, PPARG, ACADM, PCK1, PPARA, PPARD | 2.40E-08 |
| KEGG_PATHWAY | bta04919:Thyroid hormone signaling pathway | 18 | 0.05 | 5.76E-09 | GSK3B, MAP2K1, THRA, PDPK1, SRC, STAT1, PIK3R1, ESR1, PIK3CG, RXRB, RXRA, RHEB, AKT2, MDM2, AKT1, MAPK1, PRKACA, HRAS | 4.39E-08 |
| KEGG_PATHWAY | bta05214:Glioma | 14 | 0.04 | 1.11E-08 | MAP2K1, PIK3R1, IGF1, EGFR, PIK3CG, IGF1R, CDK6, AKT2, MDM2, AKT1, MAPK1, GRB2, CALM1, HRAS | 8.03E-08 |
| KEGG_PATHWAY | bta04664:Fc epsilon RI signaling pathway | 14 | 0.04 | 1.64E-08 | MAP2K1, SYK, PDPK1, PIK3R1, MAPK14, PIK3CG, MAPK10, MAPK8, AKT2, RAC2, AKT1, MAPK1, GRB2, HRAS | 1.12E-07 |
| KEGG_PATHWAY | bta04510:Focal adhesion | 23 | 0.06 | 2.66E-08 | GSK3B, MAP2K1, PDPK1, SRC, XIAP, PIK3R1, IGF1, RHOA, EGFR, PIK3CG, PGF, IGF1R, CDC42, MAPK10, MAPK8, AKT2, KDR, RAC2, AKT1, MAPK1, GRB2, HRAS, MET | 1.71E-07 |
| KEGG_PATHWAY | bta04722:Neurotrophin signaling pathway | 18 | 0.05 | 2.75E-08 | GSK3B, MAP2K1, PDPK1, PTPN11, PIK3R1, MAPK14, RHOA, PIK3CG, CDC42, MAPK10, MAPK8, AKT2, ABL1, AKT1, MAPK1, GRB2, CALM1, HRAS | 1.71E-07 |
| KEGG_PATHWAY | bta04370:VEGF signaling pathway | 13 | 0.04 | 3.41E-08 | MAP2K1, NOS3, SRC, PIK3R1, MAPK14, PIK3CG, CDC42, AKT2, KDR, RAC2, AKT1, MAPK1, HRAS | 2.03E-07 |
| KEGG_PATHWAY | bta05145:Toxoplasmosis | 17 | 0.05 | 4.01E-08 | HSPA8, TGFB2, NOS2, PDPK1, STAT1, XIAP, MAPK14, MAPK10, MAPK8, AKT2, CASP3, AKT1, MAPK1, JAK2, HSPA1A, BCL2L1 | 2.24E-07 |
| KEGG_PATHWAY | bta05218:Melanoma | 14 | 0.04 | 4.08E-08 | MAP2K1, PIK3R1, IGF1, EGFR, PIK3CG, IGF1R, CDK6, AKT2, MDM2, AKT1, MAPK1, HRAS, MET, FGFR1 | 2.24E-07 |
| KEGG_PATHWAY | bta04012:ErbB signaling pathway | 15 | 0.04 | 4.80E-08 | GSK3B, MAP2K1, SRC, PIK3R1, EGFR, PIK3CG, MAPK10, MAPK8, ERBB4, AKT2, ABL1, AKT1, MAPK1, GRB2, HRAS | 2.53E-07 |
| KEGG_PATHWAY | bta04914:Progesterone-mediated oocyte maturation | 15 | 0.04 | 6.49E-08 | MAP2K1, HSP90AA1, PDE3B, PIK3R1, IGF1, MAPK14, PIK3CG, IGF1R, MAPK10, MAPK8, AKT2, AKT1, MAPK1, PGR, PRKACA | 3.30E-07 |
| KEGG_PATHWAY | bta05213:Endometrial cancer | 12 | 0.03 | 6.79E-08 | GSK3B, MAP2K1, PDPK1, AKT2, CTNNA1, AKT1, MAPK1, GRB2, PIK3R1, HRAS, EGFR, PIK3CG | 3.32E-07 |
| KEGG_PATHWAY | bta04660:T cell receptor signaling pathway | 16 | 0.04 | 9.59E-08 | GSK3B, MAP2K1, PDPK1, PIK3R1, MAPK14, RHOA, IL2, PIK3CG, CDC42, ZAP70, LCK, AKT2, AKT1, MAPK1, GRB2, HRAS | 4.53E-07 |
| KEGG_PATHWAY | bta04931:Insulin resistance | 16 | 0.04 | 2.03E-07 | PTPN1, GSK3B, PDPK1, NOS3, NR1H2, INSR, NR1H3, PTPN11, PIK3R1, PIK3CG, MAPK10, MAPK8, AKT2, AKT1, PCK1, PPARA | 9.29E-07 |
| KEGG_PATHWAY | bta05152:Tuberculosis | 20 | 0.05 | 2.83E-07 | TGFB2, NOS2, SYK, VDR, SRC, STAT1, MAPK14, RHOA, CTSS, MAPK10, MAPK8, CD209, AKT2, CASP3, AKT1, MAPK1, CALM1, JAK2, CTSD, RAB5A | 1.25E-06 |
| KEGG_PATHWAY | bta05160:Hepatitis C | 17 | 0.05 | 4.05E-07 | GSK3B, PDPK1, STAT1, NR1H3, PIK3R1, MAPK14, EGFR, PIK3CG, MAPK10, MAPK8, RXRA, AKT2, AKT1, MAPK1, GRB2, PPARA, HRAS | 1.73E-06 |
| KEGG_PATHWAY | bta04380:Osteoclast differentiation | 17 | 0.05 | 4.49E-07 | MAP2K1, TGFB2, SYK, STAT1, PIK3R1, MAPK14, TGFBR1, PIK3CG, TGFBR2, MAPK10, MAPK8, LCK, AKT2, AKT1, MAPK1, GRB2, PPARG | 1.86E-06 |
| KEGG_PATHWAY | bta04550:Signaling pathways regulating pluripotency of stem cells | 17 | 0.05 | 7.43E-07 | GSK3B, MAP2K1, PIK3R1, IGF1, MAPK14, PIK3CG, IGF1R, BMP2, AKT2, AKT1, MAPK1, GRB2, JAK2, HRAS, JAK3, FGFR2, FGFR1 | 2.94E-06 |
| KEGG_PATHWAY | bta05219:Bladder cancer | 10 | 0.03 | 7.51E-07 | MAP2K1, DAPK1, SRC, MMP1, MMP2, MDM2, MAPK1, HRAS, MMP9, EGFR | 2.94E-06 |
| KEGG_PATHWAY | bta04668:TNF signaling pathway | 15 | 0.04 | 8.83E-07 | MAP2K1, MMP3, PIK3R1, MAPK14, SELE, MMP9, PIK3CG, MAPK10, MAPK8, CASP7, AKT2, CCL5, CASP3, AKT1, MAPK1 | 3.36E-06 |
| KEGG_PATHWAY | bta04010:MAPK signaling pathway | 23 | 0.06 | 9.78E-07 | HSPA8, MAP2K1, TGFB2, MAPK14, DUSP6, TGFBR1, EGFR, TGFBR2, CDC42, MAPK10, MAPK8, AKT2, CASP3, RAC2, AKT1, MAPK1, GRB2, PRKACA, HRAS, FGFR2, HSPA1A, FGFR1 | 3.62E-06 |
| KEGG_PATHWAY | bta05211:Renal cell carcinoma | 12 | 0.03 | 1.08E-06 | CDC42, MAP2K1, TGFB2, AKT2, AKT1, MAPK1, GRB2, PTPN11, PIK3R1, HRAS, MET, PIK3CG | 3.89E-06 |
| KEGG_PATHWAY | bta04066:HIF-1 signaling pathway | 14 | 0.04 | 1.32E-06 | MAP2K1, NOS2, NOS3, INSR, PIK3R1, IGF1, EGFR, PIK3CG, HK1, IGF1R, AKT2, AKT1, MAPK1, TEK | 4.65E-06 |
| KEGG_PATHWAY | bta05161:Hepatitis B | 17 | 0.05 | 1.74E-06 | MAP2K1, TGFB2, SRC, STAT1, PIK3R1, MMP9, TGFBR1, PIK3CG, MAPK10, MAPK8, CDK6, AKT2, CASP3, AKT1, MAPK1, GRB2, HRAS | 5.95E-06 |
| KEGG_PATHWAY | bta04062:Chemokine signaling pathway | 19 | 0.05 | 1.81E-06 | GSK3B, MAP2K1, SRC, STAT1, PIK3R1, RHOA, PIK3CG, CDC42, HCK, AKT2, CCL5, RAC2, AKT1, MAPK1, GRB2, JAK2, PRKACA, HRAS, JAK3 | 6.06E-06 |
| KEGG_PATHWAY | bta05231:Choline metabolism in cancer | 14 | 0.04 | 1.89E-06 | MAP2K1, PDPK1, PIK3R1, EGFR, PIK3CG, MAPK10, MAPK8, RHEB, AKT2, RAC2, AKT1, MAPK1, GRB2, HRAS | 6.17E-06 |
| KEGG_PATHWAY | bta04912:GnRH signaling pathway | 13 | 0.04 | 2.48E-06 | MAP2K1, SRC, MMP2, MAPK14, EGFR, CDC42, MAPK10, MAPK8, MAPK1, GRB2, CALM1, PRKACA, HRAS | 7.89E-06 |
| KEGG_PATHWAY | bta04071:Sphingolipid signaling pathway | 15 | 0.04 | 3.19E-06 | MAP2K1, PDPK1, NOS3, PIK3R1, MAPK14, RHOA, PIK3CG, MAPK10, MAPK8, AKT2, RAC2, AKT1, MAPK1, HRAS, CTSD | 9.94E-06 |
| KEGG_PATHWAY | bta05133:Pertussis | 12 | 0.03 | 5.20E-06 | MAPK10, MAPK8, CASP7, NOS2, C1S, C1R, CASP3, CASP1, MAPK1, MAPK14, CALM1, RHOA | 1.58E-05 |
| KEGG_PATHWAY | bta05142:Chagas disease (American trypanosomiasis) | 14 | 0.04 | 9.34E-06 | TGFB2, NOS2, PIK3R1, MAPK14, TGFBR1, IL2, PIK3CG, TGFBR2, MAPK10, MAPK8, AKT2, CCL5, AKT1, MAPK1 | 2.78E-05 |
| KEGG_PATHWAY | bta05164:Influenza A | 17 | 0.05 | 1.33E-05 | HSPA8, GSK3B, MAP2K1, STAT1, PIK3R1, MAPK14, PIK3CG, MAPK10, MAPK8, AKT2, CCL5, CASP1, AKT1, MAPK1, JAK2, HSPA1A | 3.88E-05 |
| KEGG_PATHWAY | bta04662:B cell receptor signaling pathway | 11 | 0.03 | 1.41E-05 | GSK3B, MAP2K1, SYK, AKT2, RAC2, AKT1, MAPK1, GRB2, PIK3R1, HRAS, PIK3CG | 4.02E-05 |
| KEGG_PATHWAY | bta05221:Acute myeloid leukemia | 10 | 0.03 | 1.44E-05 | MAP2K1, AKT2, KIT, AKT1, MAPK1, GRB2, PIK3R1, HRAS, PIK3CG, PPARD | 4.02E-05 |
| KEGG_PATHWAY | bta05162:Measles | 15 | 0.04 | 1.94E-05 | HSPA8, GSK3B, CSNK2A1, STAT1, PIK3R1, IL2, PIK3CG, CDK6, CD209, AKT2, AKT1, JAK2, JAK3, HSPA1A | 5.33E-05 |
| KEGG_PATHWAY | bta01130:Biosynthesis of antibiotics | 18 | 0.05 | 3.18E-05 | GPI, ARG2, G6PD, TPI1, ARG1, SHMT1, HMGCR, GCK, ADH5, ACAT1, HK1, ALDH2, CBS, ACADM, PCK1, AGXT, HADH, OTC | 8.54E-05 |
| KEGG_PATHWAY | bta04650:Natural killer cell mediated cytotoxicity | 13 | 0.04 | 6.03E-05 | MAP2K1, SYK, PTPN11, PIK3R1, ITGAL, PIK3CG, ZAP70, LCK, CASP3, RAC2, MAPK1, GRB2, HRAS | 1.59E-04 |
| KEGG_PATHWAY | bta04024:cAMP signaling pathway | 17 | 0.05 | 7.03E-05 | MAP2K1, PDE4D, PDE3B, PIK3R1, RHOA, PIK3CG, MAPK10, MAPK8, ADORA2A, AKT2, PDE4B, RAC2, AKT1, MAPK1, PPARA, CALM1, PRKACA | 1.82E-04 |
| KEGG_PATHWAY | bta00480:Glutathione metabolism | 9 | 0.02 | 7.98E-05 | G6PD, GSTM1, GSTO1, GSTP1, GSR, SRM | 2.02E-04 |
| KEGG_PATHWAY | bta05222:Small cell lung cancer | 11 | 0.03 | 9.60E-05 | RXRB, RXRA, CDK6, NOS2, AKT2, RARB, AKT1, XIAP, PIK3R1, PIK3CG, BCL2L1 | 2.39E-04 |
| KEGG_PATHWAY | bta04920:Adipocytokine signaling pathway | 10 | 0.03 | 1.00E-04 | MAPK10, RXRB, MAPK8, RXRA, AKT2, AKT1, PTPN11, PCK1, JAK2, PPARA | 2.45E-04 |
| KEGG_PATHWAY | bta00982:Drug metabolism - cytochrome P450 | 9 | 0.02 | 1.34E-04 | GSTM1, ADH1C, MAOB, GSTO1, GSTP1, ADH5 | 3.22E-04 |
| KEGG_PATHWAY | bta04610:Complement and coagulation cascades | 10 | 0.03 | 1.39E-04 | CFD, F7, SERPINA1, F10, C1S, PLAU, C1R, F11, F2, CFB | 3.28E-04 |
| KEGG_PATHWAY | bta00980:Metabolism of xenobiotics by cytochrome P450 | 9 | 0.02 | 1.52E-04 | HSD11B1, GSTM1, ADH1C, GSTO1, GSTP1, ADH5 | 3.52E-04 |
| KEGG_PATHWAY | bta04930:Type II diabetes mellitus | 8 | 0.02 | 2.17E-04 | MAPK10, MAPK8, INSR, MAPK1, PIK3R1, PIK3CG, GCK, HK1 | 4.97E-04 |
| KEGG_PATHWAY | bta04932:Non-alcoholic fatty liver disease (NAFLD) | 14 | 0.04 | 3.23E-04 | GSK3B, INSR, NR1H3, PIK3R1, PIK3CG, CDC42, MAPK10, MAPK8, CASP7, RXRA, AKT2, CASP3, AKT1, PPARA | 7.13E-04 |
| KEGG_PATHWAY | bta00330:Arginine and proline metabolism | 8 | 0.02 | 3.23E-04 | ARG2, ALDH2, MAOB, NOS2, NOS3, ARG1, AMD1, SRM | 7.13E-04 |
| KEGG_PATHWAY | bta04152:AMPK signaling pathway | 12 | 0.03 | 3.33E-04 | PDPK1, RHEB, AKT2, INSR, AKT1, PPARG, HMGCR, IGF1, PIK3R1, PCK1, PIK3CG, IGF1R | 7.24E-04 |
| KEGG_PATHWAY | bta04666:Fc gamma R-mediated phagocytosis | 10 | 0.03 | 4.02E-04 | CDC42, HCK, MAP2K1, SYK, AKT2, RAC2, AKT1, MAPK1, PIK3R1, PIK3CG | 8.60E-04 |
| KEGG_PATHWAY | bta04620:Toll-like receptor signaling pathway | 11 | 0.03 | 4.57E-04 | MAPK10, MAP2K1, MAPK8, STAT1, AKT2, CCL5, AKT1, MAPK1, PIK3R1, MAPK14, PIK3CG | 9.63E-04 |
| KEGG_PATHWAY | bta05204:Chemical carcinogenesis | 9 | 0.02 | 5.03E-04 | HSD11B1, GSTM1, ADH1C, GSTO1, GSTP1, ADH5 | 0.001044096 |
| KEGG_PATHWAY | bta04960:Aldosterone-regulated sodium reabsorption | 7 | 0.02 | 5.18E-04 | PDPK1, INSR, MAPK1, IGF1, PIK3R1, PIK3CG, NR3C2 | 0.001058612 |
| KEGG_PATHWAY | bta04611:Platelet activation | 12 | 0.03 | 5.42E-04 | SYK, NOS3, SRC, AKT2, AKT1, MAPK1, GP1BA, PIK3R1, MAPK14, PRKACA, RHOA, PIK3CG | 0.001092865 |
| KEGG_PATHWAY | bta04923:Regulation of lipolysis in adipocytes | 8 | 0.02 | 5.86E-04 | FABP4, AKT2, INSR, PDE3B, AKT1, PIK3R1, PRKACA, PIK3CG | 0.001163865 |
| KEGG_PATHWAY | bta04150:mTOR signaling pathway | 8 | 0.02 | 9.01E-04 | PDPK1, RHEB, AKT2, AKT1, MAPK1, IGF1, PIK3R1, PIK3CG | 0.001763163 |
| KEGG_PATHWAY | bta04670:Leukocyte transendothelial migration | 11 | 0.03 | 0.001002844 | CDC42, MMP2, RAC2, CTNNA1, PTPN11, PIK3R1, MAPK14, ITGAL, MMP9, RHOA, PIK3CG | 0.001935066 |
| KEGG_PATHWAY | bta05169:Epstein-Barr virus infection | 11 | 0.03 | 0.001144253 | MAPK10, MAPK8, SYK, AKT2, MDM2, AKT1, PIK3R1, MAPK14, ITGAL, JAK3, PIK3CG | 0.002177259 |
| KEGG_PATHWAY | bta04210:Apoptosis | 8 | 0.02 | 0.001214291 | CASP7, AKT2, CASP3, AKT1, XIAP, PIK3R1, PIK3CG, BCL2L1 | 0.002276866 |
| KEGG_PATHWAY | bta05216:Thyroid cancer | 6 | 0.02 | 0.00122984 | RXRB, MAP2K1, RXRA, MAPK1, PPARG, HRAS | 0.002276866 |
| KEGG_PATHWAY | bta00010:Glycolysis / Gluconeogenesis | 8 | 0.02 | 0.001335835 | GPI, TPI1, ALDH2, ADH1C, PCK1, GCK, ADH5, HK1 | 0.002440126 |
| KEGG_PATHWAY | bta00220:Arginine biosynthesis | 5 | 0.01 | 0.001380277 | ARG2, NOS2, NOS3, ARG1, OTC | 0.002488131 |
| KEGG_PATHWAY | bta05202:Transcriptional misregulation in cancer | 13 | 0.04 | 0.001971087 | MMP3, IGF1, MMP9, TGFBR2, IGF1R, RXRB, RXRA, PLAU, MDM2, PPARG, MET, ELANE, BCL2L1 | 0.003465088 |
| KEGG_PATHWAY | bta05166:HTLV-I infection | 17 | 0.05 | 0.001972824 | GSK3B, TGFB2, XIAP, PIK3R1, ITGAL, TGFBR1, IL2, PIK3CG, TGFBR2, LCK, AKT2, CHEK1, AKT1, PRKACA, HRAS, JAK3, BCL2L1 | 0.003465088 |
| KEGG_PATHWAY | bta04144:Endocytosis | 16 | 0.04 | 0.00203215 | HSPA8, ARF1, SRC, RHOA, TGFBR1, EGFR, RAB11A, TGFBR2, IGF1R, CDC42, MDM2, HRAS, RAB5A, FGFR2, HSPA1A | 0.003524108 |
| KEGG_PATHWAY | bta04621:NOD-like receptor signaling pathway | 7 | 0.02 | 0.002193234 | MAPK10, HSP90AA1, MAPK8, CCL5, CASP1, MAPK1, MAPK14 | 0.003755914 |
| KEGG_PATHWAY | bta01200:Carbon metabolism | 10 | 0.03 | 0.00240464 | GPI, G6PD, TPI1, SHMT1, ACADM, AGXT, GCK, ADH5, HK1, ACAT1 | 0.004067107 |
| KEGG_PATHWAY | bta00270:Cysteine and methionine metabolism | 6 | 0.02 | 0.003124991 | MTAP, BHMT, AHCY, CBS, AMD1, SRM | 0.005221022 |
| KEGG_PATHWAY | bta05203:Viral carcinogenesis | 15 | 0.04 | 0.003776045 | SYK, SRC, PIK3R1, RHOA, PIK3CG, CDC42, CDK6, CASP3, CHEK1, MDM2, MAPK1, GRB2, PRKACA, HRAS, JAK3 | 0.006232749 |
| KEGG_PATHWAY | bta05134:Legionellosis | 7 | 0.02 | 0.003873688 | HSPA8, CASP7, ARF1, CASP3, CASP1, HSPA1A | 0.006317801 |
| KEGG_PATHWAY | bta04810:Regulation of actin cytoskeleton | 14 | 0.04 | 0.004243933 | MAP2K1, SRC, PIK3R1, F2, ITGAL, RHOA, EGFR, PIK3CG, CDC42, RAC2, MAPK1, HRAS, FGFR2, FGFR1 | 0.006840222 |
| KEGG_PATHWAY | bta00071:Fatty acid degradation | 6 | 0.02 | 0.004374768 | ALDH2, ADH1C, ACADM, HADH, ADH5, ACAT1 | 0.006969107 |
| KEGG_PATHWAY | bta04360:Axon guidance | 10 | 0.03 | 0.005994036 | CDC42, GSK3B, RAC2, ABL1, MAPK1, HRAS, MET, RHOA, EPHB4, EPHA2 | 0.009438885 |
| KEGG_PATHWAY | bta04750:Inflammatory mediator regulation of TRP channels | 9 | 0.02 | 0.006365759 | MAPK10, MAPK8, SRC, IGF1, PIK3R1, MAPK14, PRKACA, CALM1, PIK3CG | 0.009910329 |
| KEGG_PATHWAY | bta04630:Jak-STAT signaling pathway | 11 | 0.03 | 0.006903695 | STAT1, AKT2, AKT1, GRB2, PTPN11, PIK3R1, JAK2, JAK3, IL2, PIK3CG, BCL2L1 | 0.010627036 |
| KEGG_PATHWAY | bta04725:Cholinergic synapse | 9 | 0.02 | 0.008849266 | MAP2K1, AKT2, AKT1, MAPK1, PIK3R1, JAK2, PRKACA, HRAS, PIK3CG | 0.013470549 |
| KEGG_PATHWAY | bta01100:Metabolic pathways | 47 | 0.13 | 0.009287849 | PNMT, GPI, TPH1, AHCY, FECH, MAOB, ADH1C, SHMT1, AMD1, GBA, ADK, AKR1B1, HMGCR, ABO, ADH5, ACAT1, HK1, SRM, HSD11B1, PNP, IMPA1, ALDH2, CBS, PNPO, ACADM, LTA4H, PCK1, HADH, G6PD, ARG2, TPI1, NOS2, ARG1, NOS3, EPHX2, PLA2G2A, GCK, APRT, DHFR, BHMT, MTAP, NMNAT1, PAH, AGXT, CES1, OTC | 0.013982806 |
| KEGG_PATHWAY | bta05146:Amoebiasis | 9 | 0.02 | 0.009819756 | ARG2, TGFB2, NOS2, ARG1, CASP3, PIK3R1, PRKACA, RAB5A, PIK3CG | 0.014622898 |
| KEGG_PATHWAY | bta01230:Biosynthesis of amino acids | 7 | 0.02 | 0.011259152 | ARG2, TPI1, CBS, ARG1, PAH, SHMT1, OTC | 0.016586063 |
| KEGG_PATHWAY | bta04922:Glucagon signaling pathway | 8 | 0.02 | 0.013092892 | AKT2, PDE3B, AKT1, PCK1, PRKACA, PPARA, CALM1, GCK | 0.019082193 |
| KEGG_PATHWAY | bta05100:Bacterial invasion of epithelial cells | 7 | 0.02 | 0.016360288 | CDC42, SRC, CTNNA1, PIK3R1, MET, RHOA, PIK3CG | 0.023593258 |
| KEGG_PATHWAY | bta05150:Staphylococcus aureus infection | 6 | 0.02 | 0.019917694 | CFD, SELP, C1S, C1R, ITGAL, CFB | 0.028424209 |
| KEGG_PATHWAY | bta04728:Dopaminergic synapse | 9 | 0.02 | 0.020648441 | MAPK10, GSK3B, MAPK8, MAOB, AKT2, AKT1, MAPK14, PRKACA, CALM1 | 0.029163262 |
| KEGG_PATHWAY | bta00350:Tyrosine metabolism | 5 | 0.01 | 0.021089254 | PNMT, ADH1C, MAOB, MIF, ADH5 | 0.029481917 |
| KEGG_PATHWAY | bta05132:Salmonella infection | 7 | 0.02 | 0.022870909 | CDC42, MAPK10, MAPK8, NOS2, CASP1, MAPK1, MAPK14 | 0.030752774 |
| KEGG_PATHWAY | bta04350:TGF-beta signaling pathway | 7 | 0.02 | 0.022870909 | BMP2, TGFB2, MAPK1, BMP7, RHOA, TGFBR1, TGFBR2 | 0.030752774 |
| KEGG_PATHWAY | bta04973:Carbohydrate digestion and absorption | 5 | 0.01 | 0.022896226 | AKT2, AKT1, PIK3R1, PIK3CG, HK1 | 0.030752774 |
| KEGG_PATHWAY | bta00260:Glycine, serine and threonine metabolism | 5 | 0.01 | 0.022896226 | BHMT, MAOB, CBS, SHMT1, AGXT | 0.030752774 |
| KEGG_PATHWAY | bta04022:cGMP-PKG signaling pathway | 10 | 0.03 | 0.025857854 | MAP2K1, NOS3, AKT2, INSR, PDE3B, AKT1, MAPK1, PDE5A, CALM1, RHOA | 0.034393457 |
| KEGG_PATHWAY | bta04310:Wnt signaling pathway | 9 | 0.02 | 0.029546354 | MAPK10, GSK3B, MAPK8, MMP7, CSNK2A1, RAC2, PRKACA, RHOA, PPARD | 0.03892164 |
| KEGG_PATHWAY | bta04320:Dorso-ventral axis formation | 4 | 0.01 | 0.030810529 | MAP2K1, MAPK1, GRB2, EGFR | 0.039980815 |
| KEGG_PATHWAY | bta04540:Gap junction | 7 | 0.02 | 0.030934061 | MAP2K1, SRC, MAPK1, GRB2, PRKACA, HRAS, EGFR | 0.039980815 |
| KEGG_PATHWAY | bta04114:Oocyte meiosis | 8 | 0.02 | 0.032095834 | AR, MAP2K1, MAPK1, PGR, IGF1, PRKACA, CALM1, IGF1R | 0.041094666 |
| KEGG_PATHWAY | bta05206:MicroRNAs in cancer | 13 | 0.04 | 0.033237916 | MAP2K1, TGFB2, MMP9, RHOA, EGFR, CDK6, PLAU, CASP3, ABL1, MDM2, GRB2, HRAS, MET | 0.041878675 |
| KEGG_PATHWAY | bta00380:Tryptophan metabolism | 5 | 0.01 | 0.03331953 | TPH1, ALDH2, MAOB, HADH, ACAT1 | 0.041878675 |
| KEGG_PATHWAY | bta04064:NF-kappa B signaling pathway | 7 | 0.02 | 0.033987462 | ZAP70, CSNK2A1, SYK, PLAU, LCK, XIAP, BCL2L1 | 0.042329839 |
| KEGG_PATHWAY | bta04270:Vascular smooth muscle contraction | 8 | 0.02 | 0.039167979 | MAP2K1, ADORA2A, PLA2G2A, MAPK1, PRKACA, CALM1, RHOA | 0.048220768 |
| KEGG_PATHWAY | bta04921:Oxytocin signaling pathway | 9 | 0.02 | 0.039421358 | MAP2K1, NOS3, SRC, MAPK1, PRKACA, CALM1, HRAS, RHOA, EGFR | 0.048220768 |
| KEGG_PATHWAY | bta05140:Leishmaniasis | 6 | 0.02 | 0.04022598 | TGFB2, NOS2, STAT1, MAPK1, MAPK14, JAK2 | 0.048769551 |
| KEGG_PATHWAY | bta04916:Melanogenesis | 7 | 0.02 | 0.048118896 | GSK3B, MAP2K1, KIT, MAPK1, PRKACA, CALM1, HRAS | 0.057827094 |
| KEGG_PATHWAY | bta04612:Antigen processing and presentation | 6 | 0.02 | 0.049039473 | HSPA8, HSP90AA1, CTSS, CTSB, HSPA1A | 0.058420937 |
| KEGG_PATHWAY | bta05144:Malaria | 5 | 0.01 | 0.051884401 | SELP, TGFB2, ITGAL, MET, SELE | 0.061277266 |
| KEGG_PATHWAY | bta05020:Prion diseases | 4 | 0.01 | 0.052381238 | MAP2K1, CCL5, MAPK1, PRKACA | 0.061335296 |
| KEGG_PATHWAY | bta05014:Amyotrophic lateral sclerosis (ALS) | 5 | 0.01 | 0.054916014 | CASP3, CASP1, MAPK14, RAB5A, BCL2L1 | 0.063758423 |
| KEGG_PATHWAY | bta04730:Long-term depression | 5 | 0.01 | 0.071471488 | MAP2K1, MAPK1, IGF1, HRAS, IGF1R | 0.082282301 |
| KEGG_PATHWAY | bta04146:Peroxisome | 6 | 0.02 | 0.072722264 | NOS2, EPHX2, AGXT, SOD2, CRAT | 0.083024584 |
| KEGG_PATHWAY | bta00620:Pyruvate metabolism | 4 | 0.01 | 0.079477401 | ALDH2, GLO1, PCK1, ACAT1 | 0.089986809 |
| KEGG_PATHWAY | bta04726:Serotonergic synapse | 7 | 0.02 | 0.088722795 | MAP2K1, TPH1, MAOB, CASP3, MAPK1, PRKACA, HRAS | 0.099631336 |
| KEGG_PATHWAY | bta04720:Long-term potentiation | 5 | 0.01 | 0.094304968 | MAP2K1, MAPK1, PRKACA, CALM1, HRAS | 0.105038867 |
| KEGG_PATHWAY | bta04924:Renin secretion | 5 | 0.01 | 0.098410415 | PDE3B, REN, PRKACA, CALM1, CTSB | 0.108727636 |
